# Added-value of mosquito vector breeding sites from street view images in the risk mapping of dengue incidence in Thailand

**DOI:** 10.1371/journal.pntd.0009122

**Published:** 2021-03-08

**Authors:** Myat Su Yin, Dominique J. Bicout, Peter Haddawy, Johannes Schöning, Yongjua Laosiritaworn, Patiwat Sa-angchai

**Affiliations:** 1 Faculty of ICT, Mahidol University, Nakhon Pathom, Thailand; 2 Biomathematics and Epidemiology, EPSP-TIMC, UMR CNRS 5525, Grenoble-Alpes University, VetAgro Sup, Grenoble, France; 3 Laue–Langevin Institute, Theory group, Grenoble, France; 4 Bremen Spatial Cognition Center, University of Bremen, Bremen, Germany; 5 Information Technology Center, Department of Disease Control, Ministry of Public Health, Bangkok, Thailand; 6 Faculty of Tropical Medicine, Mahidol University, Bangkok, Thailand; Centers for Disease Control and Prevention, UNITED STATES

## Abstract

Dengue is an emerging vector-borne viral disease across the world. The primary dengue mosquito vectors breed in containers with sufficient water and nutrition. Outdoor containers can be detected from geotagged images using state-of-the-art deep learning methods. In this study, we utilize such container information from street view images in developing a risk mapping model and determine the added value of including container information in predicting dengue risk. We developed seasonal-spatial models in which the target variable dengue incidence was explained using weather and container variable predictors. Linear mixed models with fixed and random effects are employed in our models to account for different characteristics of containers and weather variables. Using data from three provinces of Thailand between 2015 and 2018, the models are developed at the sub-district level resolution to facilitate the development of effective targeted intervention strategies. The performance of the models is evaluated with two baseline models: a classic linear model and a linear mixed model without container information. The performance evaluated with the correlation coefficients, R-squared, and AIC shows the proposed model with the container information outperforms both baseline models in all three provinces. Through sensitivity analysis, we investigate the containers that have a high impact on dengue risk. Our findings indicate that outdoor containers identified from street view images can be a useful data source in building effective dengue risk models and that the resulting models have potential in helping to target container elimination interventions.

## Author summary

The primary dengue mosquito vectors breed in containers with sufficient water and nutrition. Outdoor containers can be detected from geotagged images using state-of-the-art deep learning methods. Eight breeding site container types in Google street view images are detected using convolutional neural networks. We investigate the added value of including container information from geotagged images in predicting dengue risk. To explain the target variable dengue incidence, weather variables are added to complement the container variable predictors. Linear mixed-effects models are built to account for the effects of spatial and seasonal variation in weather and container variables on the dengue incidence. Evaluation is carried out over three provinces in Thailand: Bangkok, Nakhon Si Thammarat, and Krabi in comparison with classic linear models as well as the mixed effect models without container information. The proposed model with the container information outperforms both baseline models in all three provinces. We further perform sensitivity analysis to investigate the sensitivity of dengue incidence to the changes in the number of containers as well as the improvement in the model performance. This is the first work on dengue risk prediction models using container density information from geotagged images analysis.

## Introduction

Dengue is a mosquito-borne viral infectious disease that has rapidly spread across the world and places tropical countries under a huge socio-economic and disease burden. During the past five decades, the incidence of dengue has increased 30-fold, with the current global incidence estimated at 390 million cases per year [1]. Two species of *Aedes* mosquitoes, *Aedes aegypti* and *Aedes albopictus* are the primary dengue vectors. *Aedes aegypti* has adapted to human habitats and breeds primarily in artificial water containers such as jars, old tires, and flower pots, whereas *Aedes albopictus* tended to breed in natural containers such as tree stumps and coconut shells and to a lesser extent in artificial containers. As potential breeding sites, containers in the environment are routinely surveyed and container elimination is one of the most effective approaches to dengue control. While larval and container surveys can provide crucial information on mosquito vector populations to help in risk prediction and in targeting control efforts, the labor-intensive nature of the surveys limits their practical scope. As a result, studies incorporating larval counts in risk prediction models have been limited in number [2] and scope and indirect proxies such as socioeconomic status and proximity to vector larval development sites are commonly used in risk prediction models [3].

Haddawy et al. [4] presented a novel approach to detect outdoor open containers that constitute potential dengue vector breeding sites in geotagged images and demonstrated the approach on Google street view (GSV) images. Eight of the most common containers are detected in the images using a convolutional neural network. The object recognition algorithm has an accuracy over a test set of images of 0.91 in terms of F-score. The container counts obtained from the GSV images agree well with container counts from available manual surveys. Results from multivariate linear regression relating densities of the eight container types which are considered as the potential breeding sites for both *Aedes aegypti* and *Ae*. *albopictus* to larval survey data show the good prediction of Breteau index values in the dengue season with an R-squared of 0.674. The value of the produced container density information in risk prediction remained an open question.

In this study, we aim to investigate whether container densities obtained from GSV images can be used effectively for dengue risk mapping. Given that GSV images cover only areas along roads and have more limited coverage in rural areas than urban areas, the answer is not obvious. We, therefore, sought to investigate this empirically. Using the container density values from Haddawy et al. [4] and four years of dengue incidence data, we employ a risk prediction approach to determine the added value of container densities obtained from GSV images in predictive models for three provinces in Thailand. We develop Linear Mixed Effects Models (LMER) at the sub-district level, and along with the container density, population data, and metrological covariates are included in the models. Extensive analyses are carried out using incidence data for performance evaluation. The results show that the models with the container variables can predict significantly more accurately than the baseline models. This is the first work to explore the use of container density information obtained from the geotagged images in dengue risk prediction.

### Related work

Weather factors such as the amount of rainfall, humidity, and temperature [2,5–8] were most often incorporated into the dengue risk models. Other than the weather variables, Gross domestic product (GDP) per capita, house conditions [9], and distance to the water source; climatic data such as temperature, rainfall, humidity; environmental data such as vegetation, surface water, and land cover [2] were also commonly used in dengue risk mapping. Entomological indicators such as Breteau Index, House Index, ovitrap Index (at Mexico only), have been considered as proxies for mosquito population in early warning and response systems for dengue outbreak [2,10,11]. In Thailand, Thammapalo et al. [12] reported that larval indices are predictive of the risk for dengue virus transmission. Hettiarachchige et al. [13] made use of surveillance data on *Aedes aegypti* larvae and weather data to build a two-stage risk prediction system for assessing dengue transmission via *Aedes aegypti* mosquitoes on the island of Taiwan. In another study [14], the authors monitored and analyzed the adult female *Ae*. *aegypti* population using vector traps. They compared generalized additive models (GAM) with climate variables including precipitation, temperature, and humidity, and a GAM that additionally included mosquito abundance in the previous week obtained from sticky traps as an explanatory variable. Their results suggest that the adult mosquito infestation index is a good predictor of dengue occurrence. Aryaprema et al. [15] use the Breteau index in predicting dengue risk. They constructed ROC curves to determine the performance of the Breteau indices as predictors of impending dengue outbreaks and to establish a threshold value. In Thailand, seasonal and geographical variations are known to have effects on the infestation of *Aedes* mosquito in the containers in human inhibitions and surroundings [16].

Several existing studies on vector-borne disease risk prediction have used information from GIS images and other remotely-sensed data to represent the type of land cover providing an indirect assessment of appropriateness for vector breeding and survival [6,17–22]. Besides the land cover type, the remotely sensed data have been used to detect the quality of neighborhoods in predicting the dengue risk. Khormi & Kumar [23] used high-resolution GIS images to determine factors such as the density of houses in each neighborhood in each district, the width of streets, and roof area of houses to create a prediction model identifying levels of risk of dengue and to describe the association between dengue cases and the related socio-economic factors. Although remote sensing-based approaches are an efficient tool to collect data on different predictors over large areas, Louis and colleagues [2] showed that reliable predictors for dengue from remote sensing have not yet been established.

Existing risk maps have been developed at low spatial resolution and predicted dengue risk on a country or state scale [2], while only two studies [20,24] were run at the municipality level. The need for risk maps that can deliver information at a spatial precision that would be sufficient to take actions on a finer scale is noted by Louis et al. [2].

## Methods

### Study sites

**Fig 1:**
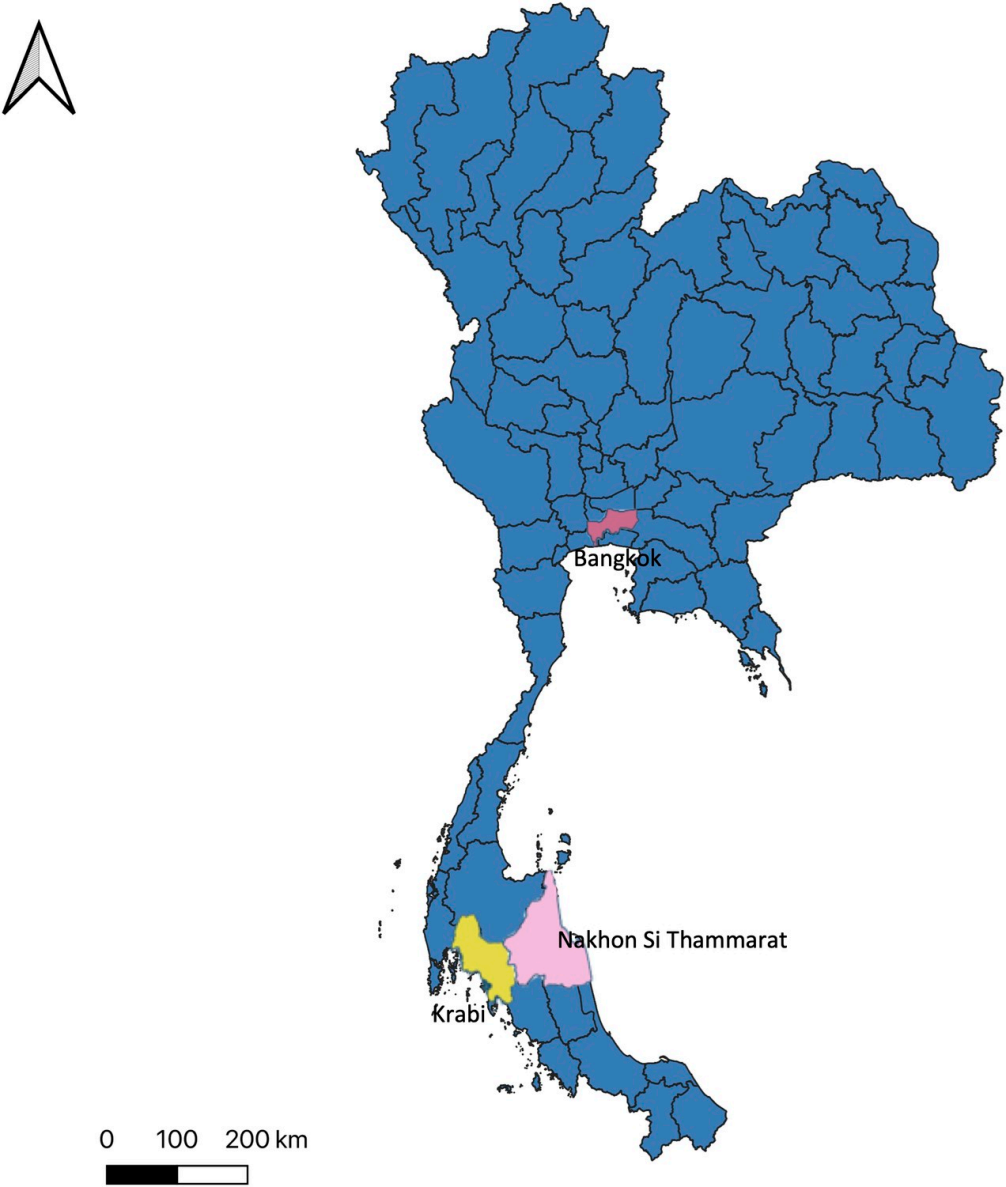
Three provinces highlighted in the Thailand map indicate the locations of Nakhon Si Thammarat (Pink), Krabi (Yellow), and Bangkok (Red). The map in this figure was produced using ArcGIS version 10.4 (Esri, Redlands, CA, USA). Source of shapefile: United Nations Office for the Coordination of Humanitarian Affairs https://data.humdata.org/dataset/thailand-administrative-boundaries.

The study area consists of three provinces in Thailand: Nakhon-Si-Thammarat, Krabi, and Bangkok. Nakhon Si Thammarat is located in southern Thailand (8° 32’ 16.5" N Latitude and 99° 56’ 50.7" E Longitude). The terrain in Nakhon-Si-Thammarat consists of the eastern coastal plain near the Gulf of Thailand, the mountainous area, and the western plain in between two mountains. The overall population in Nakhon-Si-Thammarat is approximately 1.5 million people observed in December 2017 [25]. There are 23 districts and 165 sub-districts. The seasons in Nakhon Si Thammarat are affected by the Gulf of Thailand. The average temperature throughout the entire year is around 27°C [26]. Krabi is located in southern Thailand (8° 5’ 10.68" N Latitude, 98° 54’ 22.62" E Longitude). The western and southern parts of the Krabi border on the Andaman sea. It is primarily lowland, with small monadnocks distributed around the province and mountains from the north to the south. The population in Krabi observed in December 2017 is around 0.47 million people [25]. It consists of 8 districts and 53 sub-districts. Due to the proximity with the sea, the rainfall is quite heavy, and the temperature is steady, with an average of about 28°C throughout the entire year [26]. Bangkok (13° 45’ 22.79" N Latitude, 100° 30’ 6.35" E Longitude) is the capital of Thailand. It consists of lowlands, with the Chao Phraya River flowing through it to the Gulf of Thailand. The population in Bangkok is about 5.7 million people observed in December 2017 [25]. There are 50 districts and 180 sub-districts in total. The average annual temperature in Bangkok is about 28°C [26]. The three provinces in the study are shown in Fig 1.

### Study data

Dengue incidence is the dependent variable in our models. The dengue incidence will be predicted using the information on population, container density from GSV images, and weather variables.

### Dengue incidence

**Fig 2:**
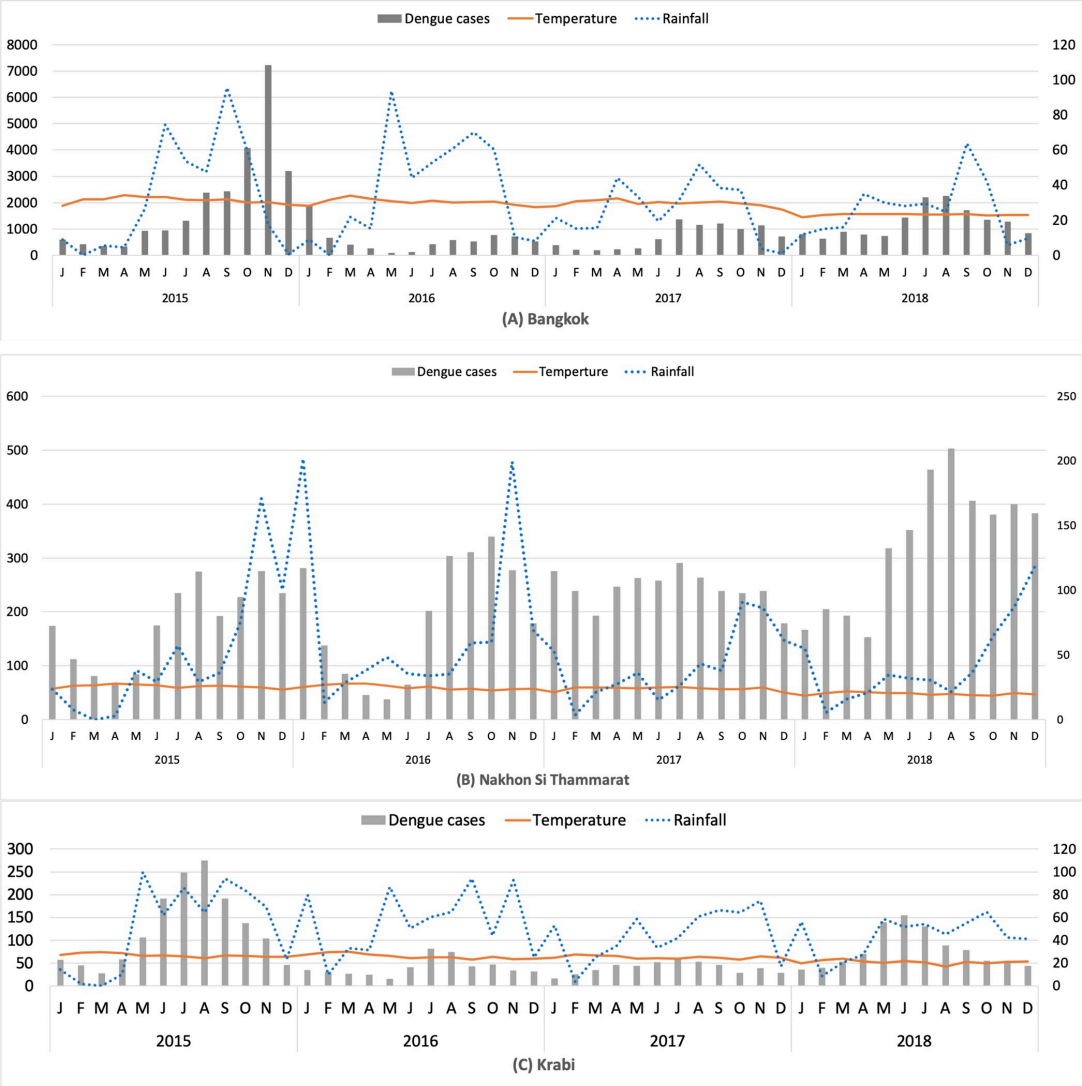
Monthly dengue cases (bar), rainfall (blue dotted line), and temperature (orange line) between 2014 and 2018 in the study areas. The left y-axis represents the number of dengue cases, the right y-axis represents the temperature (LST) and the amount of rainfall (RF).

The dengue incidences were obtained from the dengue surveillance reporting system in Thailand by the Bureau of Epidemiology (BoE), Ministry of Public Health (MoPH) [27]. The number of dengue cases was initially recorded at local hospitals with Form 506 and accumulated at the Bureau of Epidemiology (BoE) for further collation and analysis [27]. A dengue case is defined according to the definitions established by the BoE [27]. For the analysis, we used the dengue incidence per population in each sub-district of Bangkok, Nakhon Si Thammarat, and Krabi provinces from 2015 to 2018. In Fig 2, the monthly dengue incidence, average monthly rainfall, average monthly temperature between 2015 and 2018 are presented for each province.

### Population and weather data

Mosquitoes feed on humans and their breeding sites are directly associated with the population. Population data were obtained from the Thailand National Statistics Office and includes the total population in each subdistrict, as well as the breakdown by age and gender. Climate directly influences mosquito abundance and distribution. Significant correlations have been reported between annual dengue incidence and estimates of *Aedes aegypti* populations at a national scale, using climate-based models [28]. In Thailand, Nakhapakorn and Tripathi [29] reported that the dengue occurrences in Thailand were positively associated with rainfall and negatively associated with temperature and humidity. We obtained rainfall and LST data for the study period from satellite images from the Global Rainfall Map (GSMaP), JAXA global rainfall watch system [30], and MOD11C2 V006 [31]. Monsoon weather patterns predominate in Thailand. The dengue season corresponds to the rainy season, which in Bangkok is from May to October, and in Nakhon Si Thammarat and Krab is from June to November [32].

### Container density

Dengue vector breeding sites consist of open containers of varying sizes that can contain water. The frequency of occurrence and the suitability of containers as breeding sites vary, with ceramic containers generally more suitable than plastic containers. Haddawy et al. [4] detected outdoor open containers which constitute potential dengue vector breeding sites from geotagged Google street view (GSV) images using convolutional neural networks. In this study, we make use of their dataset and provide here a brief description of their approach and their data. Their pipeline to detect and map containers involves image retrieval and object detection. Image retrieval is done by plotting points along each road at 50-meter increments. A distance of 50 meters gives complete image coverage without overlap. At each point, a panoramic view is achieved by retrieving five images 72 degrees apart at a field of view of 75 and a pitch of -15 degrees. Also, the metadata consisting of geo-coordinate and the month and year the image was taken is retrieved. A total of 790,450 GSV images were retrieved from Bangkok, 958,027 from Nakhon Si Thammarat, and 386,819 from Krabi. While there was some variation in the dates of the images, the vast majority were from 2016. It is reasonable to assume that while the location or presence of individual containers may change over time, the total number in an area (absent major intervention efforts) is quite stable, as indicated by a study in Thailand [33]. The percentage image coverage of the three provinces varied considerably. Bangkok had the best image coverage at a mean of 77.06% of total area over all districts, followed by Nakhon Si Thammarat at 8.40%, and Krabi at 7.31%. Coverage tends to be highest in the main population centers and lower in more rural areas.

**Fig 3:**
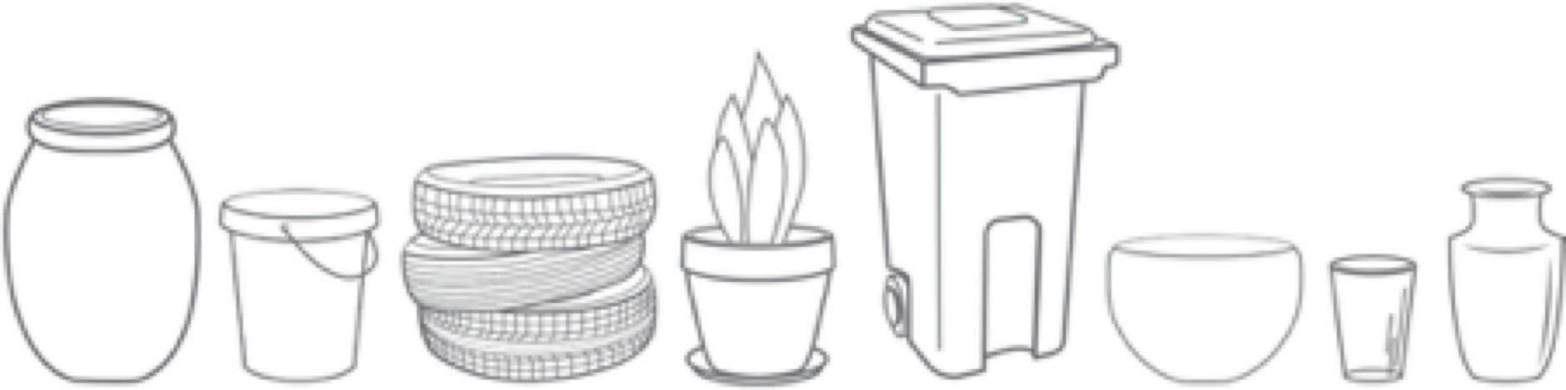
Eight outdoor container types detected from street view images [4], from left to right: Jar, bucket, discarded tire, potted plant, bin, bowl, miscellaneous short open, miscellaneous tall open.

The object detection component of the pipeline detects eight types of containers comprising the most common breeding sites in Thailand: bin, bowl, bucket, jar, potted plant, discarded tire, miscellaneous short open (*Misc_Short*), and miscellaneous tall open (*Misc_Tall*) (Fig 3).

**Fig 4:**
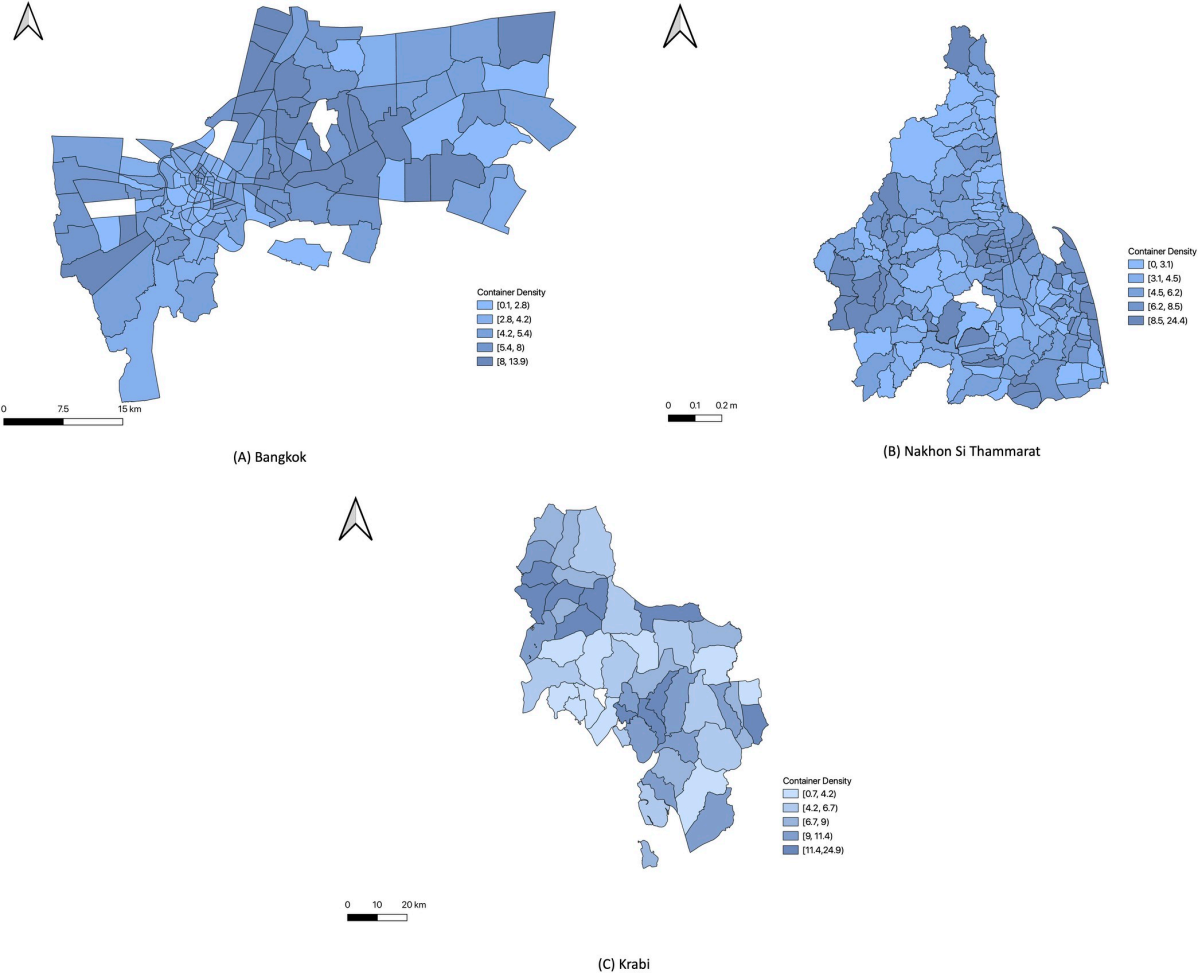
Container density by population (A) Bangkok (B) Nakhon Si Thammarat (C) Krabi province. White color represents sub-districts with no data. The choropleth map in this figure was produced using ArcGIS version 10.4 (Esri, Redlands, CA, USA). Source of shapefile: United Nations Office for the Coordination of Humanitarian Affairs https://data.humdata.org/dataset/thailand-administrative-boundaries.

Their object recognition algorithm has a precision of 0.90, recall of 0.92, and an F-score of 0.91 over a test set of images. A total of 298,391 containers were detected in Bangkok, 84,609 in Nakhon Si Thammarat, and 30,025 in Krabi province. Container density per population (the number of containers/population) was markedly more uniform across the three provinces but showed considerable variation among districts within the provinces (Fig 4). We provide the histograms showing the distribution containers (S1–S3 Figs), the maps showing the distribution of the population (S4–S6 Figs), and the distribution of individual container type density by population in each province in S7–S30 Figs.

Correlations among predictor variables can affect predictive models. Since containers are generated from human activity, some container types tend to occur together and the number of containers in an area is related to the population there. So we examine the correlation between the eight detected containers and the size of the population in sub-districts to determine the relationship between different containers as well as with the population. The correlation between container types and population in the study provinces. In Bangkok, *Misc_Short*, *PottedPlant*, and *tire* container types are strongly correlated (Pearson correlation > 0.9) with *Bin*, *Bowl*, and *Bucket* containers. In Nakhon Si Thammarat, *Bowl*, *Bucket*, *Misc_Short*, *Pottedplant*, and *tire* are strongly correlated with Pearson correlation > 0.9. Similarly in Krabi, *bowl*, *bucket*, *Misc_Short*, *Pottedplant*, and *Tire* are strongly correlated with Pearson correlation > 0.9. We have provided a correlation among containers and the population for all three provinces in S31–S33 Figs.

### Data preparation

Relatively coarse spatial resolutions were considered in previous spatial risk mapping studies, for example at the state level in Singapore [34], at the district level in Brazil [35], and the district level in Thailand [36]. To effectively target dengue in Thailand, the Ministry of Public Health considers a finer resolution at the sub-district level to be appropriate for prediction. But at such a high resolution, there is a significant amount of noise in the number of dengue cases throughout the year. Aggregating them at a large temporal scale can help in mitigating the noise. As noted by Campbell et al. [37], seasonal cycles of dengue disease are observed in every province in Thailand and the public health officials are familiar with the seasonal projections of disease. Besides, we wish to relate the analyses in this study to the results from the previous work [4] which examined the seasonal correlation between container densities and the Breteau index and found a strong correlation during the dengue season. The distribution of *Aedes* vectors was found to be influenced seasonally by breeding outdoors rather than indoors in a study in Thailand [16]. Predictive models at weekly or monthly temporal resolution normally account for lagged effects of weather variables. But with the choice of seasonal resolution, including lag effects is not necessary. Our dataset contains fifteen candidate predictor variables including eight container variables, six weather variables, and a population variable, as shown in Table 1.

**Table 1:** List of candidate variables in the dataset.

Type	Variables	Description
Dengue	Cumulative incidence	Seasonal
Containers	• Bin, Bowl, Bucket, Miscellaneous short open containers (Misc_Short), Jar, Potted plant, Discarded tire (tire), Miscellaneous tall open containers (Misc_Tall)	Kept constant for all seasons
Temperature	• Minimum, maximum, and average daily temperature for dengue season • Minimum, maximum, and average daily temperature for the non-dengue season	Seasonal
Rainfall	• Minimum, maximum, average, total daily rainfall for the dengue season • Minimum, maximum, average, total daily rainfall for the non-dengue season	Seasonal
Population		Kept constant for all seasons.

Since the container types were shown to have a high correlation with one another, we assessed the collinearity between container variables. For the target variable (Dengue incidence), we computed the variance inflation factor (VIF) to measure how much the variance of a regression coefficient is inflated due to multicollinearity between variables. Through experiments, we set the threshold value at two and removed the concerned variables to address the presence of multicollinearity among variables. We used an R function vif() from the *car* package to detect multicollinearity in a regression model for dengue incidence. To find the covariates between different categories of variables, two VIF functions were used separately, one for a GLM model including container variables and a separate GLM model with only the weather variables. The list of container variables and the weather variables in each province after applying the VIF functions are presented in Table 2. From the VIF results, the two container types (Jar and Misc_Tall), and Average rainfall (AVG_RF) were selected for every province, meaning that these variables play a significant role in dengue incidence. All variables in the models, including the target dengue incidence, are standardized by dividing by the standard deviation and log-transformed before building the models. In addition, the container count and dengue incidence variables are first divided by population. The sub-districts with missing weather and dengue incidence values were removed, and 159, 167, and 45 sub-districts are available for Bangkok, Nakhon Si Thammarat, and Krabi province, respectively.

**Table 2:** List of variables for each province.

	Container	Weather
**Bangkok**	• Jar, Misc_Tall	• Maximum temperature (MAX_LST) • Average rainfall (AVG_RF)
**Nakhon Si Thammarat**	• Bin, bowl, bucket, jar, Misc_Tall	• Maximum temperature (MAX_LST) • Average rainfall (AVG_RF) • Total rainfall (SUM_RF)
**Krabi**	• Bowl, bucket, jar, Misc_Tall	• Minimum temperature (MIN_LST) • Average rainfall (AVG_RF)

### Models

To determine the added-value of the containers in the models, we took a step-by-step approach. We started by building a simple generalized linear model (GLM) model to predict dengue incidence using the population and weather data only. The GLM model for each province was fitted using the R’s glm() function. This reference GLM model is referred to as Baseline1 (GLM).

Some sub-districts may have a great probability of disease occurring due to weather, and others may have a lower probability, even after we have accounted for the differences in weather and population traits. These differences are accounted for by incorporating random effects in our second reference model. To capture the mixed effects of the weather, we built the generalized linear mixed models using the *lmer()* function from the *lme4* package in R with the random effect term (1|Year_season). The Year_Season variable represents either the dengue or non-dengue season of each study year and (1|Year_season) is a random intercept which can be different for each season of the study year in the training data. We refer to this reference LMER model as Baseline 2 (LMER).

Next, we built our proposed models by introducing the container variables into Baseline 2 (LMER) models. The container types we considered in this study are man-made and thus likely to be linked to population. Indeed, the analysis of correlation shows the abundance of some container types to be highly correlated to the population (S31–S33 Figs). For each container type identified by the VIF function in the data preparation stage, we added the interaction terms between the container density with the population to the model. With a similar take on seasonal random effects, we incorporated the random effects from among sub-districts with the random effects term with (1| sub-district). With the (1|sub-district) intercept, the model will consider an intercept that is different for each sub-district of the study year in the training data. These LMER models with container information are referred to as (LMER+C) models. Table 3 summarizes the models and the model equations in our study.

**Table 3:** Models and model equations in standard mathematical equations.

Model	Model equation
**General**	$log\left(y_{i}\right)=\beta_{0}+\sum_{j=1}^{3}\beta_{j}x_{i,j}+\sum_{k=1}^{8}\beta_{k+3}c_{i,k}*\left(1+\gamma_{k}x_{i,1}\right)+\sum_{j=1}^{2}b_{0i,j}+\varepsilon_{i}$ *i* = 1,2, ⋯, *Number of sub* − *districts* *y*_*i*_: dengue incidence of sub-district *i* *ε*_*i*_: random error (mean zero) of sub-district *i* *β*_*j*_, *γ*_*k*_ and *b*_*j*_: regression coefficients • Explanatory variables: fixed effect *x*_*i*,1_: population of sub-district *i* *x*_*i*,2_: rainfall of sub-district *i* *x*_*i*,3_: temperature of sub-district *i* *c*_*i*,*k*_: container count of kind *k* (*k* = 1, 2, ⋯ for Jar, Bin, …) of sub-district *i* • Random intercepts *b*_0*i*,1_: year-season (dengue and non-dengue season) of sub-district *i* *b*_0*i*,2_: sub-district *i* *y*_*i*_, *x*_*i*,*j*_ and *c*_*i*,*k*_ are all standardized by the standard deviations over all sub-districts.
**Baseline1 (GLM)**	General model with *β*_*j*>3_ = 0, *γ*_*k*_ = 0 and *b*_0*i*,*j*_ = 0. *R code*: *glm(DengueIncidence ~ Population + Weather variables)*
**Baseline2 (LMER)**	General model with *β*_*j*>3_ = 0, *γ*_*k*_ = 0 and *b*_0*i*,2_ = 0. *R code*: *lmer(DengueIncidence~ Population + Weather variables + (1|Year_Season))*
**LMER+C**	General model *R code*: lmer(DengueIncidence ~ Population + Weather variables + (1|Year_Season) + Container variables * Population + (1|Sub-district))

## Results

Initially, three years of data from 2015 to 2017 were used in both building the model and evaluating the model fitness. We measured the correlation between actual and predicted dengue incidence, R-squared, which represents the proportion of the variance for a target-dependent variable that is explained by the independent variables in a model, and Adjusted Akaike Information Criteria (AICc), which indicates the goodness-of-fit measures for each model. The results are summarized in Table 4. Correlation values range from +1 to -1 and R-squared values range from 0% to 100%, with a value of 100% indicating that all variation in dengue incidence is explained by variation in the independent variables in the model. A low AIC score of a model indicates a simple model with great explanatory predictive power AIC [38].

**Table 4:** Performance of the models on training data (2015–2017) and test data (2018).

	Training data (2015–2017)						Test data (2018)				
	Pearson	Spearman	R-squared	AIC	%increased (Pearson)		Pearson	Spearman	R-squared	%increased (Pearson)	
					Over Baseline1	Over Baseline 2				Over Baseline1	Over Baseline 2
**Bangkok**											
Baseline (GLM)	0.85	0.85	0.72	2377.43			0.83	0.83	0.68		
Baseline (LMER)	0.87	0.92	0.76	1798.70	2.47%		0.87	0.90	0.76	6%	
LMER+C	0.98	0.99	0.96	1563.84	15.45%	12.66%	0.92	0.92	0.85	12%	6%
**Nakhon Si Thammarat**											
Baseline (GLM)	0.76	0.76	0.58	2087.09			0.66	0.66	0.43		
Baseline (LMER)	0.86	0.78	0.75	2109.90	13.13%		0.78	0.64	0.62	18.98%	
LMER+C	0.98	0.97	0.96	2049.32	28.58%	13.66%	0.87	0.70	0.75	32%	11%
**Krabi**											
Baseline (GLM)	0.53	0.53	0.28	765.07			0.55	0.55	0.30		
Baseline (LMER)	0.51	0.62	0.26	717.82	-4.21%		0.70	0.65	0.49	27%	
LMER+C	0.98	0.95	0.96	601.95	84.31%	92.41%	0.78	0.76	0.61	42%	12%

The simple Baseline 1 (GLM) models using only Population and Weather variables achieved moderate performance in Bangkok and Nakhon Si Thammarat over the training data (2015–2017). Performance in Bangkok is characterized by Pearson correlation 0.85, Spearman correlation 0.85, and R-Squared 0.72, while performance in Nakhon Si Thammarat is characterized by Pearson correlation 0.76, Spearman correlation 0.76, and R-Squared 0.58. On the other hand, the Baseline 1 (GLM) model performance is considerably lower in Krabi relative to the other two provinces, with Pearson correlation 0.53, Spearman correlation 0.53, and R-Squared 0.28.

Improvements in performances are observed in Baseline 2 (LMER) models in all three provinces over the training data. We compared the performance between the two baseline models by computing the percentage increase in Spearman rank correlation coefficients. Compared to the Baseline 1 (GLM) models, Baseline 2 (LMER) models have a 2.47% higher correlation in Bangkok and 13.13% in Nakhon Si Thammarat, but 4.21% lower correlation in Krabi. The Baseline 2 (LMER) models have higher R-squared values over the training data in all three provinces, but lower AIC for only Bangkok and Krabi.

Next, we evaluate the LMER+C models. In Bangkok, LMER+C has a 15.45% and 12.66% higher correlation than the Baseline 1 (GLM) model and Baseline 2 (LMER) model, respectively. The LMER+C model correlation increased in Nakhon Si Thammarat (Baseline 1 (GLM): 28.58%, Baseline 2 (LMER): 13.66%), and in Krabi (Baseline 1 (GLM): 84.31%, Baseline 2 (LMER): 92.41%). Also, we can observe that the LMER+C models have the lowest AIC and the highest R-squared compared to the Baseline 1 (GLM) and Baseline 2 (LMER) models in all provinces.

Next, we use the models fitted earlier with three years of training data (2015–2017) and perform prediction on one year of test data (2018). The results are shown in the right half of Table 4. In Bangkok, the Pearson correlation of the LMER+C model is 12% higher than that of the Baseline 1 (GLM) model and 6% higher than the Baseline 2 (LMER) model. In Nakhon Si Thammarat, the Pearson correlation of the LMER+C model is 32% higher than that of the Baseline 1 (GLM) model and 11% higher than the Baseline 2 (LMER) model. In Krabi, the Pearson correlation of the LMER+C model is 42% higher than that of the Baseline 1 (GLM) model and 12% higher than the Baseline 2 (LMER) model.

**Fig 5:**
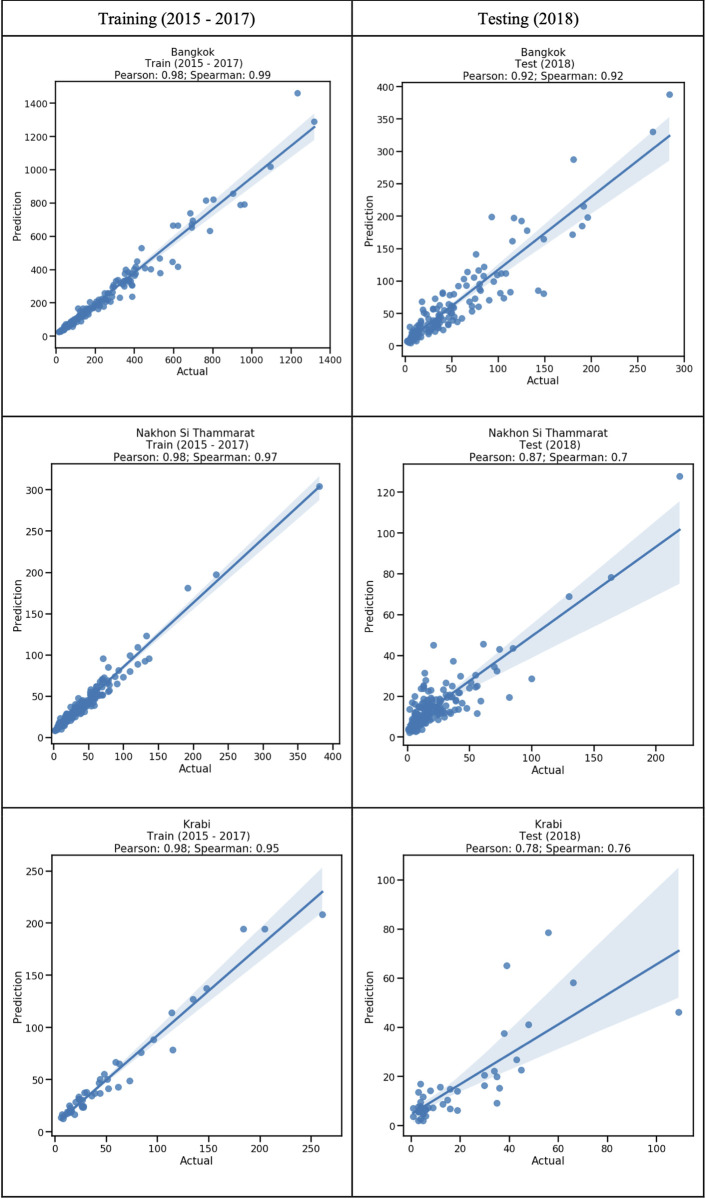
Scatter plots of predicted vs actual values of dengue incidence for LMER+C models for Bangkok, Nakhon Si Thammarat, and Krabi province for the training (2015–2017) and testing (2018) data. The maximum p-value of all panels is less than 2.384x10^-10^. The solid line is a linear trend line which is an indication of the linear (Pearson) correlation between the two variables. (Note: shading shows the 99% confidence interval).

The scatter plots in Fig 5 show the relationship between observed and predicted dengue using the LMER+C model for the three provinces for the training and testing data. There is an overall positive association between observed and predicted counts in all three provinces. With the fitted data, the models are highly predictive for Bangkok (Pearson = 0.98, Spearman = 0.97, p-value < 0.001), for Nakhon Si Thammarat (Pearson = 0.98, Spearman = 0.97, p-value < 0.001) and for Krabi (Pearson = 0.98, Spearman = 0.95, p-value < 0.001). Using the test data, the models are highly predictive for Bangkok (Pearson = 0.91, Spearman = 0.91, p-value < 0.001), and moderately predictive for Nakhon Si Thammarat (Pearson = 0.87, Spearman = 0.70, p-value < 0.001) and for Krabi (Pearson = 0.78, Spearman = 0.76, p-value < 0.001). The distribution of residuals of the developed models was also analyzed. The normal Q-Q plot and residual sequence plots of the study provinces are provided in (S34–S39 Figs). A straight line can be observed in the residual normal probability plot in all three provinces, and the residual sequence plots illustrate the consistent distribution of errors around zero within ± 1.96. These observations indicate a normal distribution of residuals.

**Fig 6:**
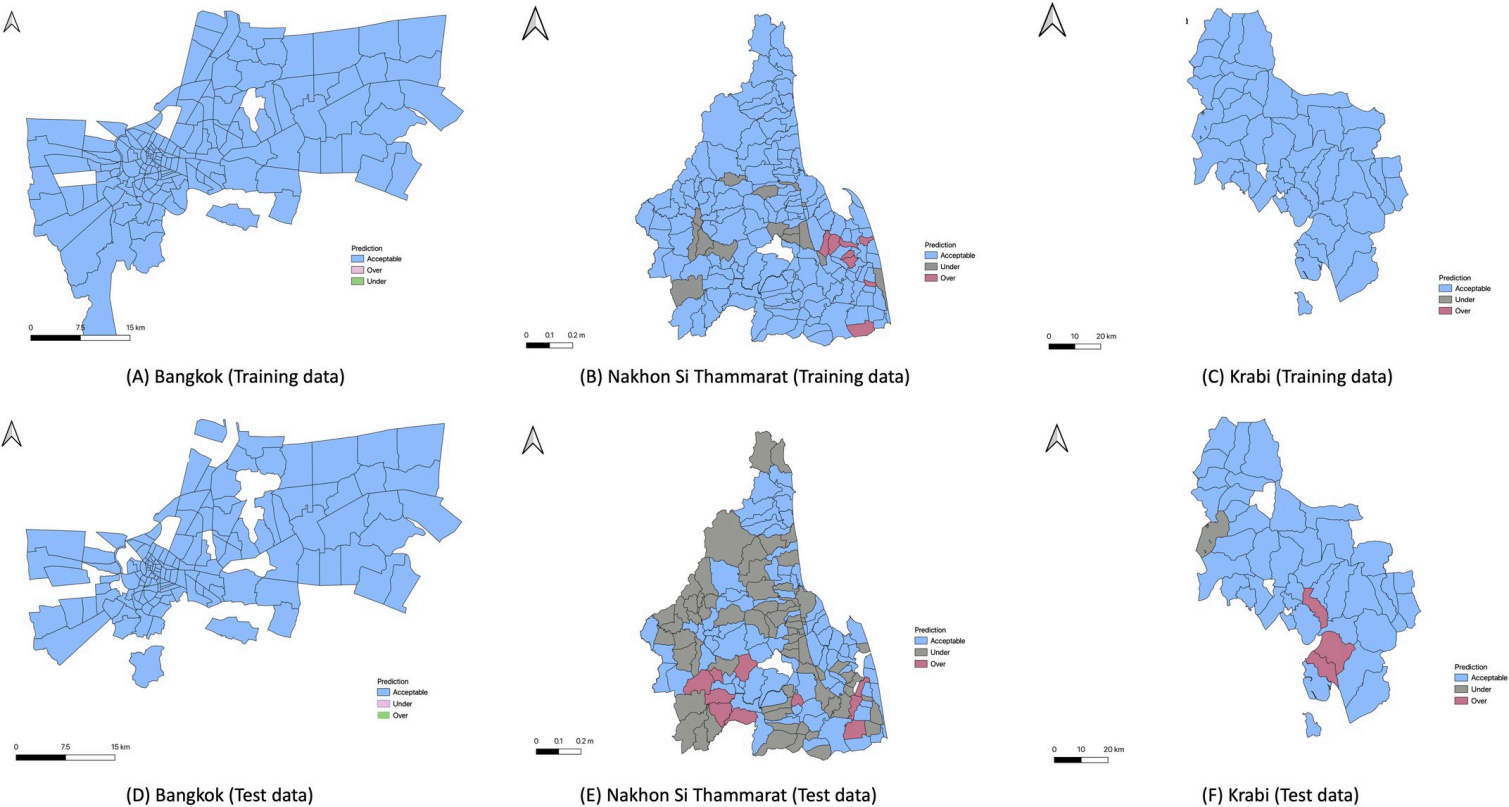
The risk maps of Bangkok, Nakhon Si Thammarat, Krabi for the training (2015–2017), and test (2018) data. White color represents sub-districts with no data. The choropleth map in this figure was produced using ArcGIS version 10.4 (Esri, Redlands, CA, USA). Source of shapefile: United Nations Office for the Coordination of Humanitarian Affairs https://data.humdata.org/dataset/thailand-administrative-boundaries.

Next, we visualize the accuracy of the risk maps over the sub-districts using maps of each province for the training and test data (Fig 6). For each province, the sub-districts are grouped into three categories: *Acceptable*, if the actual value falls within three standard deviations; *Under*, if it is more than and *Over* if it is below three standard deviations of the predicted value.

In Bangkok, the predicted dengue level corresponds well to the observed level in both training and testing data. The same is true of Krabi except for four sub-districts in the testing data. Predictions for the testing data in Nakhon Si Thammarat have the largest number of sub-districts in which dengue incidence is under (69) or over (10) estimated. A plausible explanation is that for the year 2018 the dengue incidence was considerably higher than in any of the three years in the training data, as shown in Fig 2B.

The coefficients of the predictors in the LMER+C models for test data from 2018 are shown in Tables 5–7. We provide the intercepts of the individual sub-districts and season from random effects in Supplements (S1–S3 Tables). The population variable is found to be significant in all provinces. The container-related variables in the models are not always significant. For example, in Nakhon Si Thammarat only the *Jar* container type is found to be significant, however, *Jar* container interaction with the population is not significant.

**Table 5:** The coefficients of the LMER+C model using training data (2015–2017) for Bangkok province. Significant variables with a p-value <0.05 are indicated with boldface.

Predictor	*β*_*j*_	*β*_*j*_ * *γ*_*k*_	SE	95% CI	p-value
Fixed effects					
(Intercept)	1.08		1.01	(-0.91, 3.06)	0.297
**Population**	2.2		0.2	(1.81, 2.59)	< 0.001
MAX_LST	-0.1		0.06	(-0.22, 0.03)	0.126
**AVG_RF**	0.97		0.23	(0.52, 1.41)	< 0.001
**Jar**	0.73		0.27	(0.20, 1.26)	0.008
**Misc_Tall**	-0.21		0.1	(-0.40, -0.01)	0.04
Jar * Population		-1.28	1.01	(-1.93, -0.63)	< 0.001
Population * Misc_Tall		0.79	0.27	(0.32, 1.26)	0.001
Random effects	Std of *b*_0,*j*_				
Subdistrict (intercept)	0.36				
Year_Season (intercept)	1.45				

Note: Std stands for standard deviation.

**Table 6:** The coefficients of the LMER+C model using training data (2015–2017) for Nakhon Si Thammarat province. Significant variables with a p-value <0.05 are indicated with boldface.

Predictor	*β*_*j*_	*β*_*j*_ * *γ*_*k*_	SE	95% CI	p-value
Fixed effects					
**(Intercept)**	2.16		0.59	(1.00, 3.32)	<0.001
**Population**	1.29		0.2	(0.89, 1.68)	<0.001
**MAX_LST**	-0.17		0.05	(-0.27, -0.07)	<0.001
AVG_RF	0.05		0.07	(-0.08, 0.18)	0.494
SUM_RF	0.14		0.08	(-0.01,0.29)	0.196
Bin	-0.09		0.34	(-0.76, 0.58)	0.792
Bowl	0.05		0.29	(-0.53, 0.63)	0.864
Bucket	0.19		0.44	(-0.66, 1.05)	0.66
**Jar**	-0.34		0.17	(-0.68, 0.00)	0.049
Misc_Tall	-0.27		0.23	(-0.72, 0.18)	0.249
Bin * Population		0.36	0.39	(-0.41, 1.14)	0.359
Population * Bowl		-0.29	0.37	(-1.02, 0.44)	0.435
Population * Bucket		0.04	0.48	(-0.91, 0.99)	0.927
Population * Jar		0.16	0.24	(-0.30, 0.63)	0.487
Population * Misc_Tall		0.51	0.3	(-0.07, 1.10)	0.089
Random effects	Std of *b*_0,*j*_				
Subdistrict (intercept)	0.29				
Year_Season (intercept)	0.13				

Note: Std stands for standard deviation.

**Table 7:** The coefficients of the LMER+C models using training data (2015–2017) for Krabi province. Significant variables with a p-value <0.05 are indicated with boldface.

Predictor	*β*_*j*_	*β*_*j*_ * *γ*_*k*_	SE	95% CI	p-value
Fixed effects					
(Intercept)	-0.12		1.62	(-3.28, 3.05)	0.943
**Population**	2.68		0.71	(1.29, 4.07)	<0.001
MIN_LST	-0.09		0.11	(-0.29, 0.12)	0.419
AVG_RF	0.17		0.15	(-0.13, 0.47)	0.274
Bowl	-3.01		1.71	(-6.37, 0.35)	0.087
Bucket	1.33		2.17	(-2.93, 5.58)	0.545
Jar	2.45		1.83	(-1.14, 6.05)	0.19
Misc_Tall	1.37		1.24	(-1.06, 3.80)	0.277
Bowl * Population		3.31	1.6	(0.18, 6.43)	0.046
Population * Bucket		-2.26	1.95	(-6.08, 1.55)	0.253
Population * Jar		-3.15	1.67	(-6.43, 0.12)	0.067
Population * Misc_Tall		-0.91	1.12	(-3.10, 1.28)	0.421
Random effect	Std of *b*_0,*j*_				
Subdistrict (intercept)	0.44				
Year_Season (intercept)	0.55				

Note: Std stands for standard deviation.

The *Population* variable is significant in the LMER+C models of all three provinces. Significant weather variables are the *Average rainfall (AVG_RF)* in Bangkok and the *Maximum temperature (MAX_LST)* in Nakhon Si Thammarat. Surprisingly, only a few container-related variables are significant across the three provinces. All container-related variables are significant in Bangkok, whereas only the *Jar* variable is significant in Nakhon Si Thammarat. In Krabi, only *Bowl* container type interaction with the *Population* is significant. Based on the significant variables in the model, we can know the relative importance of identified outdoor containers responsible for the dengue incidences. With the significant variables identified, the next step is to quantify the importance of each.

### Sensitivity analysis

Sensitivity analysis is commonly employed to quantify the importance of each of a model’s parameters on its behavior and to determine the robustness of model predictions to variations in parameter values. In epidemiology, it is often used to discover parameters that have a high impact on disease incidence and should be targeted by intervention strategies [39]. Since we are interested in determining the impact of the different types of containers, we measure the relative change in a dengue incidence as container parameters change.

Similar to the normalized sensitivity index presented by Rodrigues et al. [39], the sensitivity index (S) is the proportion of decrease in the dengue incidence after decreasing the number of containers in each sub-district and is calculated as

$${\text{S}}_{\text{z}}=100*\left[\text{K}\left(\text{c}\right)—\text{K}\left(\text{z}\right)\right]/\text{K}\left(\text{c}\right)$$

where

- K(c) is the dengue incidence predicted with the significant container variables from the LMER+C model using the actual container counts, and
- K(z) is the dengue incidence predicted with the significant container variables from the LMER+C model after removing z-percent uniformly from those container types.

In computing S, we use only the variables with significant coefficients from the LMER+C models. We compute S twice, with z = 50 to simulate the 50% removal of identified containers and with z = 100 to simulate the complete removal of identified containers for each sub-district. Since setting z = 100 corresponds to predicting risk without container information, the S_100_ indices provide a way to quantify the added value of the container information in the LMER+C model.

**Fig 7:**
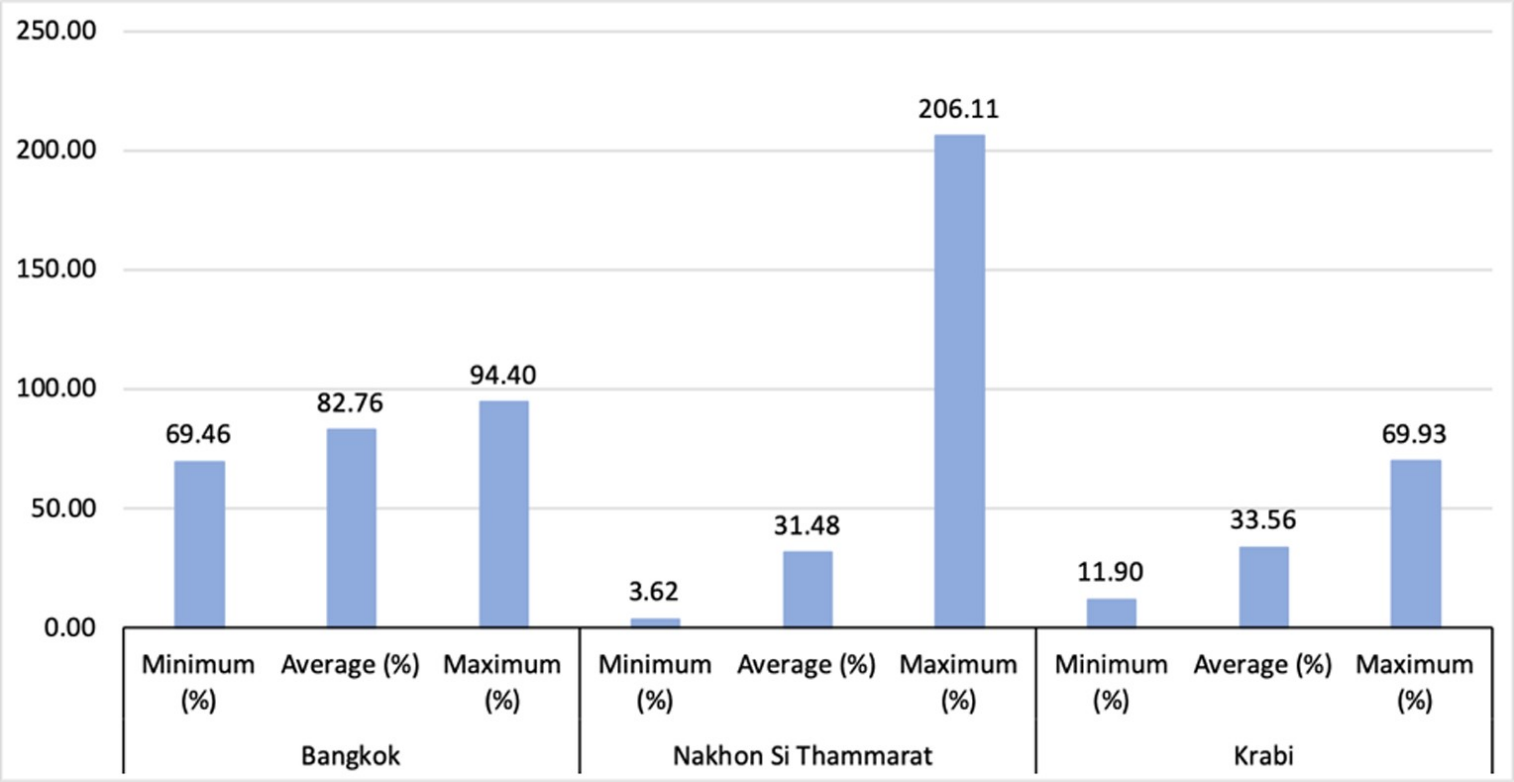
The ranges of sensitivity index (S_100_) for each province.

The range of percentage reduction in dengue incidence concerning the container variables (S_100_) in the LMER+C models is shown in Fig 7. In Bangkok, 140 sub-districts are identified with dengue cases contributed from identified outdoor containers; among them, 63 sub-districts have less than average percentage of cases (82.76%) contributed from the containers. Similarly, in Nakhon Si Thammarat 141 sub-districts have dengue incidence contributed from containers; among them, 97 sub-districts have less than the average percentage of cases (31.48%) contributed from the containers. The proportions of decrease in dengue cases more than 100% for Nakhon Si Thammarat are due to negative predictions for two sub-districts when using the LMER+C model with only significant container variables. Similarly, in Krabi 41 sub-districts have dengue incidence contributed from containers, among them, 23 sub-districts have less than an average percentage of cases (33.56%) contributed from the containers. The remaining cases may be contributed from the other predictors such as temperature and rainfall, and other factors such as indoor containers which are not considered in our models.

**Fig 8:**
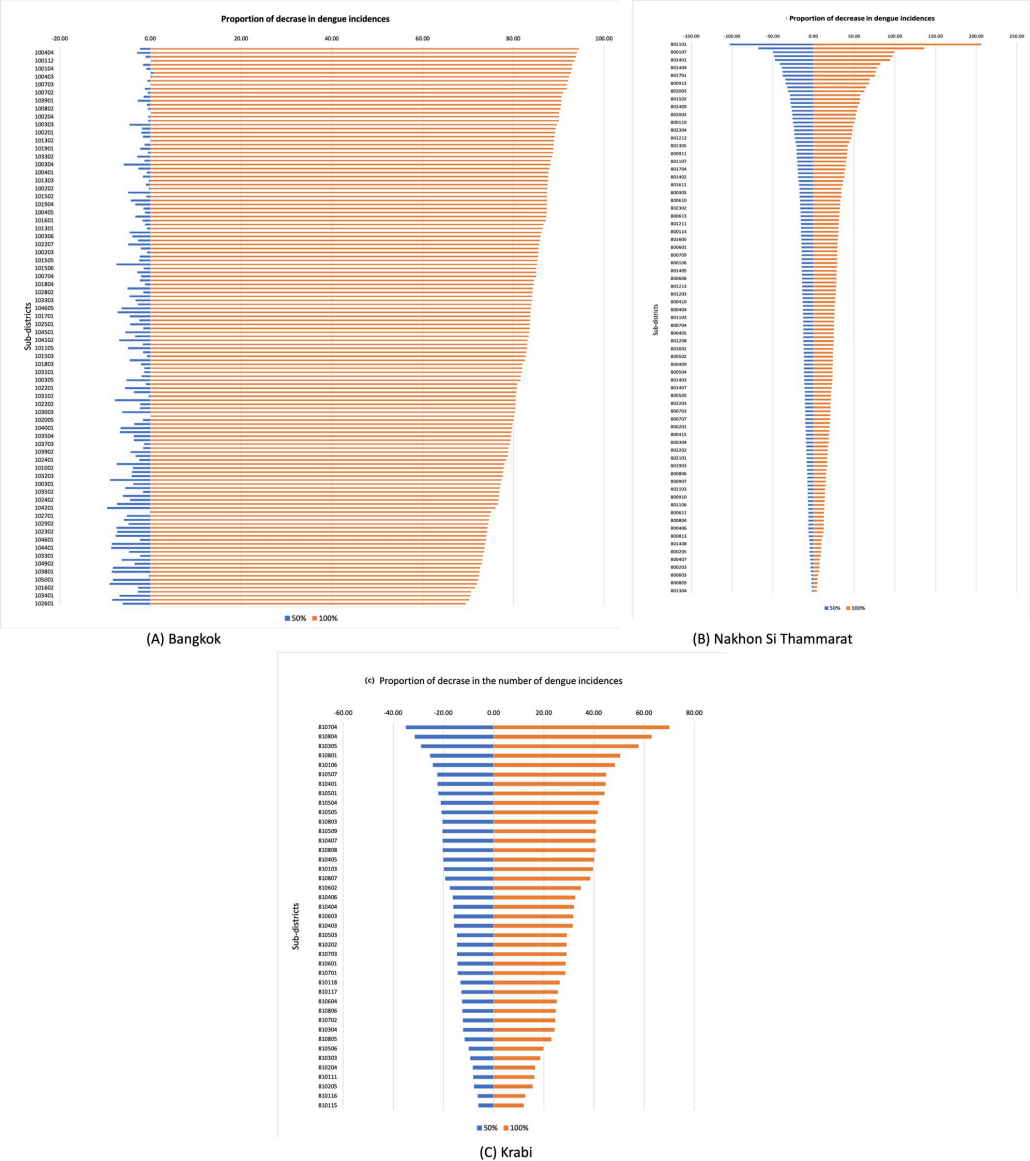
The proportion of decrease in dengue cases in (A) Bangkok, (B) Nakhon Si Thammarat, (C) Krabi province.

Fig 8 shows the sensitivity index for the sub-districts after eliminating the number of containers by 50% (blue color) and 100% (orange color) in Bangkok, Nakhon Si Thammarat, and Krabi province. As expected, the sub-districts with the high container densities are more sensitive to the reduction in containers. In Bangkok and Krabi, the sensitivity index is much higher for S_100_ (complete removal of outdoor containers) than for S_50_ (50% removal of identified outdoor containers). A plausible explanation is that when 50% of the containers are removed, that may still leave a good number of sites where the vectors can breed, whereas when all detected outdoor containers are removed, that leaves only indoor containers and undetected outdoor containers. Meanwhile, the change in sensitivity index in Nakhon Si Thammarat is roughly linear, going from S_50_ to S_100_. This is likely due to the insignificance of interaction between population and container terms in the model for that province.

## Discussion

Vector abundance is an important factor in determining dengue risk, particularly for *Aedes* mosquitoes which are more adapting to the urban environment and are more widely dispersed now than at any time in the past [40]. Traditional vector control approaches using larval and container surveys provide an estimate of the number of vectors but they are costly, labor-intensive, and are not feasible to implement for the large area, and are not sustainable in long term [2,41,42]. Studies related to vector-borne disease modeling often include the proxies of mosquito breeding or resting sites based on the vector-knowledge reviewed in the literature [43,44], survey data [13], and Breteau Index [15]. In this paper, we conducted a proof-of-concept study to determine the effects of outdoor container information detected from geotagged images in risk models.

*Aedes* mosquitoes breed in containers and are closely associated with humans. They are highly anthropophilic, and predominantly found in densely populated urban areas. Being a necessary driver for dengue transmission, population dynamics are often considered in disease transmission [45]. Besides population, strong associations with dengue incidences and weather variables have been shown in studies. Weather predictors predominantly used in the existing studies related to dengue are rainfall and temperature [5–8]. Similar to existing studies’ findings, the results from our baseline models confirm that dengue risk can be predicted reasonably well using a simple GLM model with only population, weather variables consisting of temperature, and rainfall variables. Weather variables have non-linear relationships with the dengue cases [46–48] and have delayed effects on the number of dengue cases [49,50]. To determine the effects of container information in the models, our models were built at the level of dengue and non-dengue seasons (two points per year) and we used a simple linear model for temperature and rainfall. We consider the effect of the changes in weather and their impact on dengue cases as background information. LMERC+C model results indicated that the inclusion of container information from street-view images in the model with weather variables could help in predicting the risk of dengue. The current models could be improved with the inclusion of non-linear relationships between weather and dengue cases.

To our best knowledge, this is the first study that uses the container counts from street-view images in dengue risk mapping. Containers are the major breeding sites of the dengue vector and the *Aedes* mosquitoes tend to remain close to their breeding sites, container counts can be highly indicative of local vector populations. We built the LMER+C models by including the container density variables, their interactions with the population to the models as well as intercepts for each sub-district by defining the sub-district codes as random effects variables. The results indicate that weather variables alone may be one of several necessary to determine risk but insufficient factors, confirming that the prediction of future dengue risk should not rely exclusively on climatic factors [51]. The container-population interaction variables are found to be significant in the Bangkok and Krabi models, meaning that two sub-districts with the same container / population ratio but with different populations and number of containers would not result in the same impact. Fig 8, also confirms that the effect of containers varies from one sub-district to another.

Both natural and artificial containers of all sorts near human habitats have the potential to become mosquito breeding sites. The outdoor container information from street-view images in the LMER+C model acts as the proxy representing the vector abundance that other models currently obtained from manual survey data. The findings in the present study have shown the significant contribution of container information in the dengue transmission and distribution pattern. Vector-control strategies usually focus on reducing sources of *Aedes* larva and pupa habitats [52]. One of the World Health Organization strategies to control *Ae*. *aegypti* is by eliminating sources of *Aedes such as* container habitats that could become the breeding sites [52,53]. In practice, the effectiveness of the vector-control strategies can neither be predicted nor measured until the number of dengue cases is collected and reported at the end of the study season. Through sensitivity analysis, we characterize the response of model outputs to container parameter variation in the LMER+C models. Sensitivity indices of the sub-districts were computed by varying the number of identified outdoor containers to simulate the effect of reduction in breeding sites on dengue cases. One important aspect of such a model to a public health decision maker is its ability to predict dengue so that areas with a high risk of dengue can be prioritized for intervention, thereby reducing the incidence and possible epidemic. The mapping of such areas can be done using the container detection pipeline [4] with the risk mapping models shown in this study. Our models can also be used for the Early Warning Alert and Response System (EWARS) [54] and other surveillance actions periodically. Areas with high container density which are possibly associated with large vector mosquito populations can be identified, so that preventive actions, such as insecticide fogging, application of larvicides, and elimination of the breeding sites can be conducted to prevent the incidence of dengue fever.

It is important to note that the percentage increase in Pearson correlations is reduced in the prediction of dengue incidence with the test data. One possible reason for the lower performance with test data compared to the training data is contributed from the *Year_season* random effect variables. While the global random effect from the *Year_Season* variable well explained the dengue incidence occurrence, the individual intercepts obtained from each study year in the training data do not apply to the new (unseen) year grouping variable in the test data. Consequently, the yearly intercepts are not made use of in predicting the unseen test year.

Our approach to using GSV images to obtain container counts has limitations in terms of temporal and spatial coverage. GSV images are often two to three years old. In our study, this was not an issue since we were also making use of historical dengue incidence data. For use of our approach in practice, we assume that while individual containers may move or be destroyed over time, the total count in a district or sub-district is relatively stable over time. Phuanukoonnon et al. [32] studied the mean numbers of containers in rural and urban areas and found that for over 10 years (between 1992–2005), the number of water storage containers in Thailand has not changed. Alternatively, fresher images could be obtained through some of the crowdsourcing tools such as Mapillary (www.mapillary.com) and OpenStreetCam (openstreetcam.org) or the targeted use of drones [55]. Drones could be a particularly useful approach for collecting data after interventions to eliminate containers.

Furthermore, GSV images do not cover every area on the map since the images are usually captured by cars driving through the streets and thus the containers in isolated and inaccessible areas from the roads, as well as indoor containers are not considered in our study. In future work, we will seek to incorporate such container counts by estimating them through the classification of housing types and socioeconomic status of neighborhoods from the street view images [56]. Drones can also play a role here by enabling the collection of images from outdoor areas that do not lie along roads.

Distribution pattern of dengue cases and its interaction with weather, containers, and spatial factors can be used for modeling of interactive dengue surveillance and effective management system not merely in study areas but also in the other highly reported dengue cases areas in Thailand or elsewhere. The present study will stimulate further discussion on how to strengthen current existing dengue prevention and control actions with vector-abundance indicators which were previously not available without expensive monitoring and field evaluation.

## Supporting information

S1 Fig Bangkok–Distribution of container. (TIF)

S2 Fig Nakhon Si Thammarat—Distribution of containers. (TIF)

S3 Fig Krabi—Distribution of containers. (TIF)

S4 Fig Bangkok–Population. The map in this figure was produced using ArcGIS version 10.4 (Esri, Redlands, CA, USA). Source of shapefile: United Nations Office for the Coordination of Humanitarian Affairs https://data.humdata.org/dataset/thailand-administrative-boundaries.
(TIF)

S5 Fig Nakhon Si Thammarat Population. The map in this figure was produced using ArcGIS version 10.4 (Esri, Redlands, CA, USA). Source of shapefile: United Nations Office for the Coordination of Humanitarian Affairs https://data.humdata.org/dataset/thailand-administrative-boundaries.
(TIF)

S6 Fig Krabi Population. The map in this figure was produced using ArcGIS version 10.4 (Esri, Redlands, CA, USA). Source of shapefile: United Nations Office for the Coordination of Humanitarian Affairs https://data.humdata.org/dataset/thailand-administrative-boundaries.
(TIF)

S7 Fig Bangkok—Container density by the population (Bowl). The map in this figure was produced using ArcGIS version 10.4 (Esri, Redlands, CA, USA). Source of shapefile: United Nations Office for the Coordination of Humanitarian Affairs https://data.humdata.org/dataset/thailand-administrative-boundaries.
(TIF)

S8 Fig Nakhon Si Thammarat—Container density by the population (Bowl). The map in this figure was produced using ArcGIS version 10.4 (Esri, Redlands, CA, USA). Source of shapefile: United Nations Office for the Coordination of Humanitarian Affairs https://data.humdata.org/dataset/thailand-administrative-boundaries.
(TIF)

S9 Fig Krabi—Container density by the population (Bowl). The map in this figure was produced using ArcGIS version 10.4 (Esri, Redlands, CA, USA). Source of shapefile: United Nations Office for the Coordination of Humanitarian Affairs https://data.humdata.org/dataset/thailand-administrative-boundaries.
(TIF)

S10 Fig Bangkok—Container density by the population (Bin). The map in this figure was produced using ArcGIS version 10.4 (Esri, Redlands, CA, USA). Source of shapefile: United Nations Office for the Coordination of Humanitarian Affairs https://data.humdata.org/dataset/thailand-administrative-boundaries.
(TIF)

S11 Fig Nakhon Si Thammarat—Container density by the population (Bin). The map in this figure was produced using ArcGIS version 10.4 (Esri, Redlands, CA, USA). Source of shapefile: United Nations Office for the Coordination of Humanitarian Affairs https://data.humdata.org/dataset/thailand-administrative-boundaries.
(TIF)

S12 Fig Krabi—Container density by the population (Bin). The map in this figure was produced using ArcGIS version 10.4 (Esri, Redlands, CA, USA). Source of shapefile: United Nations Office for the Coordination of Humanitarian Affairs https://data.humdata.org/dataset/thailand-administrative-boundaries.
(TIF)

S13 Fig Bangkok—Container density by the population (Bucket). The map in this figure was produced using ArcGIS version 10.4 (Esri, Redlands, CA, USA). Source of shapefile: United Nations Office for the Coordination of Humanitarian Affairs https://data.humdata.org/dataset/thailand-administrative-boundaries.
(TIF)

S14 Fig Nakhon Si Thammarat—Container density by the population (Bucket). The map in this figure was produced using ArcGIS version 10.4 (Esri, Redlands, CA, USA). Source of shapefile: United Nations Office for the Coordination of Humanitarian Affairs https://data.humdata.org/dataset/thailand-administrative-boundaries.
(TIF)

S15 Fig Krabi—Container density by the population (Bucket). The map in this figure was produced using ArcGIS version 10.4 (Esri, Redlands, CA, USA). Source of shapefile: United Nations Office for the Coordination of Humanitarian Affairs https://data.humdata.org/dataset/thailand-administrative-boundaries.
(TIF)

S16 Fig Bangkok—Container density by the population (Jar). The map in this figure was produced using ArcGIS version 10.4 (Esri, Redlands, CA, USA). Source of shapefile: United Nations Office for the Coordination of Humanitarian Affairs https://data.humdata.org/dataset/thailand-administrative-boundaries.
(TIF)

S17 Fig Nakhon Si Thammarat—Container density by the population (Jar). The map in this figure was produced using ArcGIS version 10.4 (Esri, Redlands, CA, USA). Source of shapefile: United Nations Office for the Coordination of Humanitarian Affairs https://data.humdata.org/dataset/thailand-administrative-boundaries.
(TIF)

S18 Fig Krabi—Container density by the population (Jar). The map in this figure was produced using ArcGIS version 10.4 (Esri, Redlands, CA, USA). Source of shapefile: United Nations Office for the Coordination of Humanitarian Affairs https://data.humdata.org/dataset/thailand-administrative-boundaries.
(TIF)

S19 Fig Bangkok—Container density by the population (Misc_Short). The map in this figure was produced using ArcGIS version 10.4 (Esri, Redlands, CA, USA). Source of shapefile: United Nations Office for the Coordination of Humanitarian Affairs https://data.humdata.org/dataset/thailand-administrative-boundaries.
(TIF)

S20 Fig Nakhon Si Thammarat—Container density by the population (Misc_Short). The map in this figure was produced using ArcGIS version 10.4 (Esri, Redlands, CA, USA). Source of shapefile: United Nations Office for the Coordination of Humanitarian Affairs https://data.humdata.org/dataset/thailand-administrative-boundaries.
(TIF)

S21 Fig Krabi—Container density by the population (Misc_Short). The map in this figure was produced using ArcGIS version 10.4 (Esri, Redlands, CA, USA). Source of shapefile: United Nations Office for the Coordination of Humanitarian Affairs https://data.humdata.org/dataset/thailand-administrative-boundaries.
(TIF)

S22 Fig Bangkok—Container density by the population (Misc_Tall). The map in this figure was produced using ArcGIS version 10.4 (Esri, Redlands, CA, USA). Source of shapefile: United Nations Office for the Coordination of Humanitarian Affairs https://data.humdata.org/dataset/thailand-administrative-boundaries.
(TIF)

S23 Fig Nakhon Si Thammarat—Container density by the population (Misc_Tall). The map in this figure was produced using ArcGIS version 10.4 (Esri, Redlands, CA, USA). Source of shapefile: United Nations Office for the Coordination of Humanitarian Affairs https://data.humdata.org/dataset/thailand-administrative-boundaries.
(TIF)

S24 Fig Krabi—Container density by the population (Misc_Tall). The map in this figure was produced using ArcGIS version 10.4 (Esri, Redlands, CA, USA). Source of shapefile: United Nations Office for the Coordination of Humanitarian Affairs https://data.humdata.org/dataset/thailand-administrative-boundaries.
(TIF)

S25 Fig Bangkok—Container density by the population (Potted plants). The map in this figure was produced using ArcGIS version 10.4 (Esri, Redlands, CA, USA). Source of shapefile: United Nations Office for the Coordination of Humanitarian Affairs https://data.humdata.org/dataset/thailand-administrative-boundaries.
(TIF)

S26 Fig Nakhon Si Thammarat—Container density by the population (Potted plants). The map in this figure was produced using ArcGIS version 10.4 (Esri, Redlands, CA, USA). Source of shapefile: United Nations Office for the Coordination of Humanitarian Affairs https://data.humdata.org/dataset/thailand-administrative-boundaries.
(TIF)

S27 Fig Krabi—Container density by the population (Potted plants). The map in this figure was produced using ArcGIS version 10.4 (Esri, Redlands, CA, USA). Source of shapefile: United Nations Office for the Coordination of Humanitarian Affairs https://data.humdata.org/dataset/thailand-administrative-boundaries.
(TIF)

S28 Fig Bangkok—Container density by the population (Tire). The map in this figure was produced using ArcGIS version 10.4 (Esri, Redlands, CA, USA). Source of shapefile: United Nations Office for the Coordination of Humanitarian Affairs https://data.humdata.org/dataset/thailand-administrative-boundaries.
(TIF)

S29 Fig Nakhon Si Thammarat—Container density by the population (Tire). The map in this figure was produced using ArcGIS version 10.4 (Esri, Redlands, CA, USA). Source of shapefile: United Nations Office for the Coordination of Humanitarian Affairs https://data.humdata.org/dataset/thailand-administrative-boundaries.
(TIF)

S30 Fig Krabi—Container density by the population (Tire). The map in this figure was produced using ArcGIS version 10.4 (Esri, Redlands, CA, USA). Source of shapefile: United Nations Office for the Coordination of Humanitarian Affairs https://data.humdata.org/dataset/thailand-administrative-boundaries.
(TIF)

S31 Fig Bangkok—Correlation between container density and population. (TIF)

S32 Fig Nakhon Si Thammarat—Correlation between container density and population. (TIF)

S33 Fig Krabi—Correlation between container density and population. (TIF)

S34 Fig Bangkok—Residual Q-Q plot (using training data). (TIF)

S35 Fig Bangkok—Residuals by sub-districts plot (using training data). (TIF)

S36 Fig Nakhon Si Thammarat—Residual Q-Q plot (using training data). (TIF)

S37 Fig Nakhon Si Thammarat–Residuals by sub-districts plot (using training data). (TIF)

S38 Fig Krabi—Residual Q-Q plot (using training data). (TIF)

S39 Fig Krabi—Residuals by sub-districts plot (using training data). (TIF)

S1 Table Coefficients for random effect variables (Bangkok). (DOCX)

S2 Table Coefficients for random effect variables (Nakhon Si Thammarat). (DOCX)

S3 Table Coefficients for random effect variables (Krabi). (DOCX)
