# Supplementary figures and images for: Added-value of mosquito vector breeding sites from street view images in the risk mapping of dengue incidence in Thailand
Source: PLoS Negl Trop Dis. 2021 Mar 8;15(3):e0009122. doi: 10.1371/journal.pntd.0009122 (PMC7971869; doi:10.1371/journal.pntd.0009122)

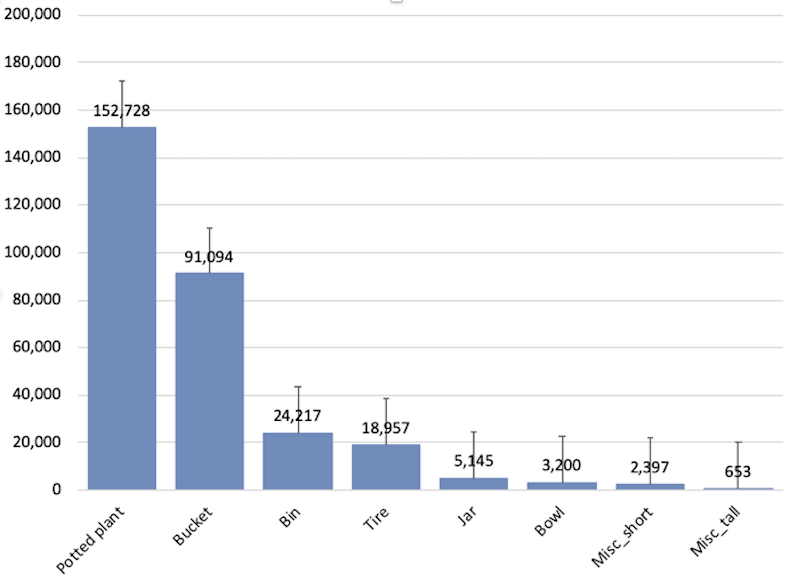

Supplement: S1 Fig — (TIF) [file pntd.0009122.s001.tif]

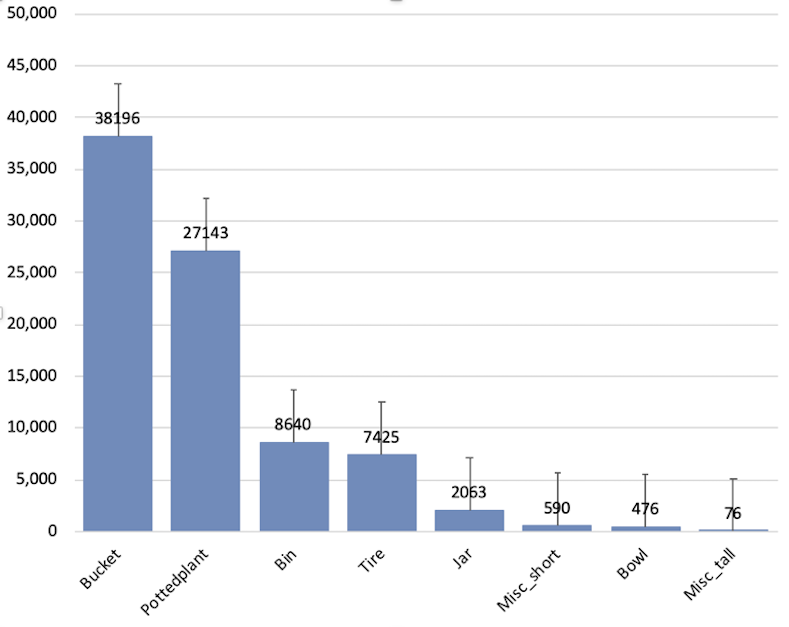

Supplement: S2 Fig — (TIF) [file pntd.0009122.s002.tif]

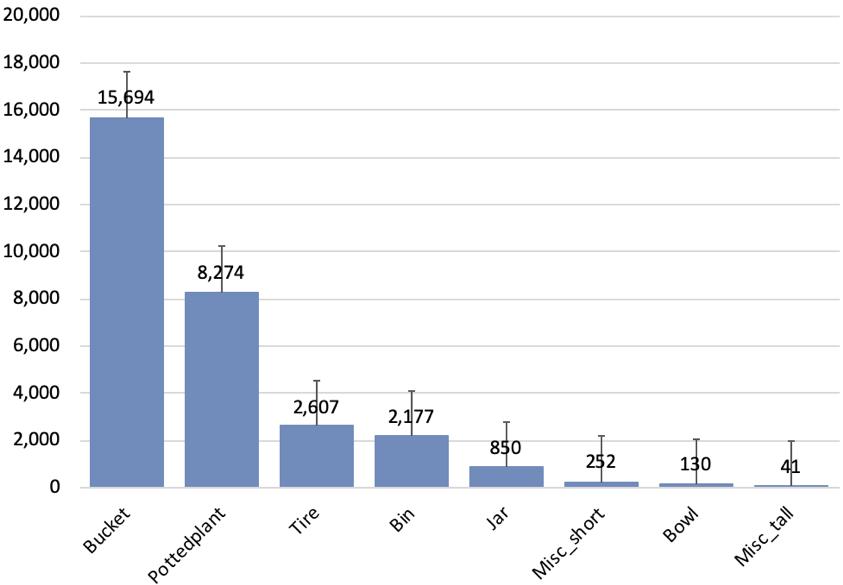

Supplement: S3 Fig — (TIF) [file pntd.0009122.s003.tif]

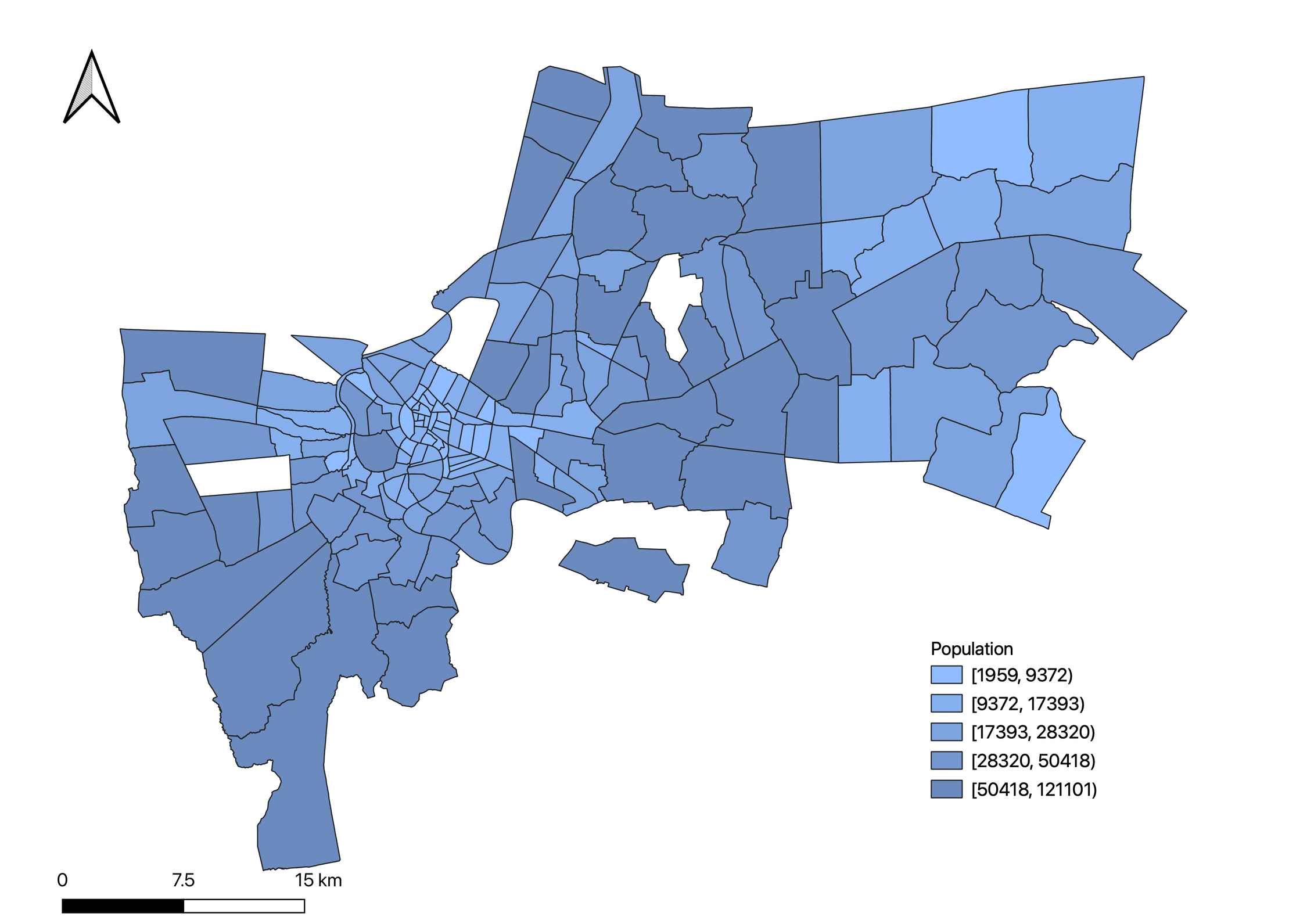

Supplement: S4 Fig — The map in this figure was produced using ArcGIS version 10.4 (Esri, Redlands, CA, USA). Source of shapefile: United Nations Office for the Coordination of Humanitarian Affairs https://data.humdata.org/dataset/thailand-administrative-boundaries. (TIF) [file pntd.0009122.s004.tif]

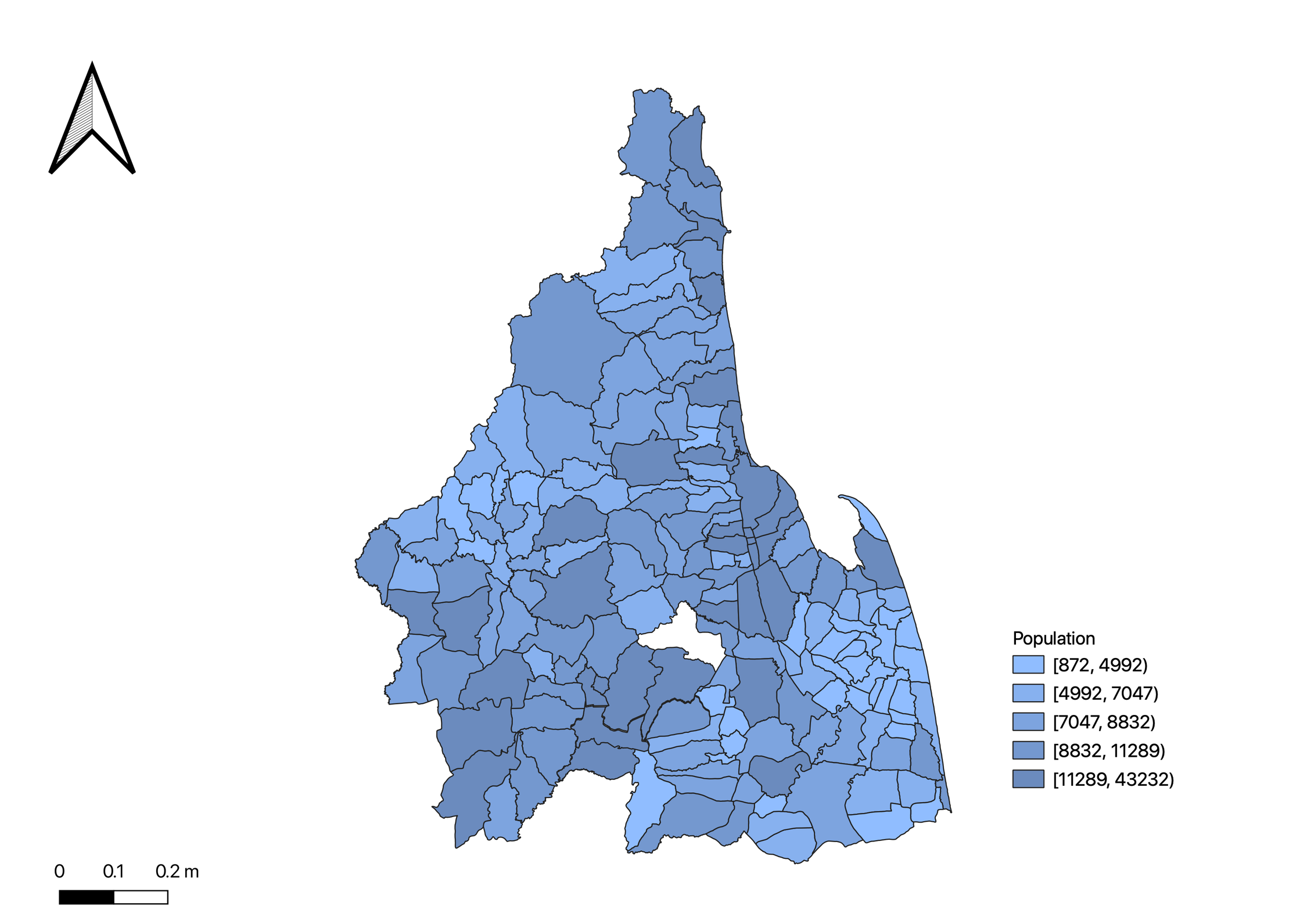

Supplement: S5 Fig — The map in this figure was produced using ArcGIS version 10.4 (Esri, Redlands, CA, USA). Source of shapefile: United Nations Office for the Coordination of Humanitarian Affairs https://data.humdata.org/dataset/thailand-administrative-boundaries. (TIF) [file pntd.0009122.s005.tif]

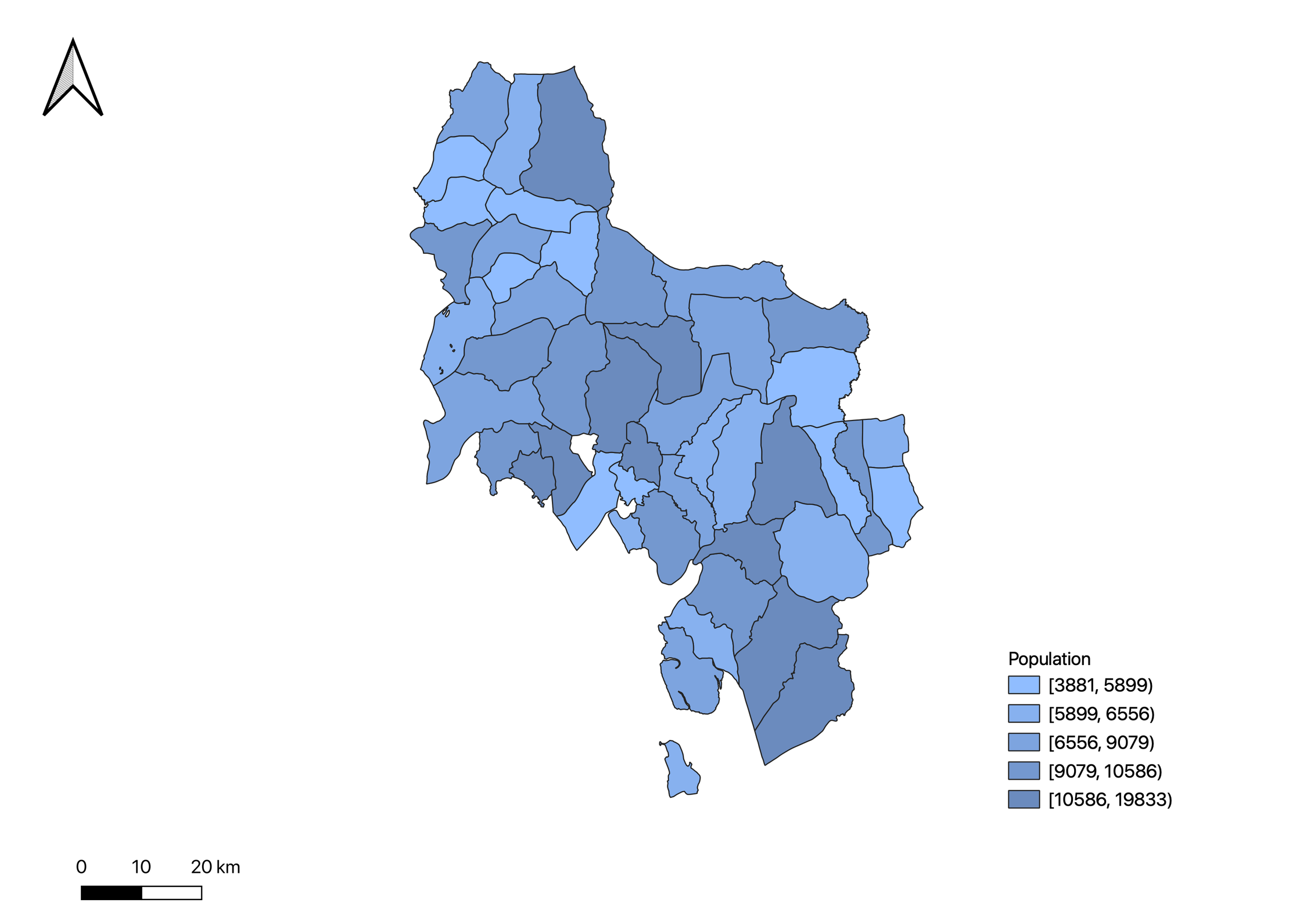

Supplement: S6 Fig — The map in this figure was produced using ArcGIS version 10.4 (Esri, Redlands, CA, USA). Source of shapefile: United Nations Office for the Coordination of Humanitarian Affairs https://data.humdata.org/dataset/thailand-administrative-boundaries. (TIF) [file pntd.0009122.s006.tif]

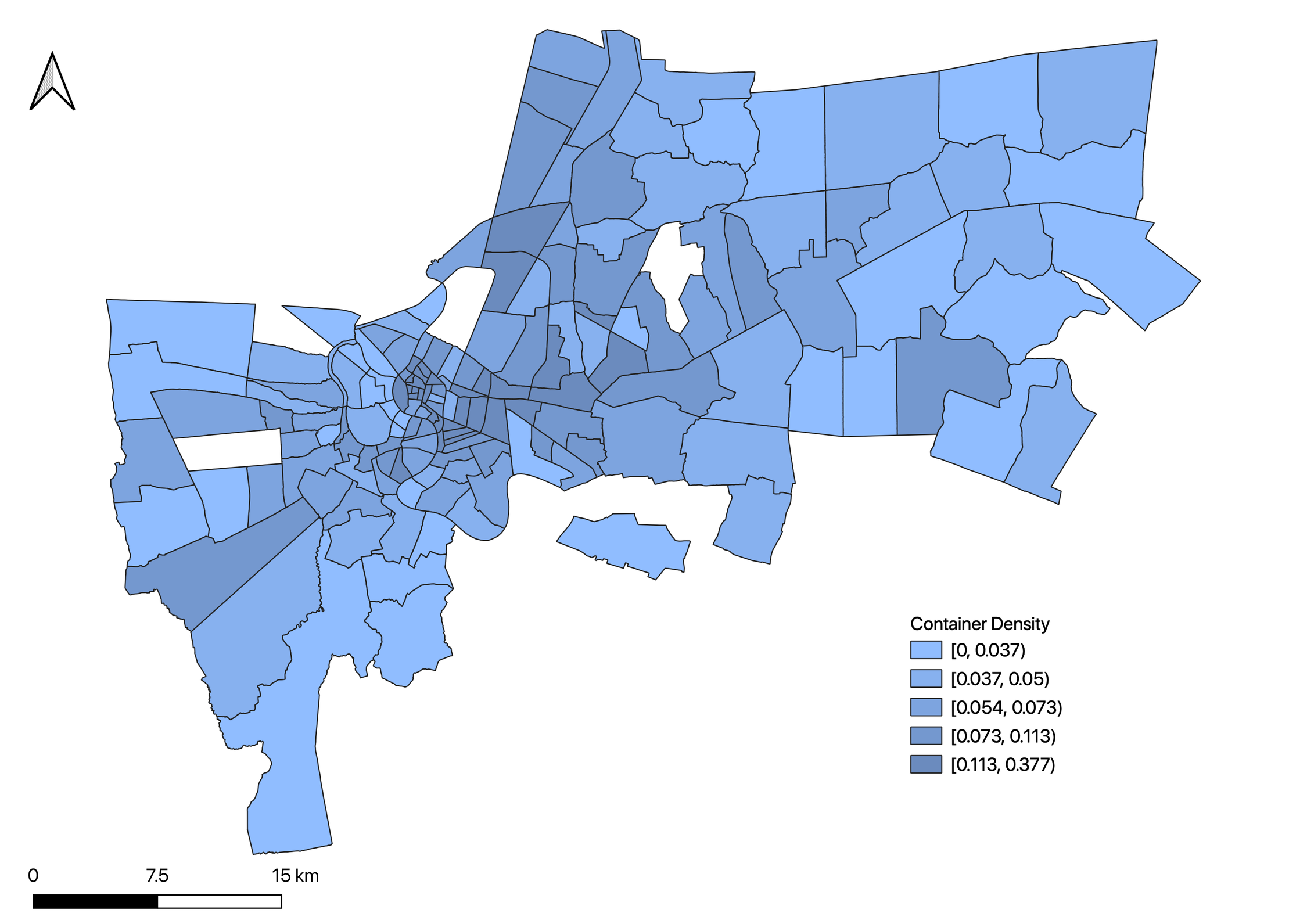

Supplement: S7 Fig — The map in this figure was produced using ArcGIS version 10.4 (Esri, Redlands, CA, USA). Source of shapefile: United Nations Office for the Coordination of Humanitarian Affairs https://data.humdata.org/dataset/thailand-administrative-boundaries. (TIF) [file pntd.0009122.s007.tif]

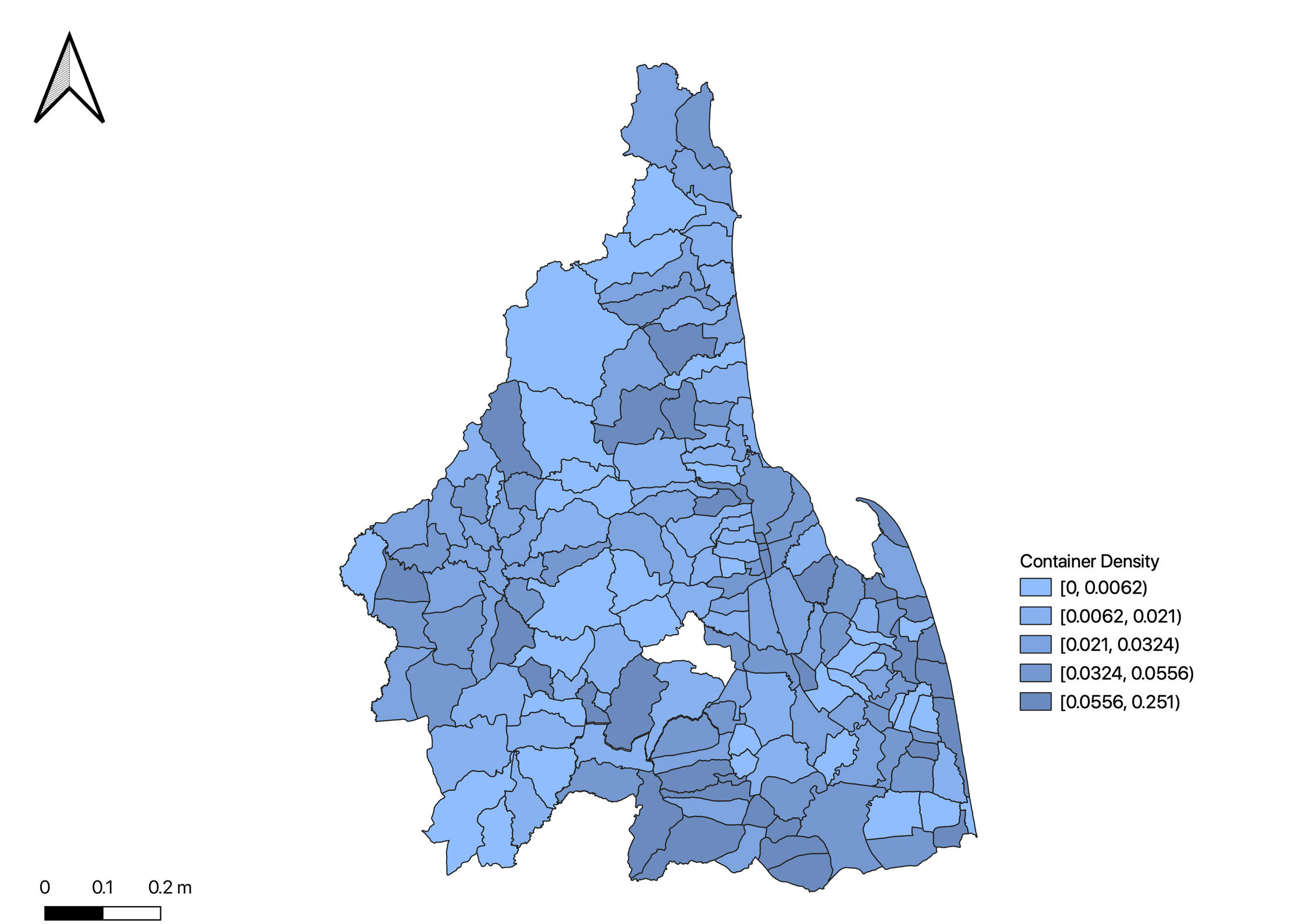

Supplement: S8 Fig — The map in this figure was produced using ArcGIS version 10.4 (Esri, Redlands, CA, USA). Source of shapefile: United Nations Office for the Coordination of Humanitarian Affairs https://data.humdata.org/dataset/thailand-administrative-boundaries. (TIF) [file pntd.0009122.s008.tif]

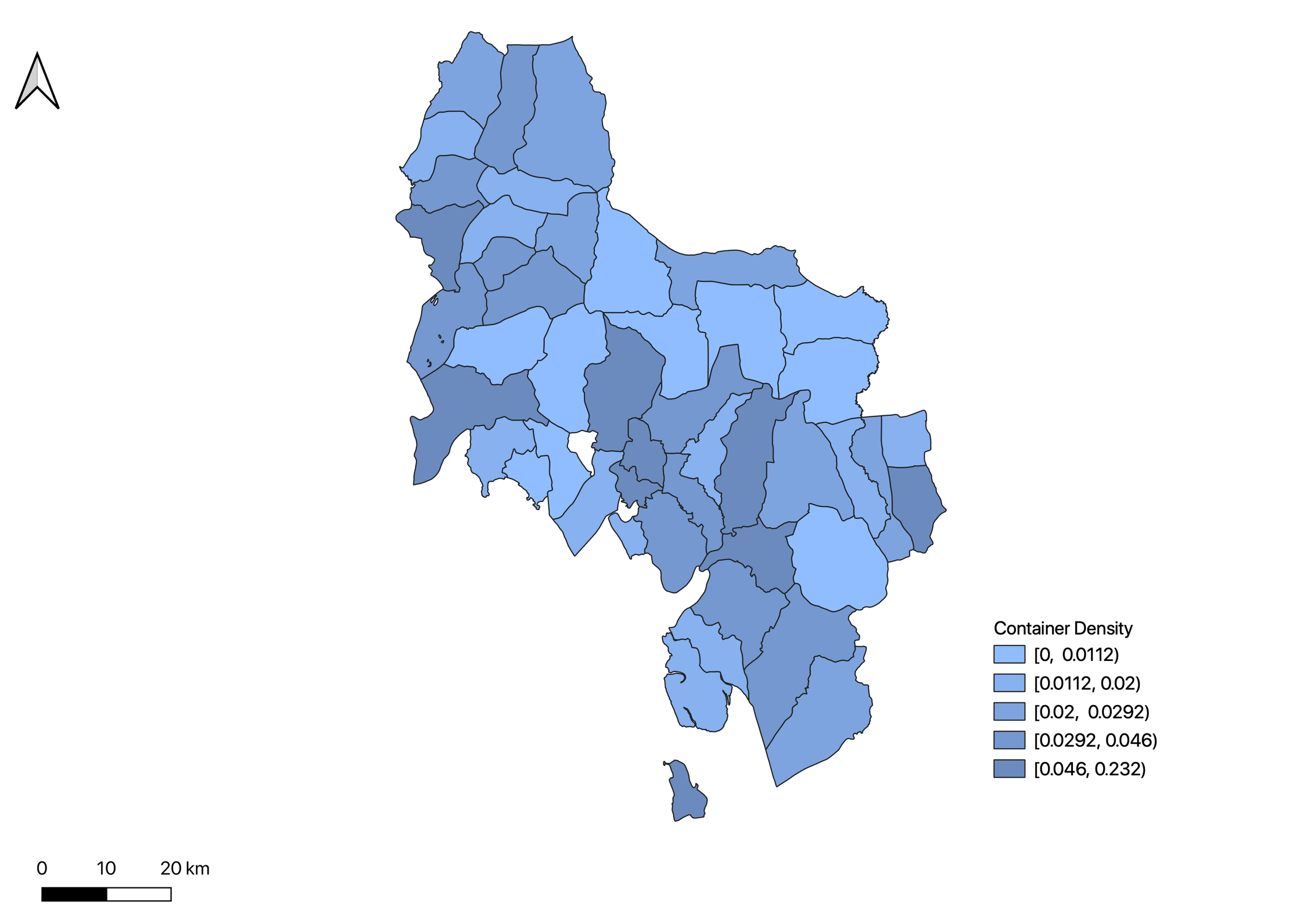

Supplement: S9 Fig — The map in this figure was produced using ArcGIS version 10.4 (Esri, Redlands, CA, USA). Source of shapefile: United Nations Office for the Coordination of Humanitarian Affairs https://data.humdata.org/dataset/thailand-administrative-boundaries. (TIF) [file pntd.0009122.s009.tif]

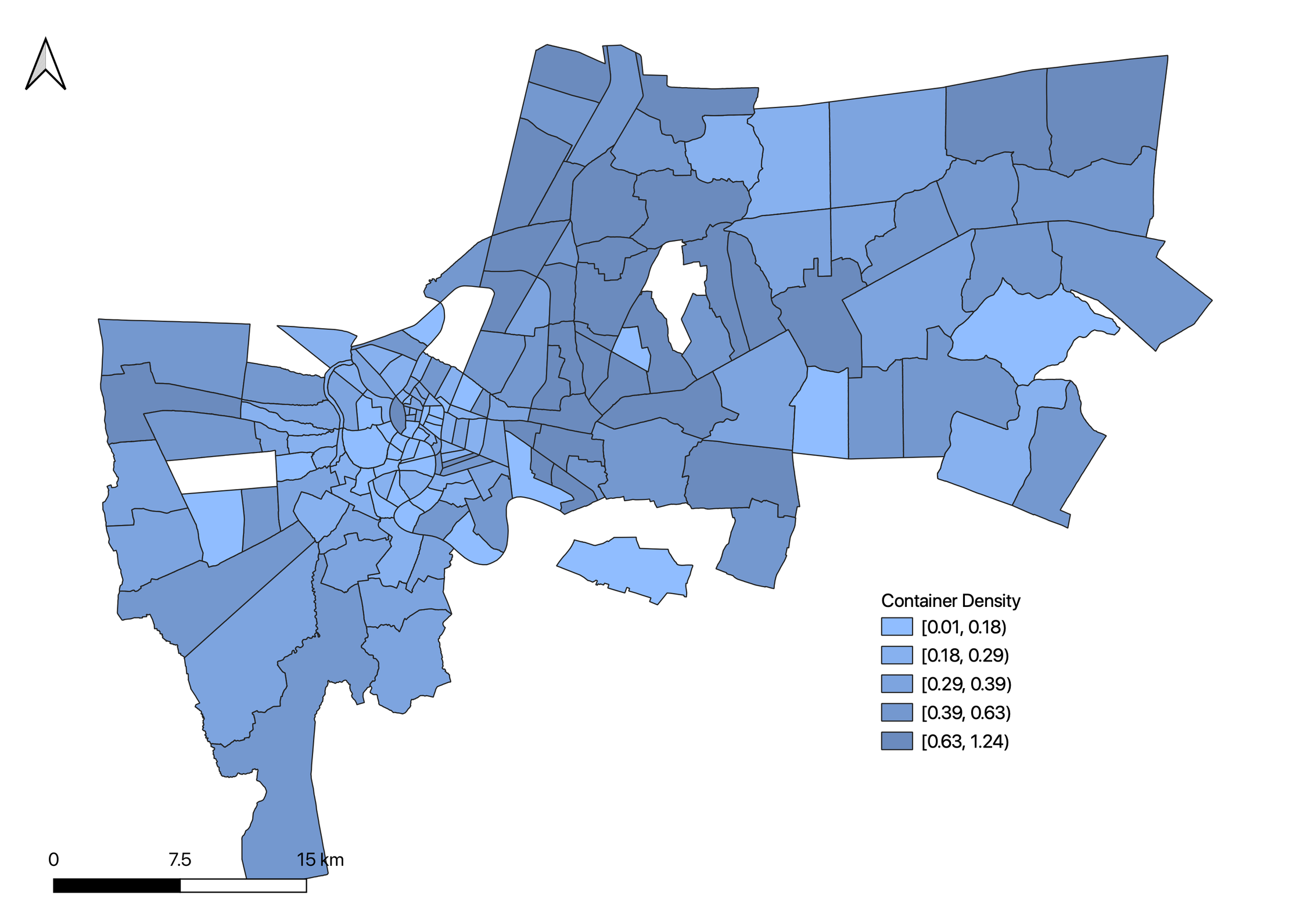

Supplement: S10 Fig — The map in this figure was produced using ArcGIS version 10.4 (Esri, Redlands, CA, USA). Source of shapefile: United Nations Office for the Coordination of Humanitarian Affairs https://data.humdata.org/dataset/thailand-administrative-boundaries. (TIF) [file pntd.0009122.s010.tif]

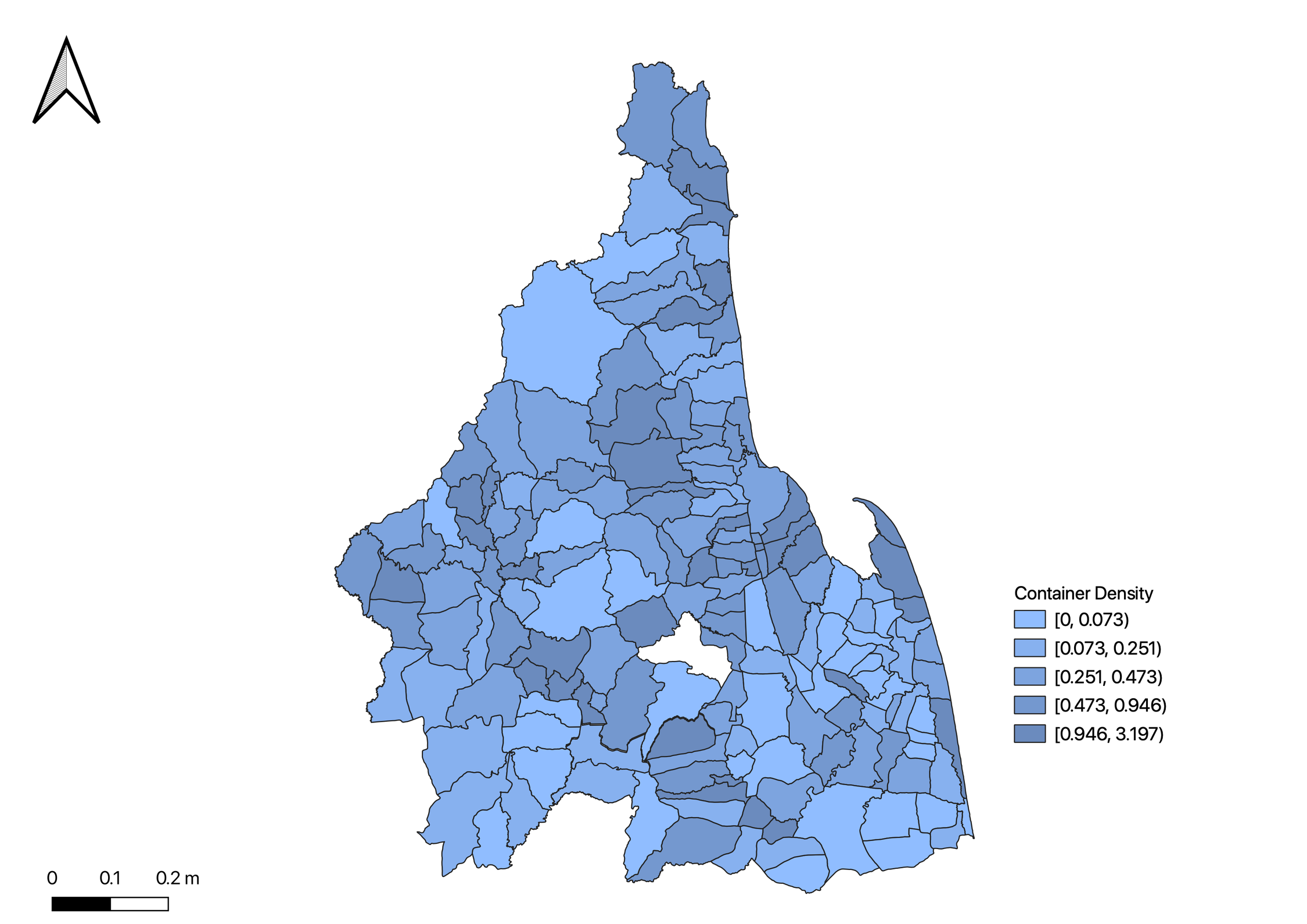

Supplement: S11 Fig — The map in this figure was produced using ArcGIS version 10.4 (Esri, Redlands, CA, USA). Source of shapefile: United Nations Office for the Coordination of Humanitarian Affairs https://data.humdata.org/dataset/thailand-administrative-boundaries. (TIF) [file pntd.0009122.s011.tif]

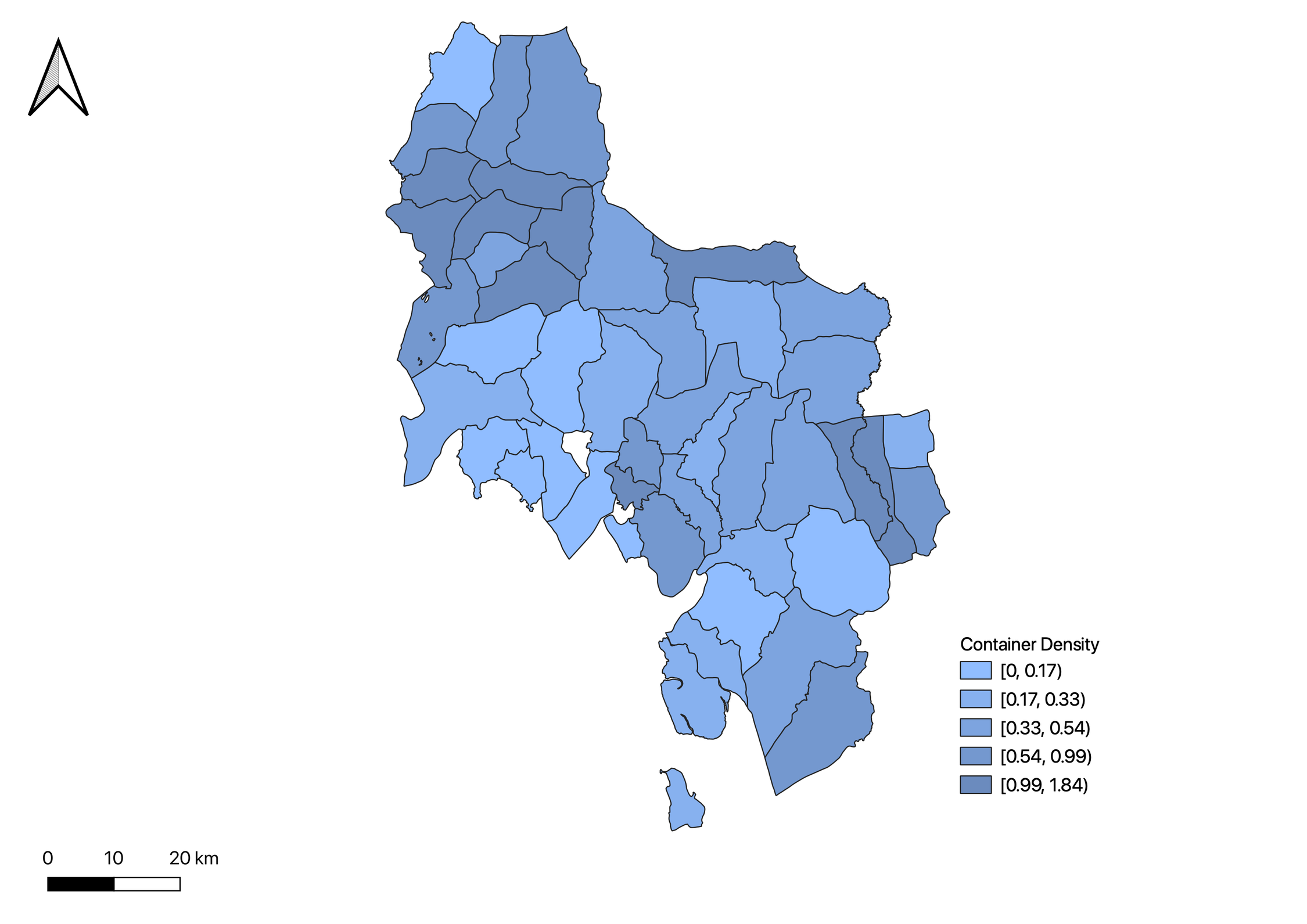

Supplement: S12 Fig — The map in this figure was produced using ArcGIS version 10.4 (Esri, Redlands, CA, USA). Source of shapefile: United Nations Office for the Coordination of Humanitarian Affairs https://data.humdata.org/dataset/thailand-administrative-boundaries. (TIF) [file pntd.0009122.s012.tif]

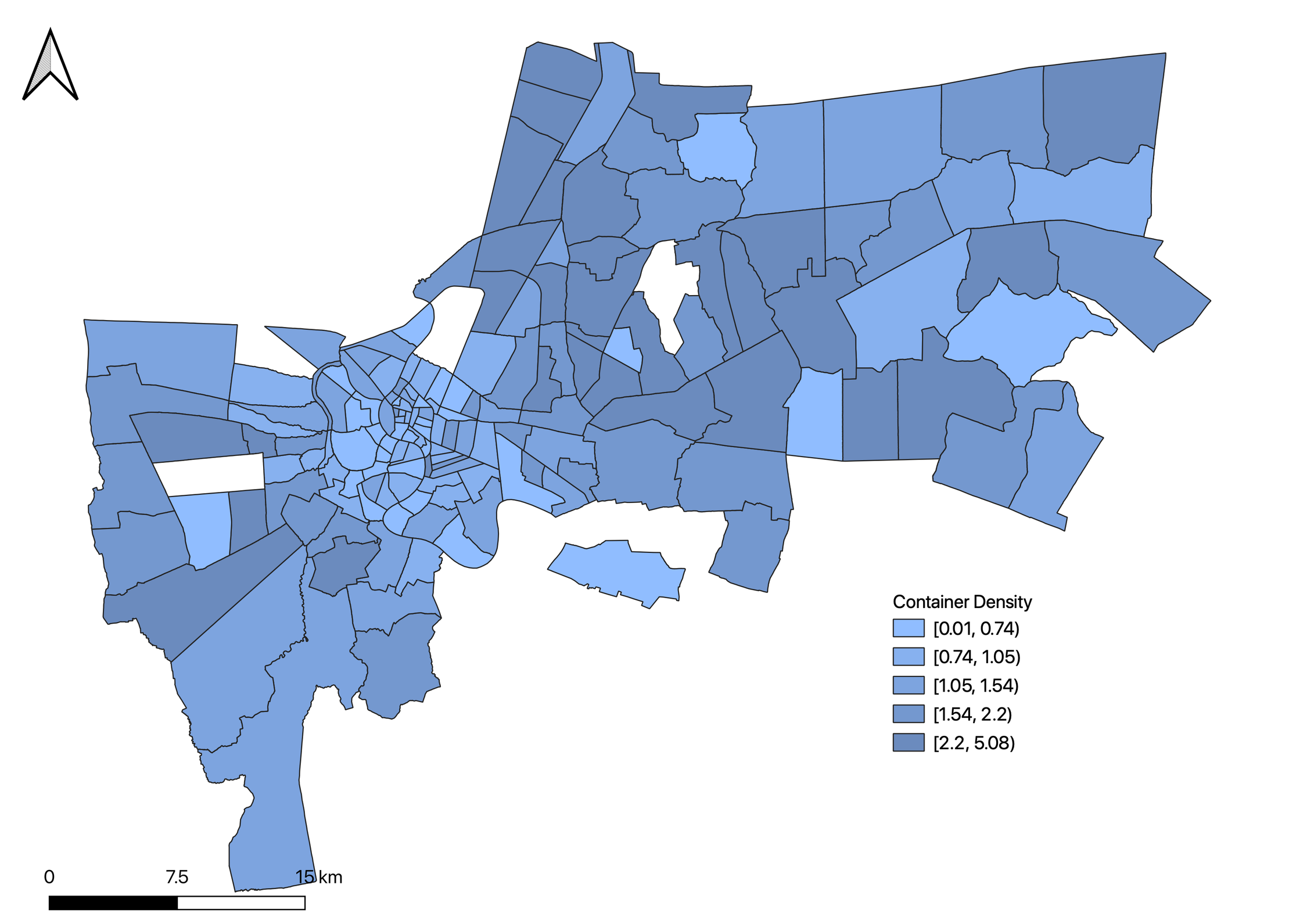

Supplement: S13 Fig — The map in this figure was produced using ArcGIS version 10.4 (Esri, Redlands, CA, USA). Source of shapefile: United Nations Office for the Coordination of Humanitarian Affairs https://data.humdata.org/dataset/thailand-administrative-boundaries. (TIF) [file pntd.0009122.s013.tif]

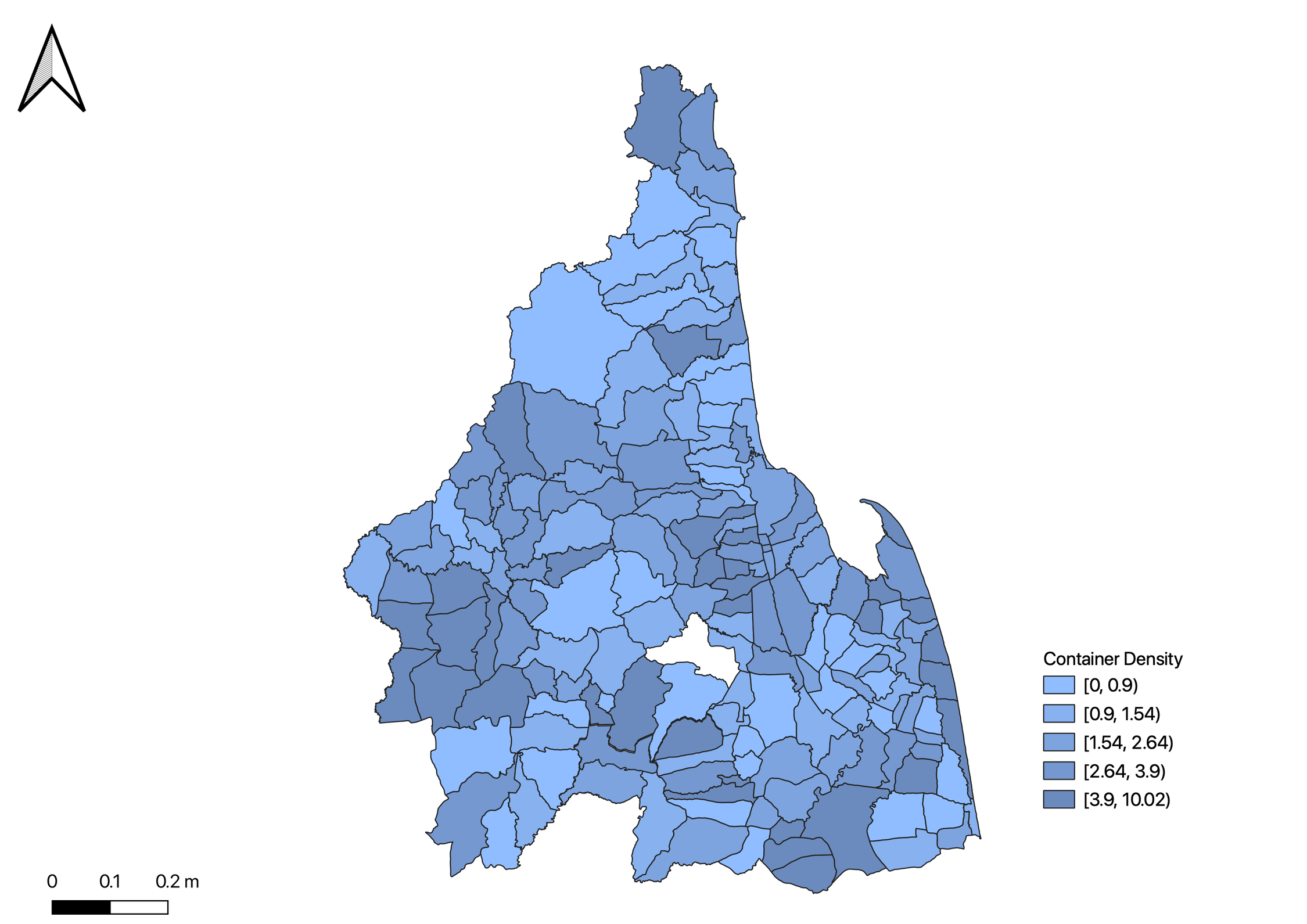

Supplement: S14 Fig — The map in this figure was produced using ArcGIS version 10.4 (Esri, Redlands, CA, USA). Source of shapefile: United Nations Office for the Coordination of Humanitarian Affairs https://data.humdata.org/dataset/thailand-administrative-boundaries. (TIF) [file pntd.0009122.s014.tif]

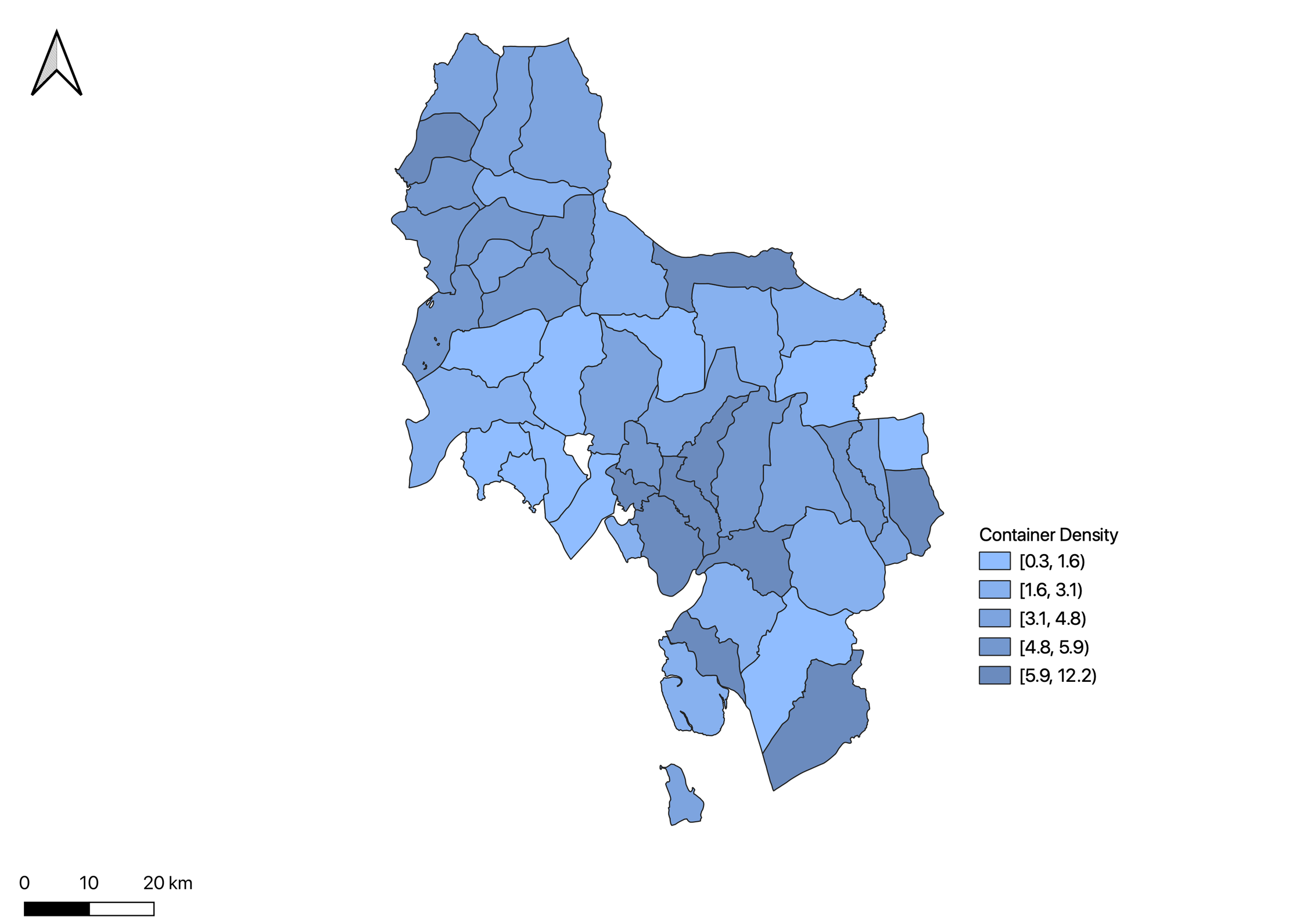

Supplement: S15 Fig — The map in this figure was produced using ArcGIS version 10.4 (Esri, Redlands, CA, USA). Source of shapefile: United Nations Office for the Coordination of Humanitarian Affairs https://data.humdata.org/dataset/thailand-administrative-boundaries. (TIF) [file pntd.0009122.s015.tif]

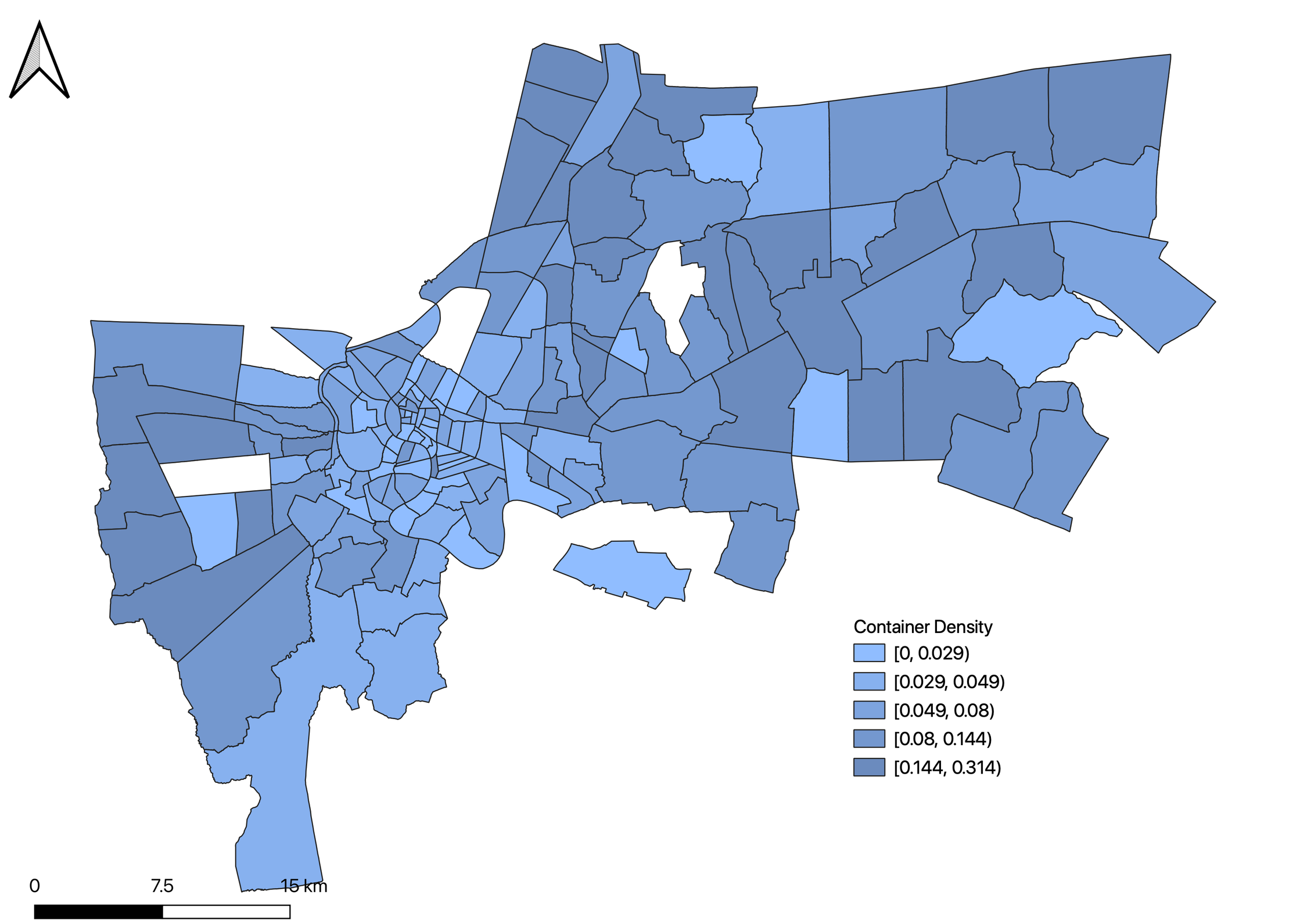

Supplement: S16 Fig — The map in this figure was produced using ArcGIS version 10.4 (Esri, Redlands, CA, USA). Source of shapefile: United Nations Office for the Coordination of Humanitarian Affairs https://data.humdata.org/dataset/thailand-administrative-boundaries. (TIF) [file pntd.0009122.s016.tif]

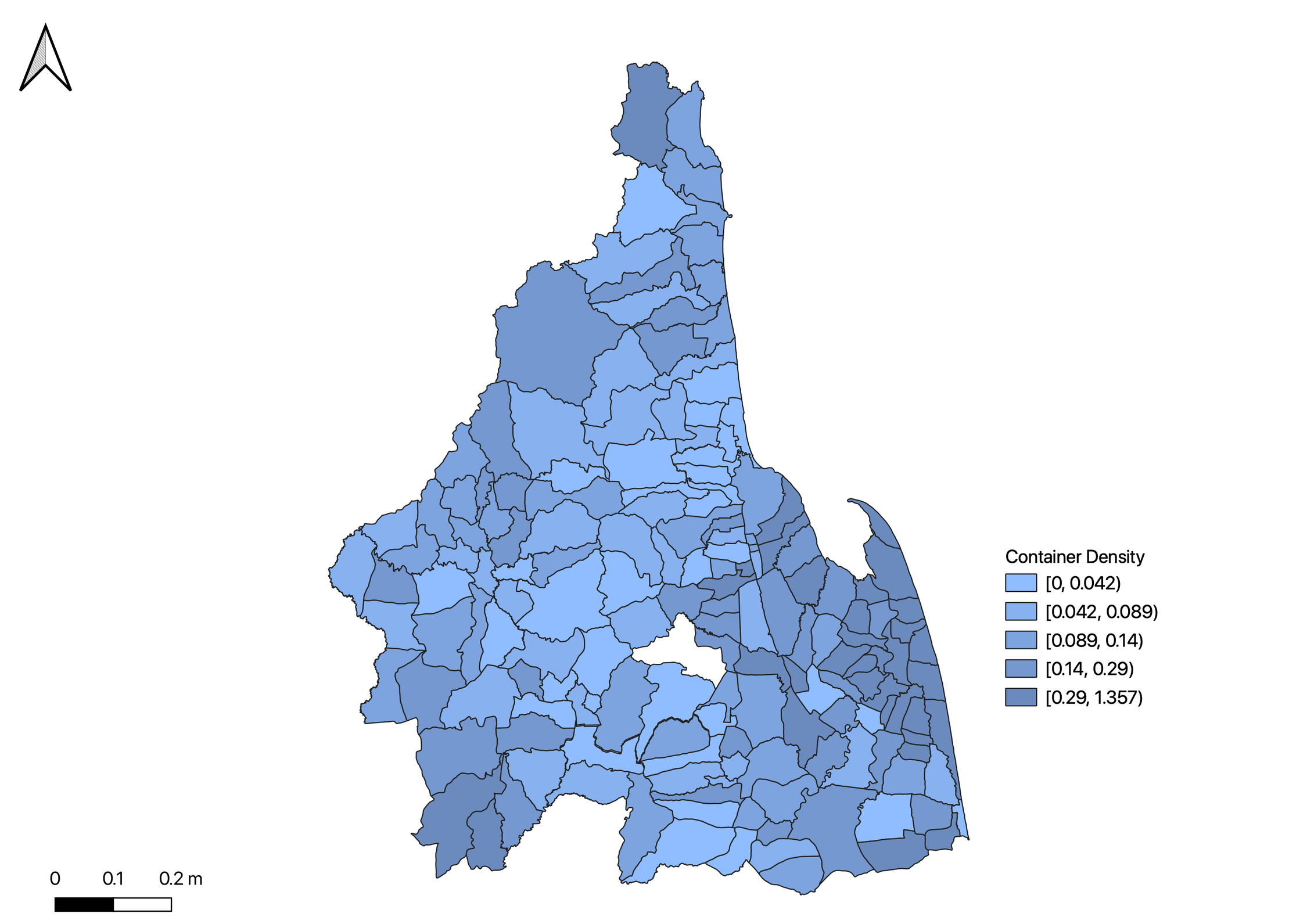

Supplement: S17 Fig — The map in this figure was produced using ArcGIS version 10.4 (Esri, Redlands, CA, USA). Source of shapefile: United Nations Office for the Coordination of Humanitarian Affairs https://data.humdata.org/dataset/thailand-administrative-boundaries. (TIF) [file pntd.0009122.s017.tif]

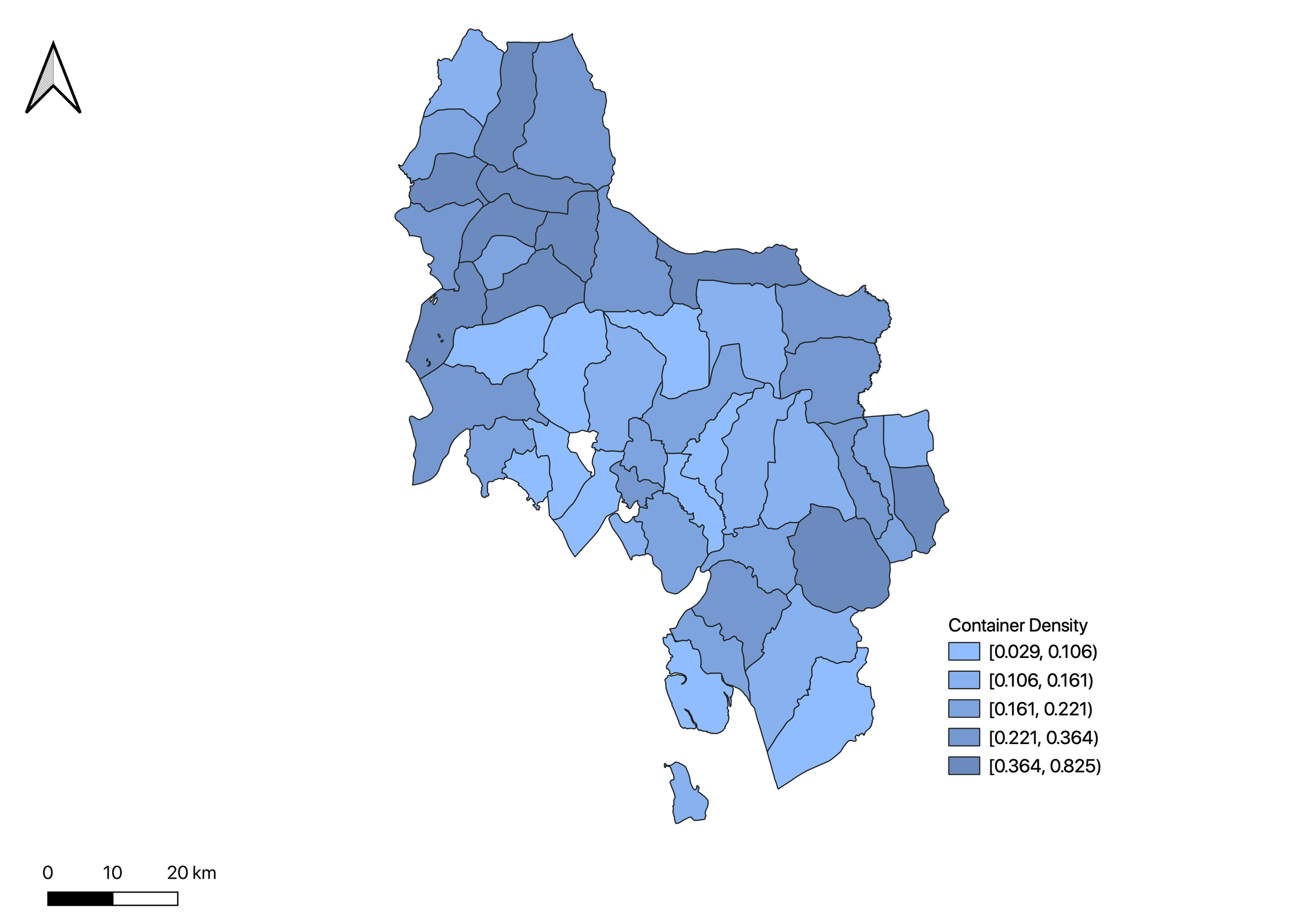

Supplement: S18 Fig — The map in this figure was produced using ArcGIS version 10.4 (Esri, Redlands, CA, USA). Source of shapefile: United Nations Office for the Coordination of Humanitarian Affairs https://data.humdata.org/dataset/thailand-administrative-boundaries. (TIF) [file pntd.0009122.s018.tif]

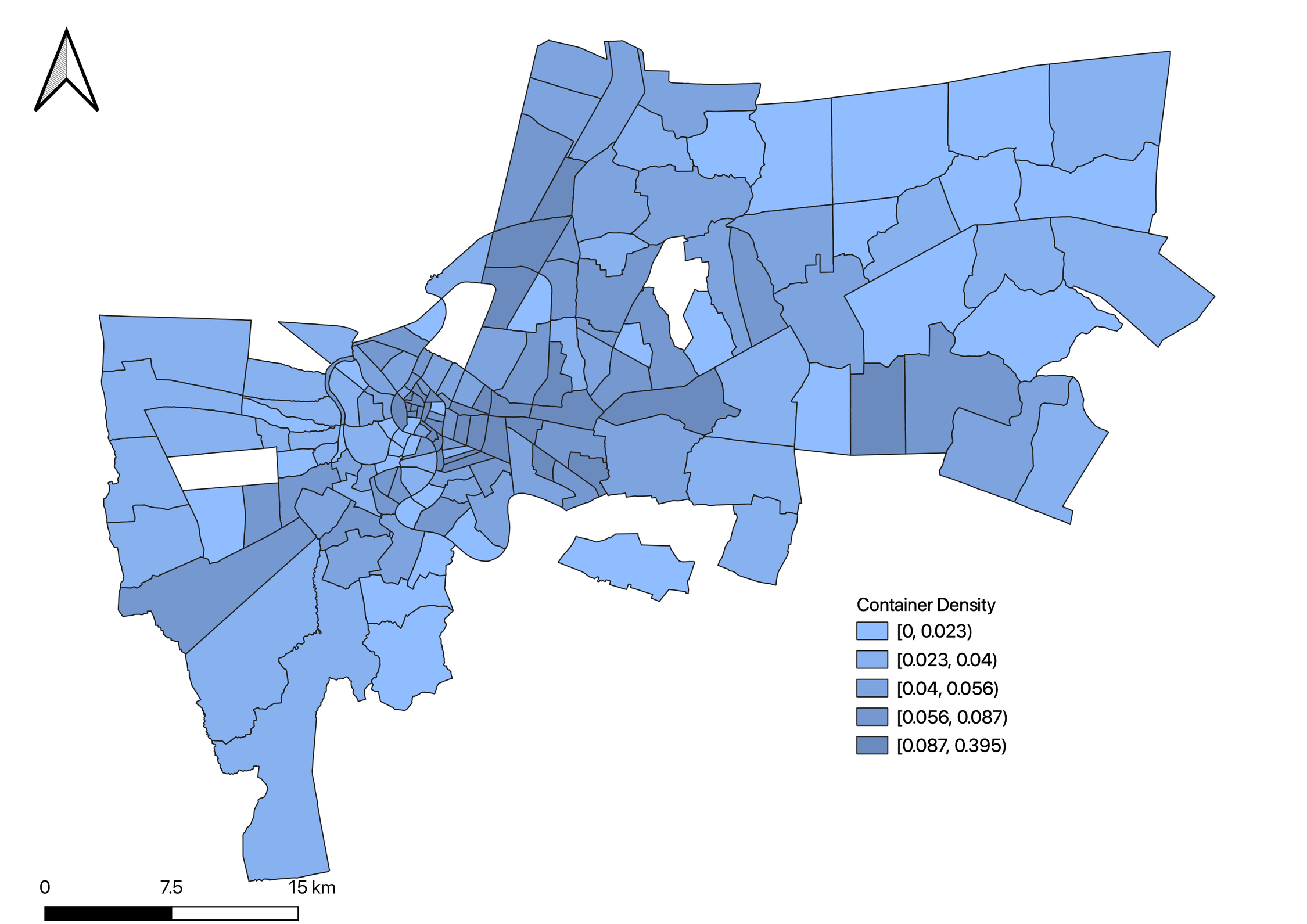

Supplement: S19 Fig — The map in this figure was produced using ArcGIS version 10.4 (Esri, Redlands, CA, USA). Source of shapefile: United Nations Office for the Coordination of Humanitarian Affairs https://data.humdata.org/dataset/thailand-administrative-boundaries. (TIF) [file pntd.0009122.s019.tif]

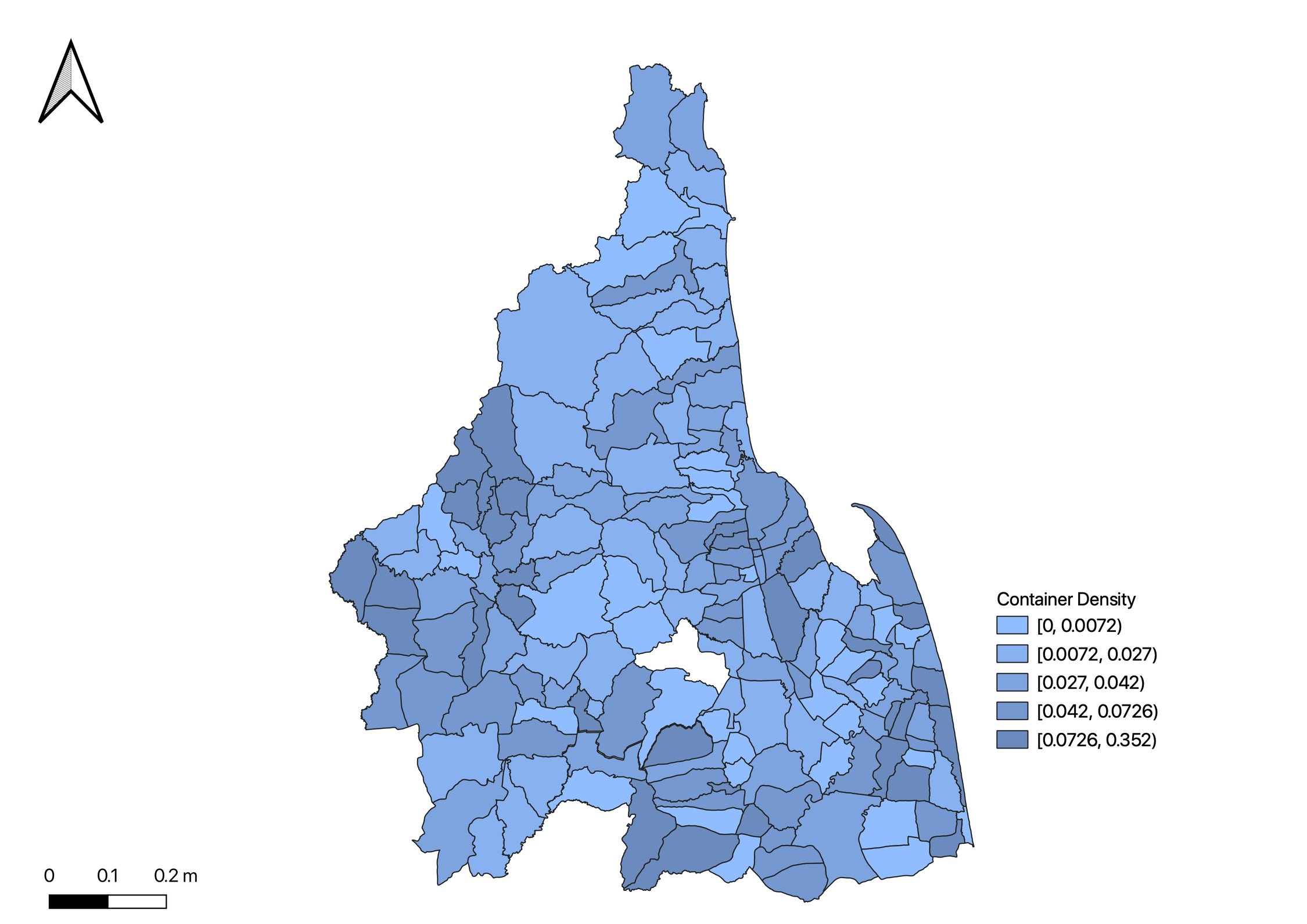

Supplement: S20 Fig — The map in this figure was produced using ArcGIS version 10.4 (Esri, Redlands, CA, USA). Source of shapefile: United Nations Office for the Coordination of Humanitarian Affairs https://data.humdata.org/dataset/thailand-administrative-boundaries. (TIF) [file pntd.0009122.s020.tif]

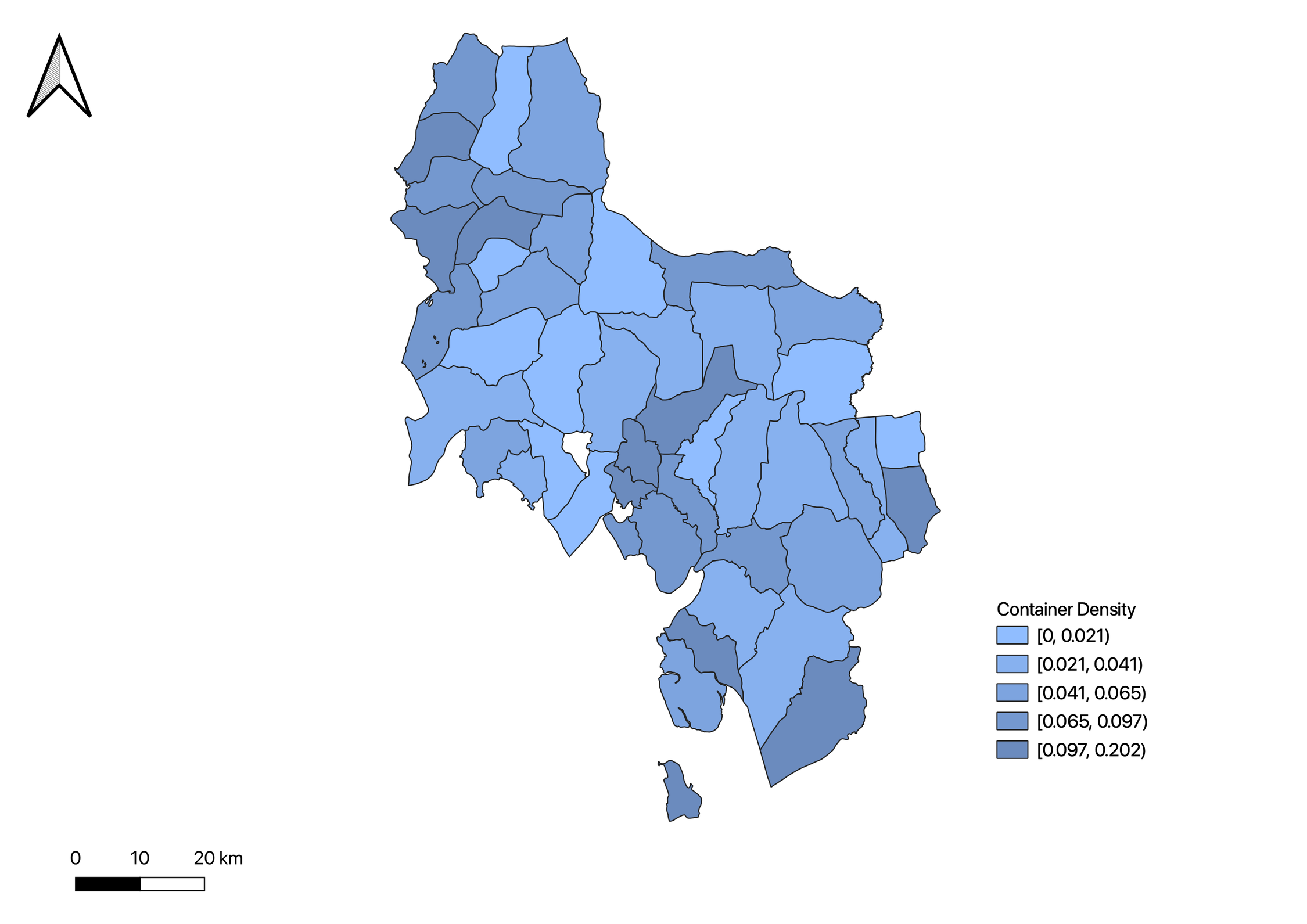

Supplement: S21 Fig — The map in this figure was produced using ArcGIS version 10.4 (Esri, Redlands, CA, USA). Source of shapefile: United Nations Office for the Coordination of Humanitarian Affairs https://data.humdata.org/dataset/thailand-administrative-boundaries. (TIF) [file pntd.0009122.s021.tif]

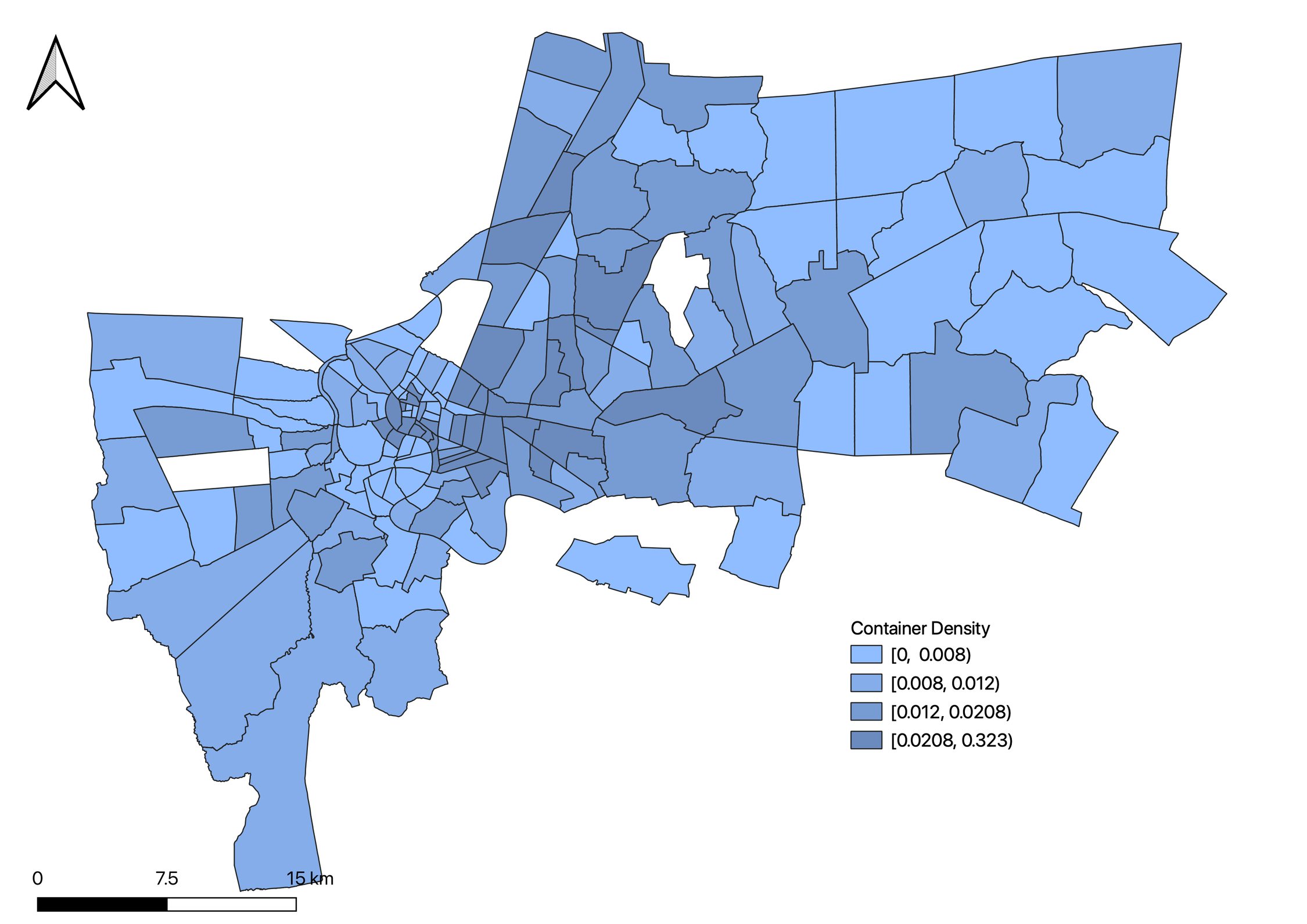

Supplement: S22 Fig — The map in this figure was produced using ArcGIS version 10.4 (Esri, Redlands, CA, USA). Source of shapefile: United Nations Office for the Coordination of Humanitarian Affairs https://data.humdata.org/dataset/thailand-administrative-boundaries. (TIF) [file pntd.0009122.s022.tif]

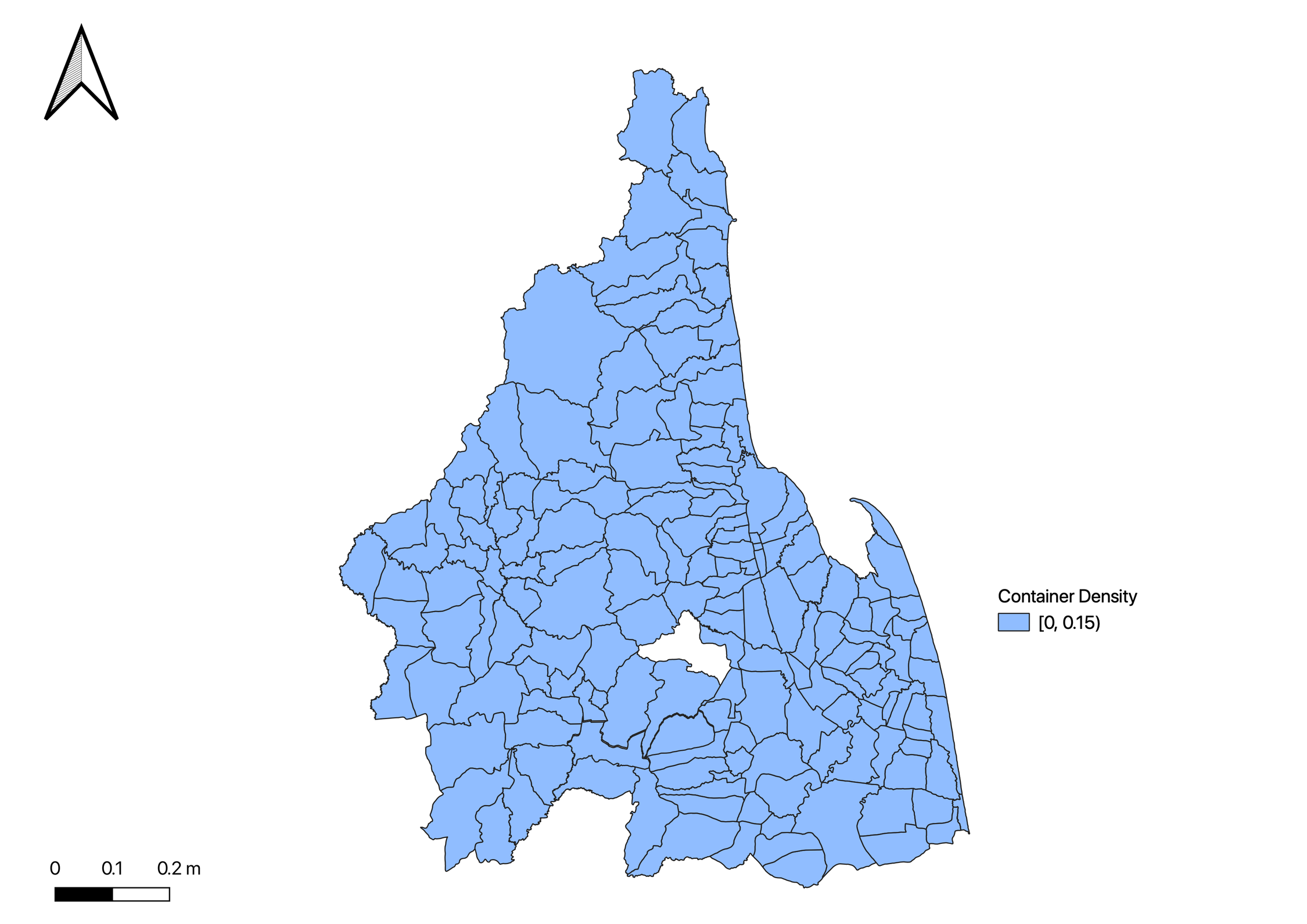

Supplement: S23 Fig — The map in this figure was produced using ArcGIS version 10.4 (Esri, Redlands, CA, USA). Source of shapefile: United Nations Office for the Coordination of Humanitarian Affairs https://data.humdata.org/dataset/thailand-administrative-boundaries. (TIF) [file pntd.0009122.s023.tif]

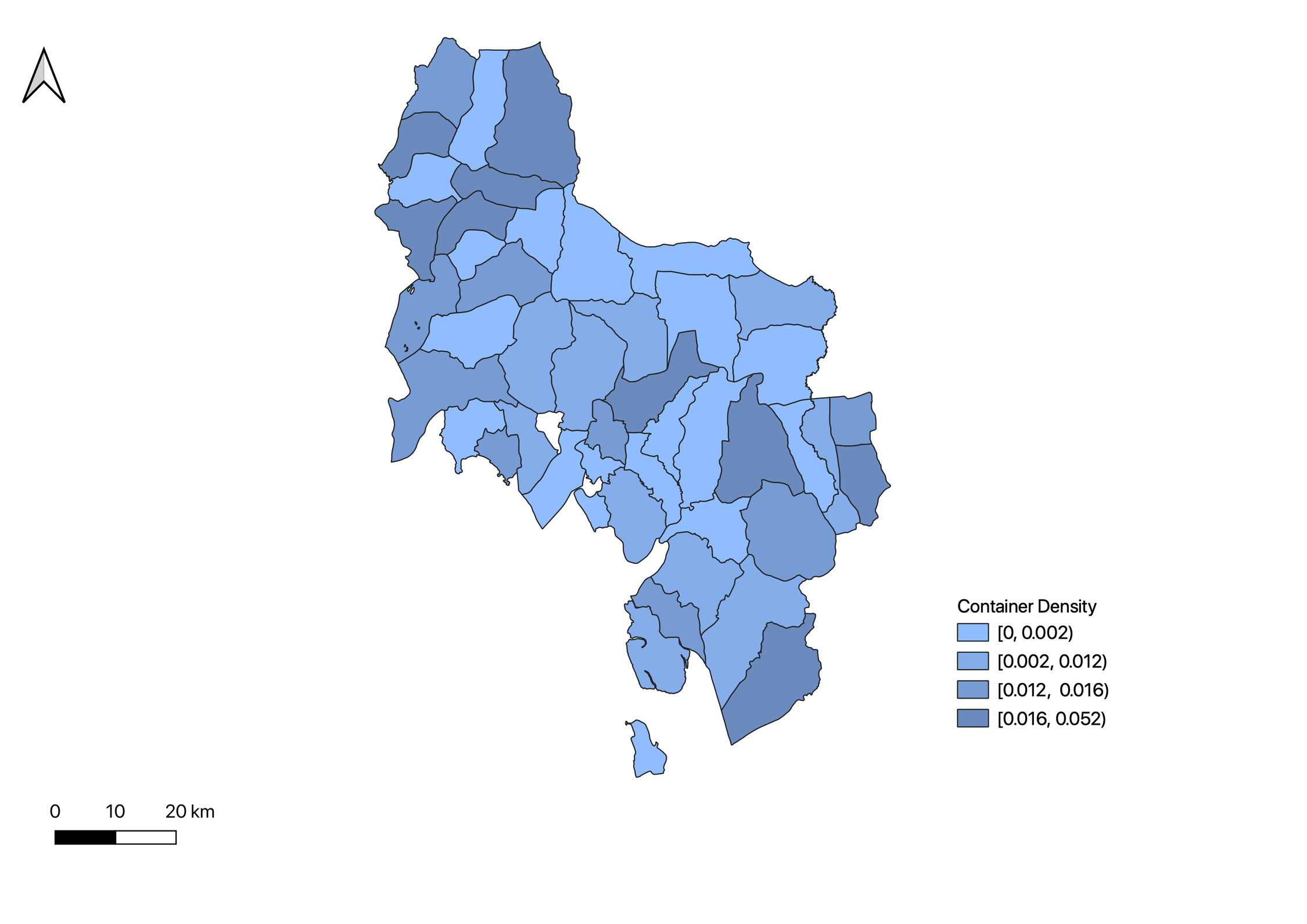

Supplement: S24 Fig — The map in this figure was produced using ArcGIS version 10.4 (Esri, Redlands, CA, USA). Source of shapefile: United Nations Office for the Coordination of Humanitarian Affairs https://data.humdata.org/dataset/thailand-administrative-boundaries. (TIF) [file pntd.0009122.s024.tif]

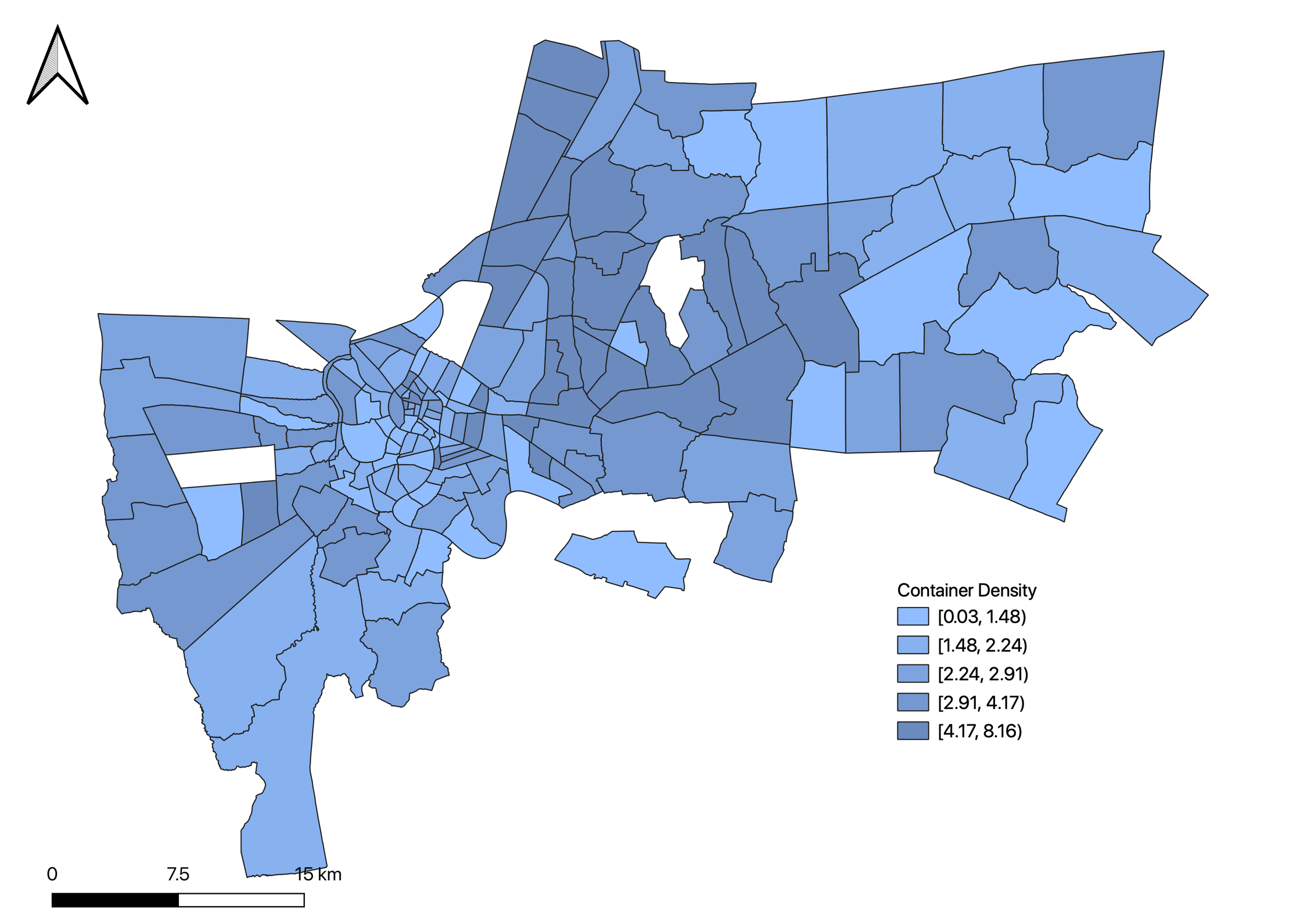

Supplement: S25 Fig — The map in this figure was produced using ArcGIS version 10.4 (Esri, Redlands, CA, USA). Source of shapefile: United Nations Office for the Coordination of Humanitarian Affairs https://data.humdata.org/dataset/thailand-administrative-boundaries. (TIF) [file pntd.0009122.s025.tif]

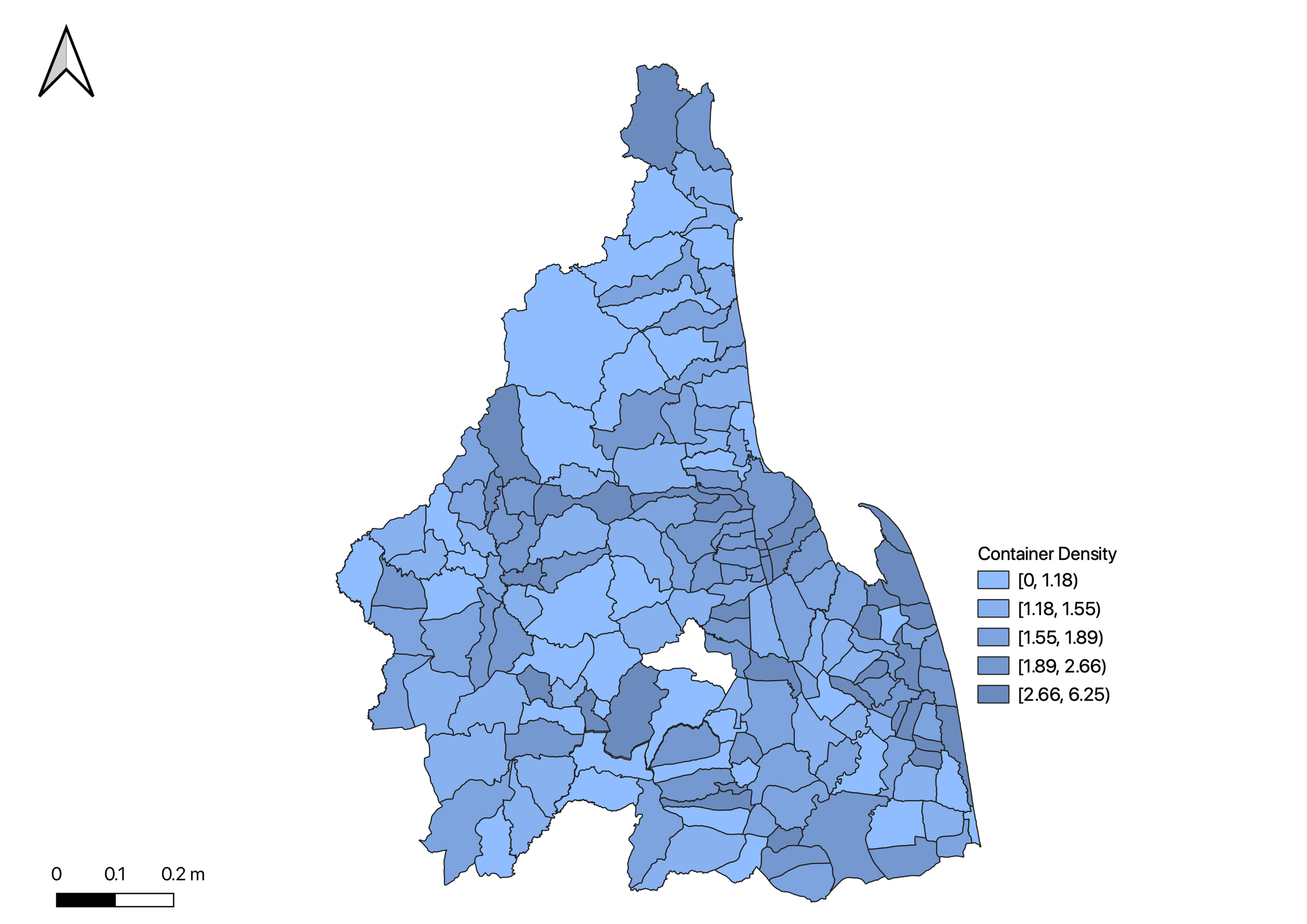

Supplement: S26 Fig — The map in this figure was produced using ArcGIS version 10.4 (Esri, Redlands, CA, USA). Source of shapefile: United Nations Office for the Coordination of Humanitarian Affairs https://data.humdata.org/dataset/thailand-administrative-boundaries. (TIF) [file pntd.0009122.s026.tif]

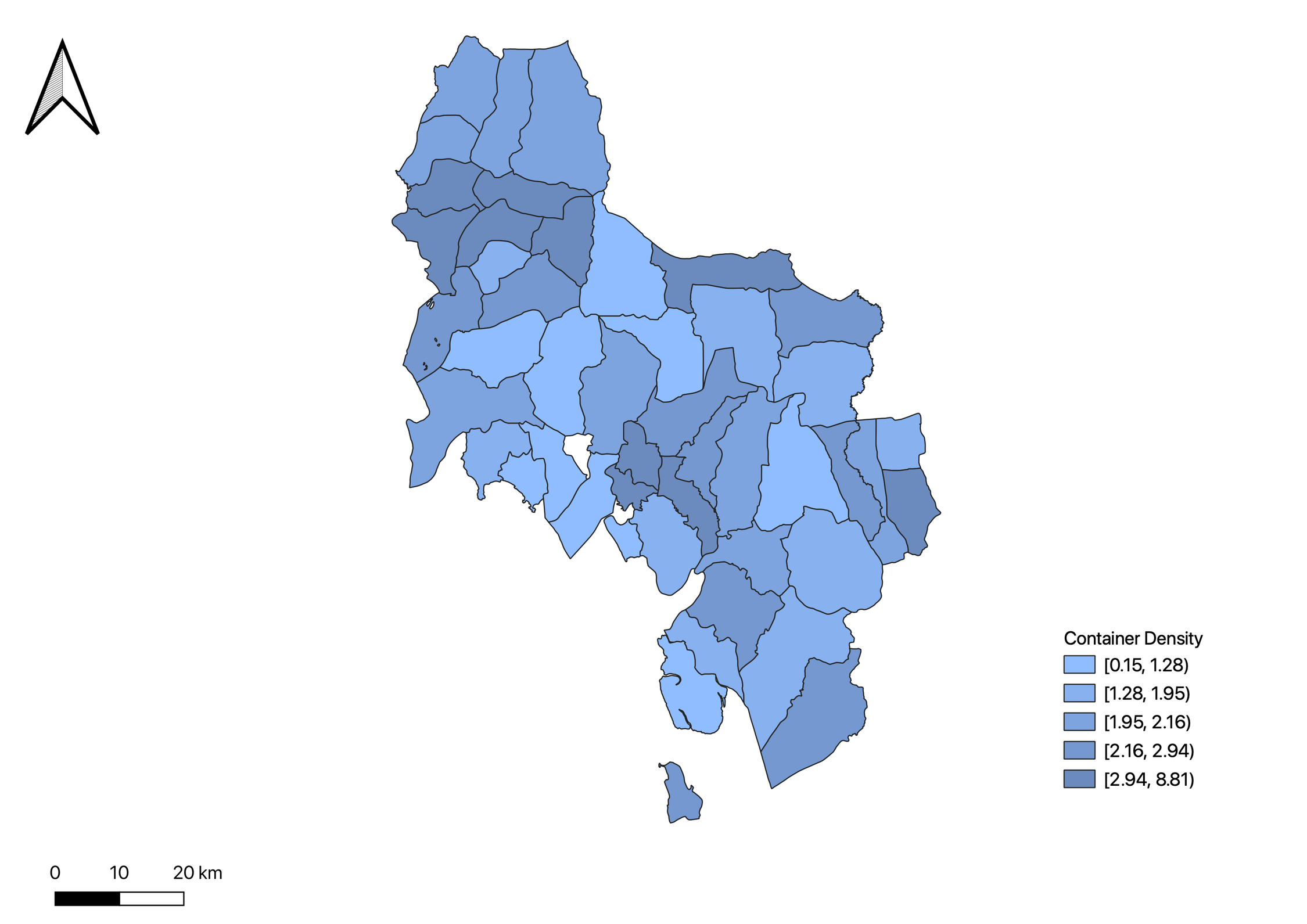

Supplement: S27 Fig — The map in this figure was produced using ArcGIS version 10.4 (Esri, Redlands, CA, USA). Source of shapefile: United Nations Office for the Coordination of Humanitarian Affairs https://data.humdata.org/dataset/thailand-administrative-boundaries. (TIF) [file pntd.0009122.s027.tif]

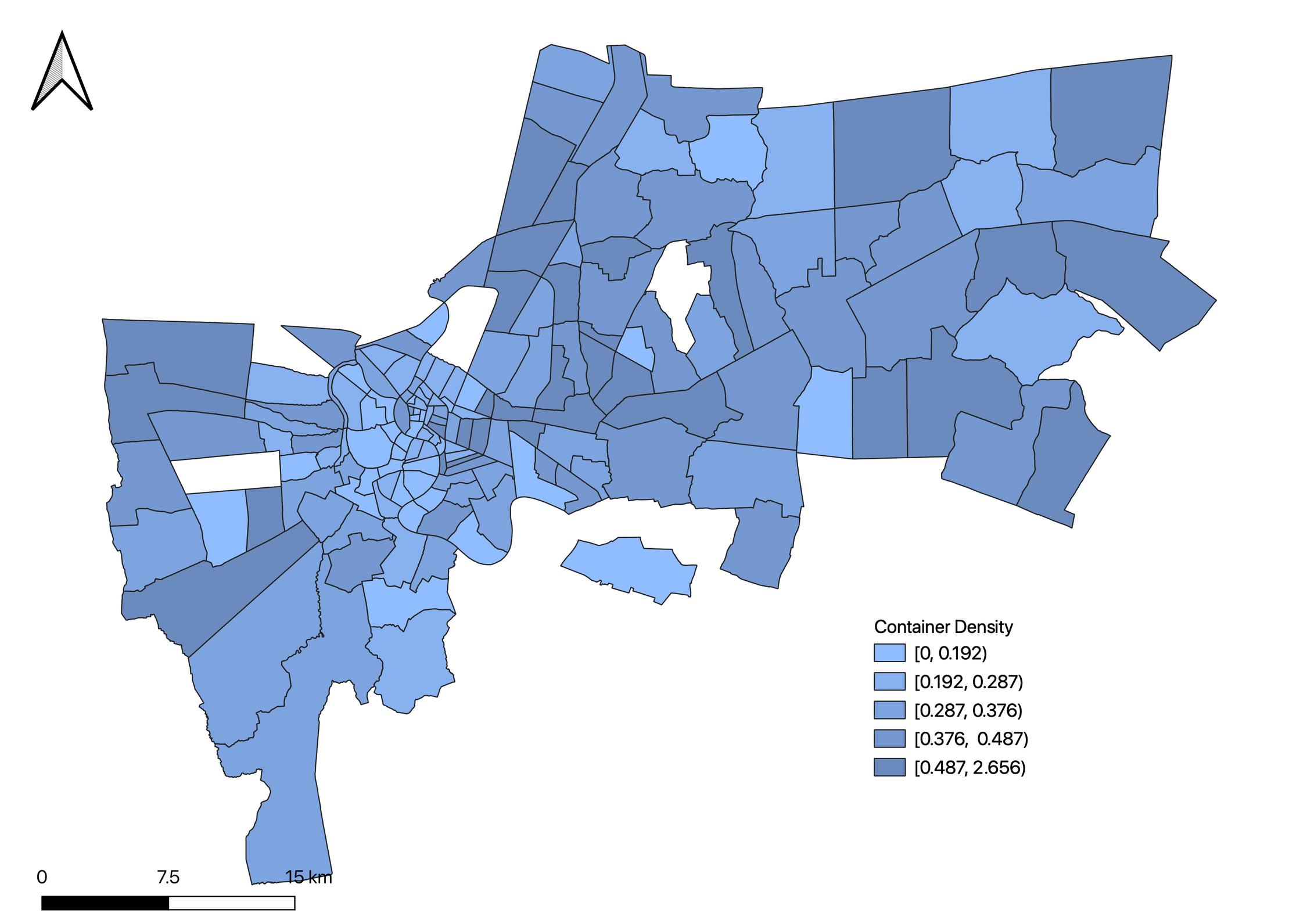

Supplement: S28 Fig — The map in this figure was produced using ArcGIS version 10.4 (Esri, Redlands, CA, USA). Source of shapefile: United Nations Office for the Coordination of Humanitarian Affairs https://data.humdata.org/dataset/thailand-administrative-boundaries. (TIF) [file pntd.0009122.s028.tif]

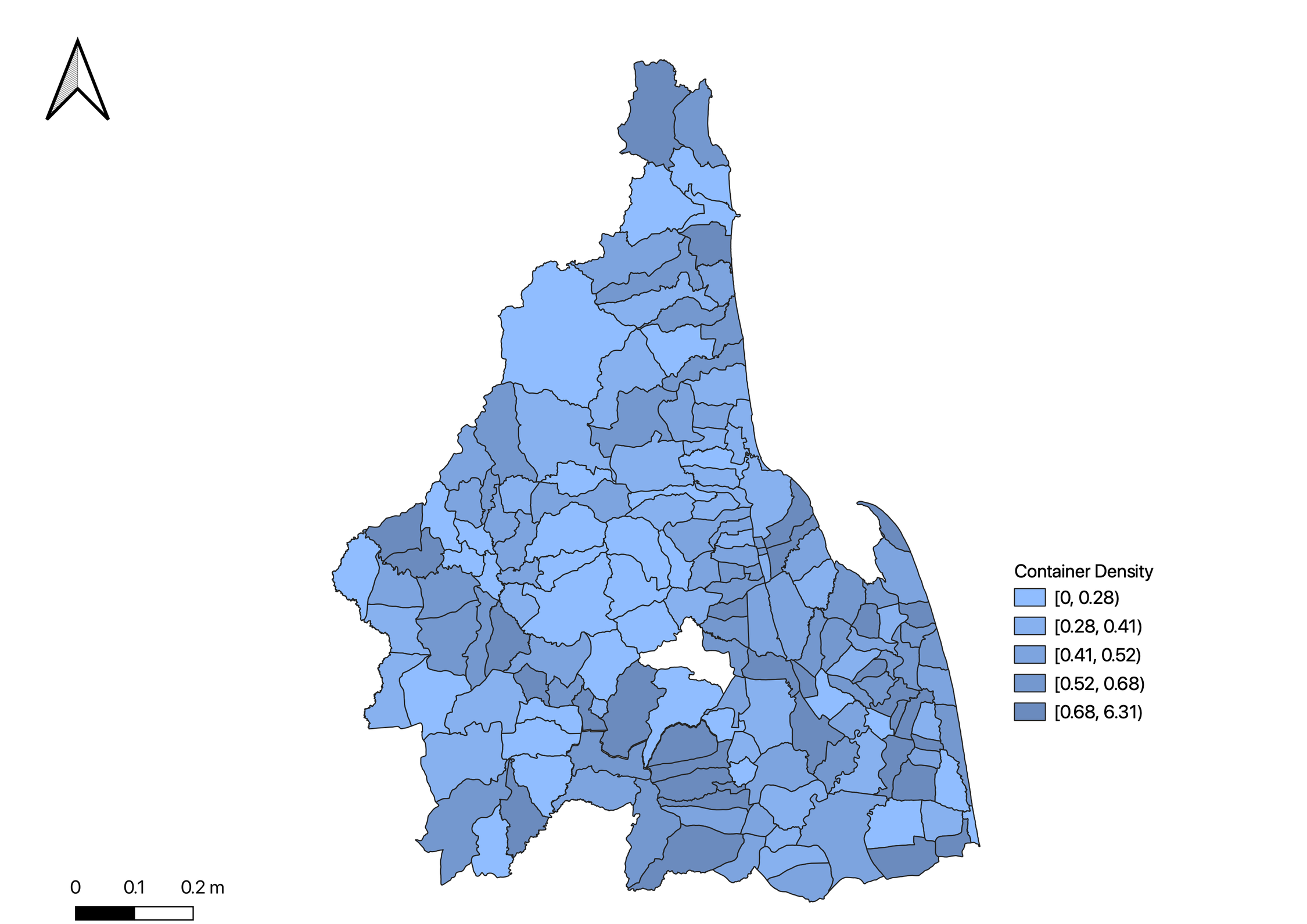

Supplement: S29 Fig — The map in this figure was produced using ArcGIS version 10.4 (Esri, Redlands, CA, USA). Source of shapefile: United Nations Office for the Coordination of Humanitarian Affairs https://data.humdata.org/dataset/thailand-administrative-boundaries. (TIF) [file pntd.0009122.s029.tif]

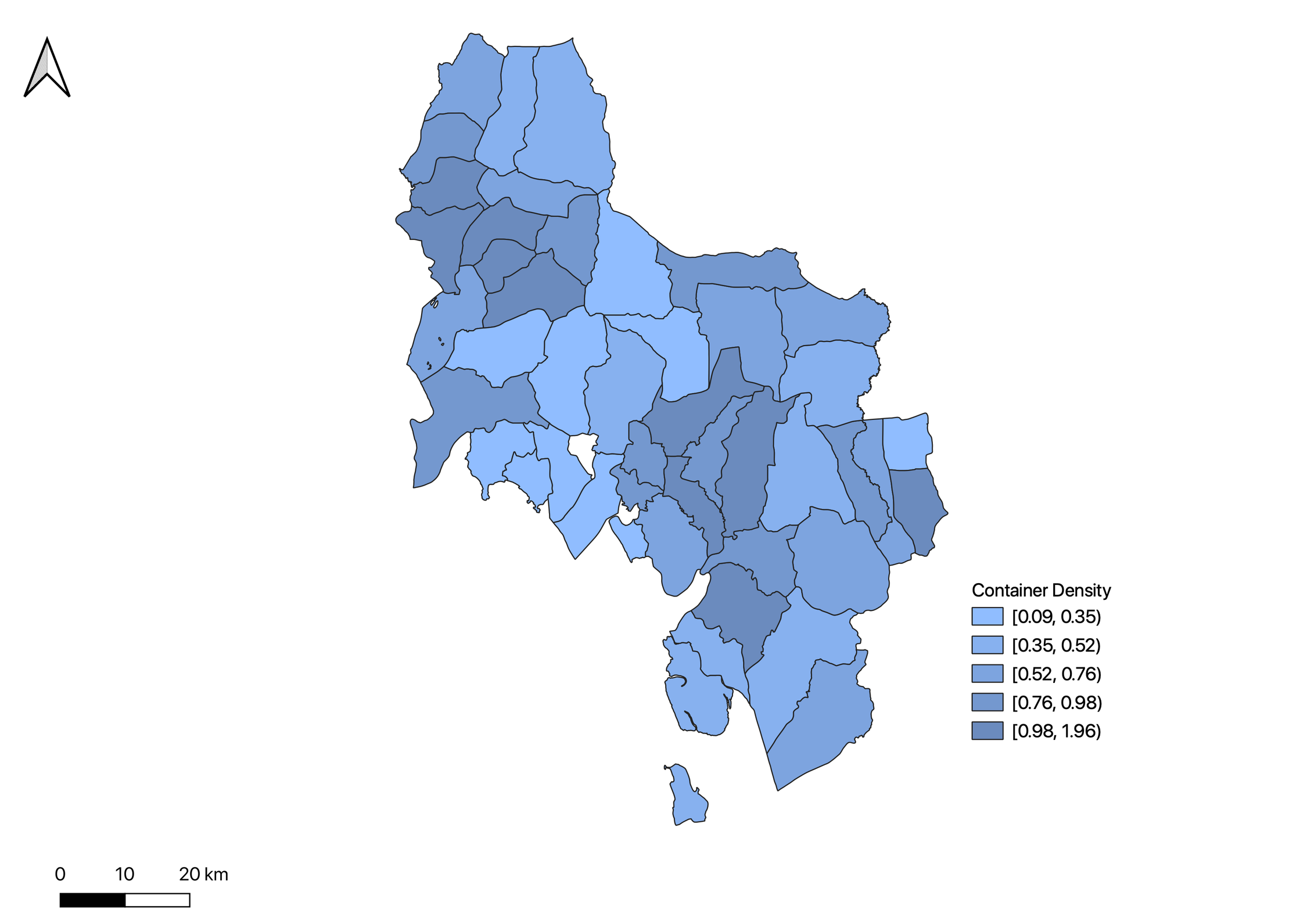

Supplement: S30 Fig — The map in this figure was produced using ArcGIS version 10.4 (Esri, Redlands, CA, USA). Source of shapefile: United Nations Office for the Coordination of Humanitarian Affairs https://data.humdata.org/dataset/thailand-administrative-boundaries. (TIF) [file pntd.0009122.s030.tif]

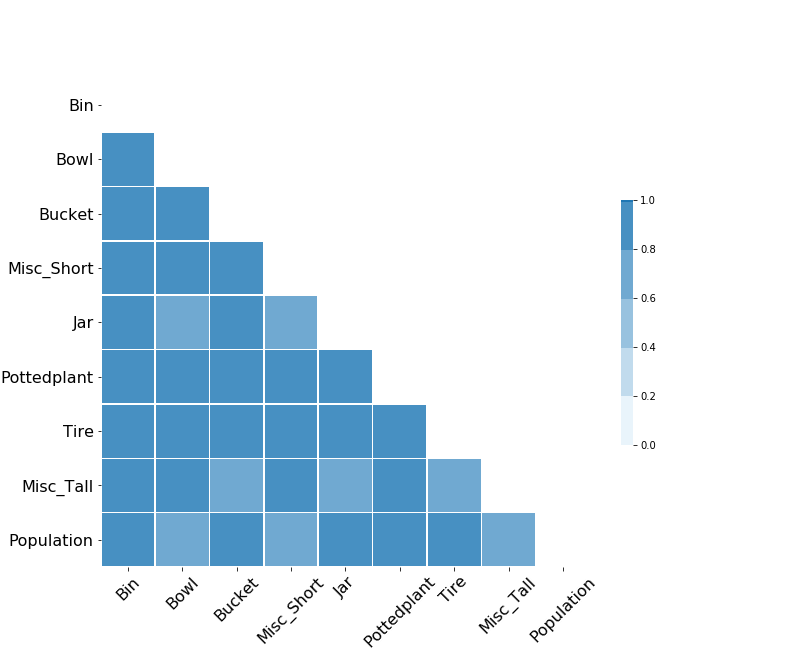

Supplement: S31 Fig — (TIF) [file pntd.0009122.s031.tif]

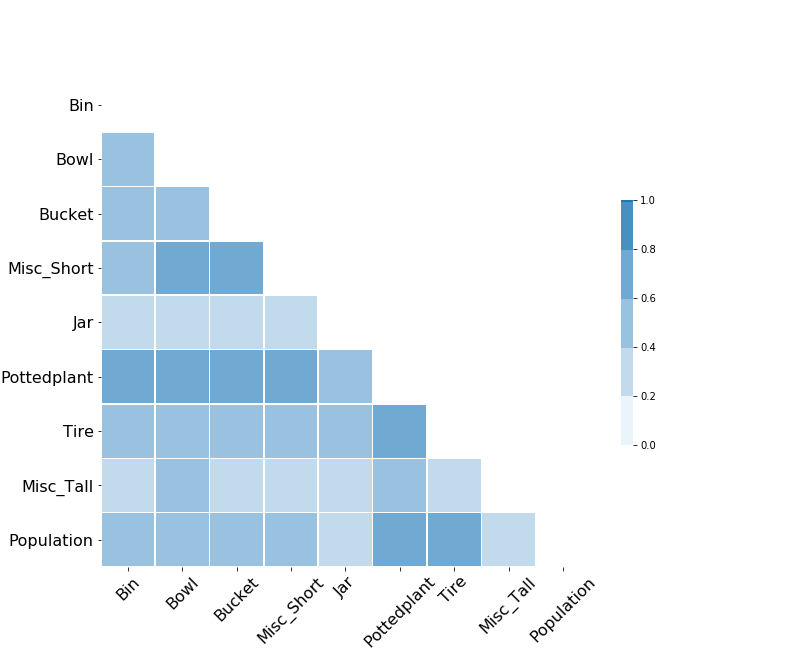

Supplement: S32 Fig — (TIF) [file pntd.0009122.s032.tif]

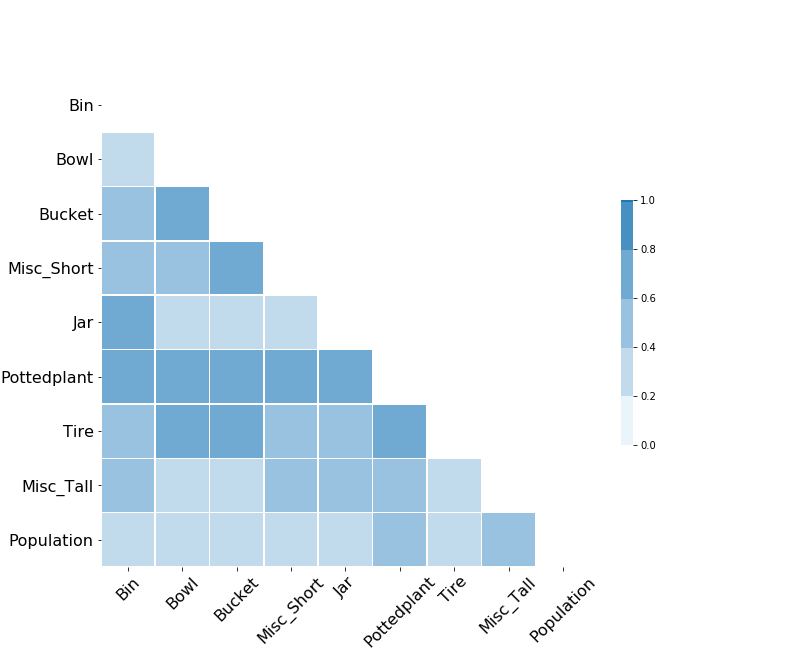

Supplement: S33 Fig — (TIF) [file pntd.0009122.s033.tif]

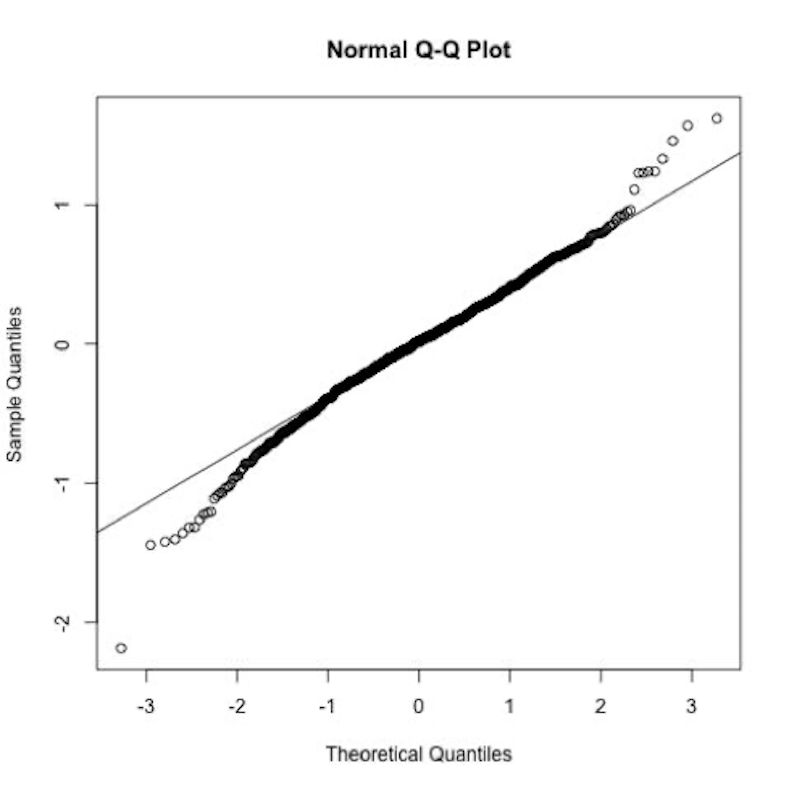

Supplement: S34 Fig — (TIF) [file pntd.0009122.s034.tif]

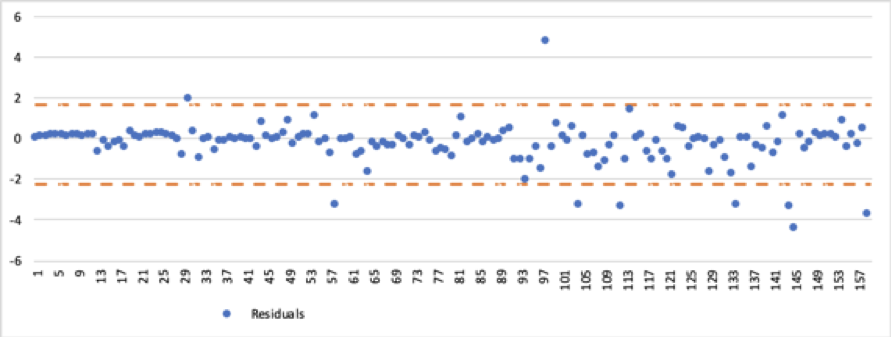

Supplement: S35 Fig — (TIF) [file pntd.0009122.s035.tif]

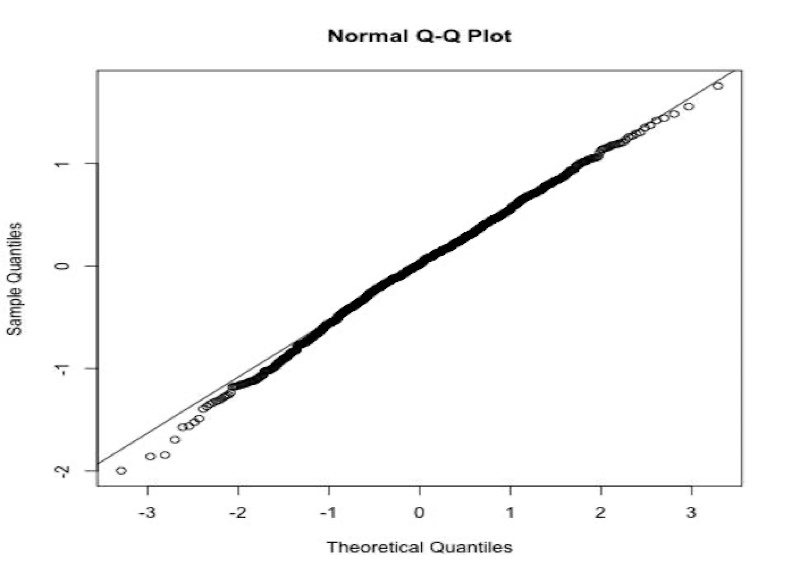

Supplement: S36 Fig — (TIF) [file pntd.0009122.s036.tif]

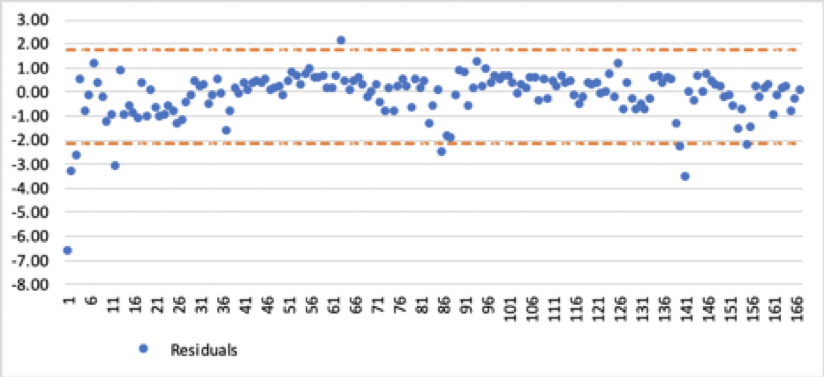

Supplement: S37 Fig — (TIF) [file pntd.0009122.s037.tif]

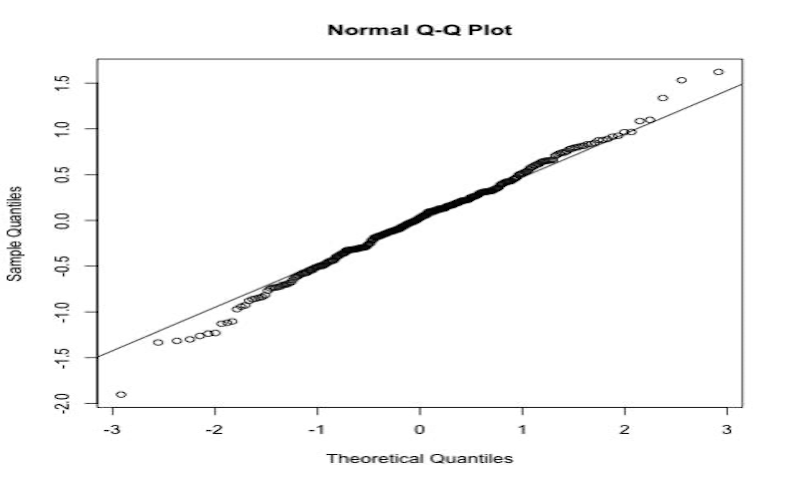

Supplement: S38 Fig — (TIF) [file pntd.0009122.s038.tif]

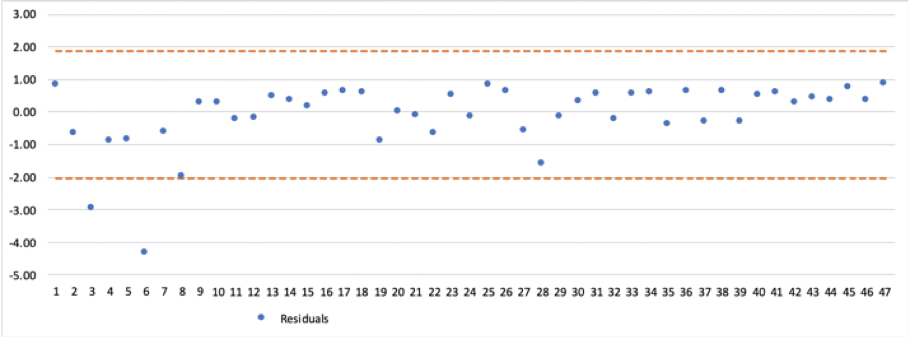

Supplement: S39 Fig — (TIF) [file pntd.0009122.s039.tif]
