# Supplementary material for: Added-value of mosquito vector breeding sites from street view images in the risk mapping of dengue incidence in Thailand
Source: PLoS Negl Trop Dis. 2021 Mar 8;15(3):e0009122. doi: 10.1371/journal.pntd.0009122 (PMC7971869; doi:10.1371/journal.pntd.0009122)
Supplement: S1 Table — (DOCX) [file pntd.0009122.s040.docx]

**Coefficients for random effect variables (Bangkok)**

| $code | (Intercept) |
| --- | --- |
| 100101 | -0.0938095 |
| 100102 | -0.4791048 |
| 100103 | -0.6784058 |
| 100104 | -0.4030202 |
| 100105 | -0.0281473 |
| 100106 | -0.589826 |
| 100107 | -0.0104109 |
| 100108 | -0.5078538 |
| 100109 | -0.6056362 |
| 100110 | -0.2498278 |
| 100111 | -0.409396 |
| 100112 | -0.4624696 |
| 100201 | 0.51757152 |
| 100202 | -0.0471575 |
| 100203 | 0.0128653 |
| 100204 | -0.0241291 |
| 100301 | 0.1950259 |
| 100302 | 0.36768217 |
| 100303 | -0.2778502 |
| 100304 | -0.3358233 |
| 100305 | 0.09751066 |
| 100306 | -0.0445589 |
| 100307 | -0.0230091 |
| 100401 | 0.06479381 |
| 100402 | -0.0230948 |
| 100403 | -0.1670565 |
| 100404 | -0.2774772 |
| 100405 | 0.0003171 |
| 100502 | -0.0841076 |
| 100508 | -0.4522533 |
| 100601 | 0.14435765 |
| 100608 | 0.420548 |
| 100701 | -0.064081 |
| 100702 | 0.24984299 |
| 100703 | 0.2481928 |
| 100704 | -0.1180455 |
| 100801 | 0.16138431 |
| 100802 | -0.1679904 |
| 100803 | -0.0329969 |
| 100804 | -0.2893381 |
| 100805 | -0.5026776 |
| 101001 | 0.07077412 |
| 101002 | 0.24444355 |
| 101102 | -0.030727 |
| 101103 | -0.0873696 |
| 101104 | 0.13935752 |
| 101105 | -0.4510197 |
| 101106 | -0.3127786 |
| 101203 | 0.04429303 |
| 101204 | 0.23450945 |
| 101301 | -0.4592883 |
| 101302 | 0.14700278 |
| 101303 | 0.05302854 |
| 101401 | -0.1908877 |
| 101501 | -0.1278221 |
| 101502 | -0.4622569 |
| 101503 | 0.0246328 |
| 101504 | 0.87381044 |
| 101505 | 0.08651073 |
| 101506 | 0.07418237 |
| 101507 | -0.2966929 |
| 101601 | 0.00267893 |
| 101602 | 0.06496593 |
| 101701 | 0.698818 |
| 101702 | 0.10242843 |
| 101704 | -0.0665161 |
| 101801 | -0.1195734 |
| 101802 | 0.26609598 |
| 101803 | 0.12696145 |
| 101804 | -0.060492 |
| 101901 | -0.3158949 |
| 101902 | 0.39113377 |
| 101904 | 0.32044535 |
| 101905 | 0.12503583 |
| 101907 | -0.09614 |
| 102004 | 0.38705698 |
| 102005 | 0.05945681 |
| 102006 | 0.23833159 |
| 102007 | 0.16410919 |
| 102009 | 0.40600597 |
| 102105 | -0.0762155 |
| 102107 | -0.4890875 |
| 102201 | 0.01600885 |
| 102202 | -0.0772919 |
| 102206 | -0.0329308 |
| 102207 | 0.32314028 |
| 102208 | 0.0918137 |
| 102209 | 0.20131698 |
| 102210 | -0.4825402 |
| 102302 | -0.152165 |
| 102303 | -0.0713663 |
| 102401 | 0.21008188 |
| 102402 | 0.01452157 |
| 102501 | 0.53570244 |
| 102502 | 0.42245021 |
| 102503 | 0.38923193 |
| 102504 | 0.32774213 |
| 102601 | -0.4025336 |
| 102701 | 0.28038547 |
| 102801 | -0.4914627 |
| 102802 | 0.07743597 |
| 102803 | -0.0340662 |
| 102902 | -0.4979407 |
| 103001 | 0.40150403 |
| 103002 | 0.08170244 |
| 103003 | 0.39546669 |
| 103004 | 0.61931711 |
| 103005 | 0.32128268 |
| 103101 | 0.0869856 |
| 103102 | -0.1978774 |
| 103103 | -0.1151688 |
| 103201 | 0.03158331 |
| 103203 | -0.1440798 |
| 103301 | 0.02588848 |
| 103302 | 0.16718283 |
| 103303 | 0.11319169 |
| 103401 | -0.0559196 |
| 103501 | -0.189025 |
| 103502 | -0.028274 |
| 103503 | 0.0742984 |
| 103504 | 0.36644472 |
| 103602 | 0.10375437 |
| 103604 | -0.1390231 |
| 103605 | -0.649774 |
| 103701 | 0.279507 |
| 103702 | 0.19118274 |
| 103703 | 0.15425856 |
| 103704 | 0.31083536 |
| 103801 | 0.05577399 |
| 103802 | 0.21335424 |
| 103901 | 0.70027119 |
| 103902 | 0.10533184 |
| 103903 | 0.72519656 |
| 104001 | 0.354238 |
| 104003 | 0.01536175 |
| 104004 | -0.0854128 |
| 104101 | -0.0294673 |
| 104102 | 0.04510097 |
| 104201 | -0.4814194 |
| 104202 | -0.3862373 |
| 104203 | -0.258955 |
| 104301 | 0.59765754 |
| 104302 | -0.6876971 |
| 104401 | 0.13491575 |
| 104501 | 0.92828425 |
| 104502 | -0.2304051 |
| 104503 | 0.18863002 |
| 104504 | 0.16290002 |
| 104601 | -0.1386306 |
| 104602 | -0.2268373 |
| 104603 | -0.1319202 |
| 104604 | 0.08395971 |
| 104605 | -0.2358794 |
| 104701 | -0.3043326 |
| 104801 | 0.17243063 |
| 104802 | -0.2144934 |
| 104901 | -0.1820371 |
| 104902 | -0.4567511 |
| 105001 | -0.0195533 |
|  |  |
| $Year_Season | |
|  | (Intercept) |
| 2015_D | -0.5425662 |
| 2015_ND | 2.0301696 |
| 2016_D | -2.320394 |
| 2016_ND | 0.8893416 |
| 2017_D | -0.1741473 |
| 2017_ND | 0.1175963 |
