# Supplementary material for: Added-value of mosquito vector breeding sites from street view images in the risk mapping of dengue incidence in Thailand
Source: PLoS Negl Trop Dis. 2021 Mar 8;15(3):e0009122. doi: 10.1371/journal.pntd.0009122 (PMC7971869; doi:10.1371/journal.pntd.0009122)
Supplement: S2 Table — (DOCX) [file pntd.0009122.s041.docx]

**Table S-2. Coefficients for random effect variables (Nakhon Si Thammarat)**

| $code | (Intercept) |
| --- | --- |
| 800101 | 0.09833278 |
| 800102 | 0.24219387 |
| 800103 | -0.0874597 |
| 800106 | 0.02950752 |
| 800107 | 0.18005207 |
| 800108 | 0.06407592 |
| 800112 | -0.1636817 |
| 800113 | -0.0323964 |
| 800114 | 0.33911843 |
| 800115 | 0.24365163 |
| 800116 | 0.25352392 |
| 800118 | -0.0182923 |
| 800119 | -0.3661686 |
| 800120 | 0.08164431 |
| 800121 | 0.20236218 |
| 800122 | 0.13445212 |
| 800201 | 0.18791322 |
| 800202 | 0.0639395 |
| 800203 | 0.28210466 |
| 800204 | 0.12413048 |
| 800205 | 0.43992954 |
| 800301 | 0.23497217 |
| 800302 | 0.16575865 |
| 800303 | 0.01364633 |
| 800304 | -0.0633171 |
| 800305 | 0.24448918 |
| 800401 | 0.27882104 |
| 800403 | 0.03073758 |
| 800404 | -0.0383008 |
| 800405 | -0.0642061 |
| 800406 | 0.14977921 |
| 800407 | -0.010134 |
| 800409 | 0.16827713 |
| 800410 | 0.22793771 |
| 800415 | -0.2937442 |
| 800416 | 0.08345994 |
| 800501 | 0.04135707 |
| 800502 | 0.38249332 |
| 800503 | -0.038094 |
| 800504 | 0.131898 |
| 800505 | 0.02446927 |
| 800601 | 0.12043116 |
| 800603 | 0.05445717 |
| 800604 | 0.13636097 |
| 800605 | 0.10991211 |
| 800606 | -0.100463 |
| 800607 | 0.05758071 |
| 800610 | 0.26100906 |
| 800611 | 0.0424607 |
| 800612 | 0.12751612 |
| 800613 | -0.0221812 |
| 800701 | -0.2657748 |
| 800702 | -0.1910511 |
| 800703 | 0.02401073 |
| 800704 | -0.2810732 |
| 800705 | -0.3738594 |
| 800706 | -0.1113392 |
| 800707 | -0.0726228 |
| 800708 | -0.3757896 |
| 800709 | 0.12504053 |
| 800710 | -0.0520441 |
| 800711 | -0.1651777 |
| 800801 | -0.3619602 |
| 800802 | -0.280326 |
| 800803 | 0.10368758 |
| 800804 | 0.06257782 |
| 800806 | -0.0170973 |
| 800807 | -0.2112007 |
| 800809 | 0.20138407 |
| 800810 | 0.18467394 |
| 800811 | -0.2276129 |
| 800813 | 0.08511291 |
| 800901 | -0.0794485 |
| 800902 | 0.09515419 |
| 800903 | 0.05305946 |
| 800904 | 0.07129071 |
| 800905 | -0.0749103 |
| 800906 | -0.0151064 |
| 800907 | 0.10781504 |
| 800908 | -0.0715331 |
| 800909 | 0.09181006 |
| 800910 | -0.0286736 |
| 800911 | 0.12688994 |
| 800912 | 0.149765 |
| 800913 | 0.06964671 |
| 801001 | 0.43677546 |
| 801002 | 0.20976625 |
| 801003 | 0.586766 |
| 801101 | -0.1435191 |
| 801102 | -0.4705156 |
| 801103 | -0.4585622 |
| 801104 | -0.0751082 |
| 801105 | -0.1120285 |
| 801106 | -0.6213701 |
| 801107 | -0.3749701 |
| 801202 | -0.7027308 |
| 801203 | -0.0833355 |
| 801204 | -0.3485917 |
| 801205 | -0.2484555 |
| 801206 | -0.4102965 |
| 801207 | -0.1883577 |
| 801208 | -0.1383302 |
| 801209 | 0.03772203 |
| 801210 | -0.0650164 |
| 801211 | 0.26927273 |
| 801212 | -0.242762 |
| 801213 | -0.1354668 |
| 801214 | -0.3265015 |
| 801215 | 0.00706188 |
| 801216 | 0.14435085 |
| 801217 | -0.0744551 |
| 801218 | 0.03172123 |
| 801301 | -0.055642 |
| 801303 | -0.0684727 |
| 801304 | -0.1595617 |
| 801305 | 0.14234816 |
| 801306 | -0.1213282 |
| 801401 | 0.04375544 |
| 801402 | 0.02625248 |
| 801403 | -0.1533249 |
| 801404 | -0.1895914 |
| 801405 | -0.0760819 |
| 801406 | 0.03830987 |
| 801407 | -0.597375 |
| 801408 | -0.2683225 |
| 801409 | -0.3066012 |
| 801501 | 0.24636267 |
| 801502 | -0.2920938 |
| 801601 | 0.17511831 |
| 801602 | 0.16669364 |
| 801603 | 0.15977445 |
| 801604 | 0.08615368 |
| 801605 | 0.26189754 |
| 801606 | -0.2193731 |
| 801607 | -0.2426787 |
| 801608 | 0.03247445 |
| 801609 | -0.1475607 |
| 801610 | -0.0954749 |
| 801611 | 0.31566735 |
| 801701 | 0.29915239 |
| 801702 | 0.01965096 |
| 801703 | 0.04528733 |
| 801704 | 0.02003959 |
| 801801 | -0.3722232 |
| 801802 | -0.3018794 |
| 801803 | -0.1366437 |
| 801901 | -0.097072 |
| 801902 | 0.22766199 |
| 801903 | 0.15069154 |
| 801904 | 0.01132174 |
| 801905 | -0.0069928 |
| 801906 | 0.08537662 |
| 802001 | 0.37426447 |
| 802002 | 0.28809239 |
| 802003 | 0.18495065 |
| 802004 | 0.22391005 |
| 802101 | -0.2728183 |
| 802102 | 0.04646359 |
| 802103 | -0.1063663 |
| 802104 | -0.0627358 |
| 802201 | 0.1719469 |
| 802202 | 0.2224185 |
| 802203 | -0.0057249 |
| 802301 | -0.0716237 |
| 802302 | 0.49436047 |
| 802303 | 0.18041074 |
| 802304 | 0.13205634 |
|  |  |
| $Year_Season | (Intercept) |
| 2015_D | -0.0748497 |
| 2015_ND | 0.00281449 |
| 2016_D | -0.0412708 |
| 2016_ND | -0.0918342 |
| 2017_D | 0.02184434 |
| 2017_ND | 0.18329581 |
|  |  |
|  |  |
