# Supplementary material for: Added-value of mosquito vector breeding sites from street view images in the risk mapping of dengue incidence in Thailand
Source: PLoS Negl Trop Dis. 2021 Mar 8;15(3):e0009122. doi: 10.1371/journal.pntd.0009122 (PMC7971869; doi:10.1371/journal.pntd.0009122)
Supplement: S3 Table — (DOCX) [file pntd.0009122.s042.docx]

**Table S-3. Coefficients for random effect variables (Krabi)**

| $code | (Intercept) |
| --- | --- |
| 810103 | 0.43975508 |
| 810105 | 0.53492925 |
| 810106 | 0.92305158 |
| 810111 | 0.60128053 |
| 810115 | 0.67225249 |
| 810116 | 0.49547612 |
| 810117 | 0.57969704 |
| 810118 | 0.42644512 |
| 810201 | 0.14830031 |
| 810202 | 0.07702136 |
| 810203 | -0.7736361 |
| 810204 | -0.049395 |
| 810205 | -0.8419667 |
| 810206 | -0.4830106 |
| 810303 | 0.2509542 |
| 810304 | 0.069713 |
| 810305 | 0.1059896 |
| 810401 | 0.38603934 |
| 810402 | 0.34724262 |
| 810403 | 0.11073048 |
| 810404 | -0.4000207 |
| 810405 | 0.57418818 |
| 810406 | -0.2317454 |
| 810407 | 0.333733 |
| 810501 | -0.3027624 |
| 810503 | 0.01603655 |
| 810504 | 0.29114884 |
| 810505 | 0.48441249 |
| 810506 | 0.0115843 |
| 810507 | 0.10719652 |
| 810508 | 0.24960693 |
| 810509 | 0.32097736 |
| 810601 | -0.8733618 |
| 810602 | -0.7520112 |
| 810603 | -0.6538508 |
| 810604 | -0.3650831 |
| 810701 | -0.7636439 |
| 810702 | -0.0966743 |
| 810703 | 0.47696253 |
| 810704 | -0.2649413 |
| 810801 | -0.1476332 |
| 810803 | -0.3846327 |
| 810804 | -0.1396675 |
| 810805 | 0.15288445 |
| 810806 | -0.6575872 |
| 810807 | -0.2773524 |
| 810808 | -0.7286331 |
| $Year_Season | (Intercept) |
| 2015_D | 0.9509659 |
| 2015_ND | 0.2436338 |
| 2016_D | -0.1349918 |
| 2016_ND | -0.4325056 |
| 2017_D | -0.3027168 |
| 2017_ND | -0.3243855 |
